# Bioactive Magnesium Silicate Activating Myocardial Energy Metabolism For Infarcted Myocardium Repair

**DOI:** 10.1002/exp2.70161

**Published:** 2026-04-13

**Authors:** Zhibin Liao, Chen Qin, Erhong Song, Chengbin Ding, Zhixu Wang, Yan Sun, Chen Song, Junjie Liu, Jingge Ma, Hongjian Zhang, Leyu Wang, Chengtie Wu

**Affiliations:** ^1^ State Key Laboratory of High Performance Ceramics and Superfine Microstructure Shanghai Institute of Ceramics Chinese Academy of Sciences Shanghai P. R. China; ^2^ Center of Materials Science and Optoelectronics Engineering University of Chinese Academy of Sciences Beijing P. R. China; ^3^ Guangdong Provincial Key Laboratory of Construction and Detection in Tissue Engineering Biomaterials Research Center School of Biomedical Engineering Southern Medical University Guangzhou China; ^4^ Department of Anatomy School of Basic Medical Science Guangzhou Medical University Guangzhou Guangdong P. R. China

**Keywords:** bioenergy‐activating bioink, biomaterials, infarcted myocardium repair, magnesium silicate nanoparticles

## Abstract

The heart is a highly energy‐dependent organ, developing bioenergy‐activating biomaterials to activate myocardial adenosine triphosphate (ATP) production and restore dysregulated energy homeostasis is a promising solution for its functional recovery. Inorganic biomaterials with tunable properties may deliver multiple physicochemical cues for bioenergy‐activation. However, there is currently a lack of systematic design and study on inorganic biomaterials‐derived physicochemical cues for myocardial bioenergy‐activation. This study proposes a bioenergy‐activating bioink based on inorganic biomaterials. Through the dual design of the chemical composition and physical morphology cues, the bioinks containing magnesium silicate (MS) nanoparticles with different morphologies were developed, and the corresponding 3D bioprinted cardiac patches were prepared. It was found that the magnesium and silicon components of MS are well beneficial to ATP production and myocardial maturation. More importantly, the morphology of MS nanoparticles could regulate the mitochondria‐targeted effects after endocytosis and the dynamic stiffness of the hydrogel matrix, thus systematically modulating ATP production and myocardial function. Furthermore, the patches with MS nanotubes significantly promoted heart repair and functional recovery in both rat and minipig animal models. This study proposes a new bioink design strategy based on biocompatible inorganic biomaterials for bioenergy‐activation to promote heart repair, offering more potential avenues for the clinical treatment of damaged, complex tissue.

## Graphical abstract

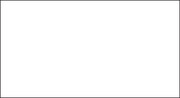

## 1 Introduction

Myocardial infarction (MI) caused by coronary artery occlusion is one of the main causes of human mortality [1]. The heart demands continuous production of large amounts of energy to maintain its rhythmical contraction function. MI causes a sudden suspension in the supply of oxygen and nutrients, resulting in a significant decrease in adenosine triphosphate (ATP) production [2]. Energy deficiency and metabolic disorders lead to irreversible necrosis of myocardial tissue, leading to catastrophic damage to the heart function [3]. Therefore, regulating the energy metabolism process in the myocardium is a feasible strategy for treating MI and promoting myocardial regeneration [4]. Recently, several bioenergy‐activating strategies, such as transplanting mitochondria and delivering energy metabolism activators, were developed to repair the myocardium, rescue heart failure, or treat other related mitochondrial dysfunction diseases [5, 6, 7]. However, their clinical application is limited due to low transplantation efficiency and the risk of affecting systemic metabolic homeostasis [8, 9].

The biomaterials‐based bioenergy‐activation strategy offers us a promising solution [10]. An increasing number of studies have found that the chemical composition of biomaterials, such as degradation products and ions, and physical properties, such as surface morphology and stiffness, can transmit specific stimulus signals to cells to regulate their energy metabolism process [11, 12, 13]. Inspired by this, it is feasible to comprehensively regulate the physicochemical properties of materials and design bioenergy‐activating biomaterials that can deliver multiple stimuli to activate myocardial energy metabolism and thus promote myocardial function. Inorganic biomaterials are attracting increasing attention for various tissue regenerative applications. The unique advantage of inorganic biomaterials is that they possess tunable properties that can deliver a variety of biochemical or biophysical cues to activate cell function [14]. In terms of chemical cues, inorganic biomaterials hold intrinsic bioactive ion‐releasing properties to facilitate tissue regeneration. Recent studies have shown that bioactive ions released from inorganic biomaterials can induce mitochondrial oxidative phosphorylation to improve ATP production [15, 16]. In terms of physical cues, their size and morphology can affect the cellular uptake process and organelle targeting, thereby regulating cellular biological processes and functions [17, 18]. Meanwhile, the physical cues can affect the mechanical properties of the hydrogel matrix, which is an important stimulus signal of cellular bioenergy‐activation [19]. Therefore, taking advantage of inorganic biomaterials to comprehensively modify physical and chemical cues to deliver bioenergy regulatory signals is a potential strategy for developing bioenergy‐activating biomaterials for infarcted myocardium repair.

**FIGURE 1:**
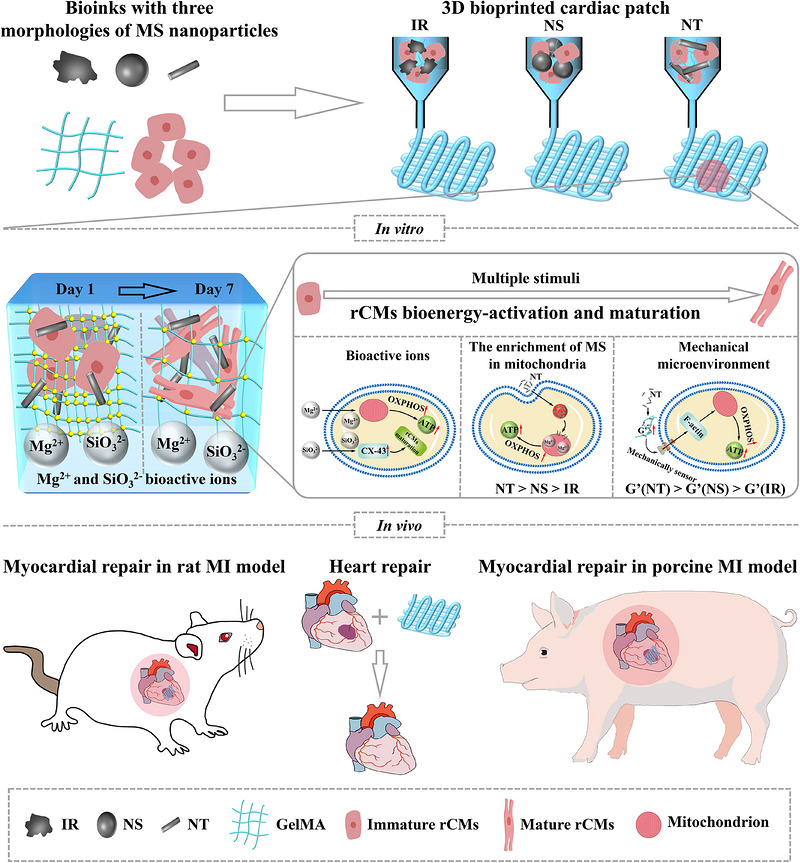
Schematic illustrating the morphology‐adjustable magnesium silicate (MS) nanoparticles‐based bioinks for activating myocardial energy metabolism and heart repair, attributed to the multiple bioenergy‐activation stimuli signals of ions and physical morphology (the enrichment effects of MS in mitochondria and the mechanical microenvironment of the hydrogel matrix). (IR: irregular particles, NS: nanospheres, NT: nanotubes).

Herein, we proposed a bioink design strategy based on magnesium silicate (MS) with different morphologies to activate the energy metabolism of cardiomyocytes (CMs) and promote myocardial function (Figure 1). Compared to previous design strategies of bioenergy‐activating biomaterials, the designed bioink integrates multiple stimuli signals of ions, physical morphology, and dynamic mechanics (Supplementary Data 1). In terms of chemical composition design, magnesium ions play an important role in maintaining mitochondrial function and cellular metabolic homeostasis [20]. Besides, silicon ions can protect CMs from the harsh microenvironment caused by MI [21]. Hence, the ionic products of MS are expected to be used as a functional component to modulate bioenergy metabolism and myocardial function. In terms of physical morphology design, we prepared MS nanoparticles with three different morphologies, including irregular nanoparticles (IR), nanospheres (NS) and nanotubes (NT) were developed, and the corresponding bioinks and 3D bioprinted cardiac patches were prepared. We found that the NT morphology of MS nanoparticles exhibited the most accumulation in the mitochondria after being taken up by CMs, thereby showing higher ATP production capacity. Meanwhile, according to the computational simulation and experimental analysis, the morphology (especially the NT morphology particles) adjusted the dynamic stiffness of the bioink, thereby indirectly affecting energy metabolism. Then, the in vivo repair effects of 3D bioprinted cardiac patches based on different morphologies of MS nanoparticles were investigated via the rat MI model, and the 3D bioprinted cardiac patch based on NT showed better repair effects. Finally, the 3D bioprinted cardiac patch based on NT was selected, and its effect of repairing myocardial tissue and enhancing the heart function was explored in minipigs. Notably, the therapeutic effects of our approach in minipigs have surpassed most of the treatment strategies reported in recent years (Supplementary Data 2). The conductive cardiac patches based on graphene oxide and carbon NT could achieve better therapeutic effects, but potential bio‐safety problems limited their application. Therefore, the bioink design strategy based on the biocompatible MS has significant clinical prospects for complex tissue regeneration.

## 2 Results and Discussion

### 2.1 Preparation and Characterization of MS Nanoparticles With Different Morphologies

**FIGURE 2:**
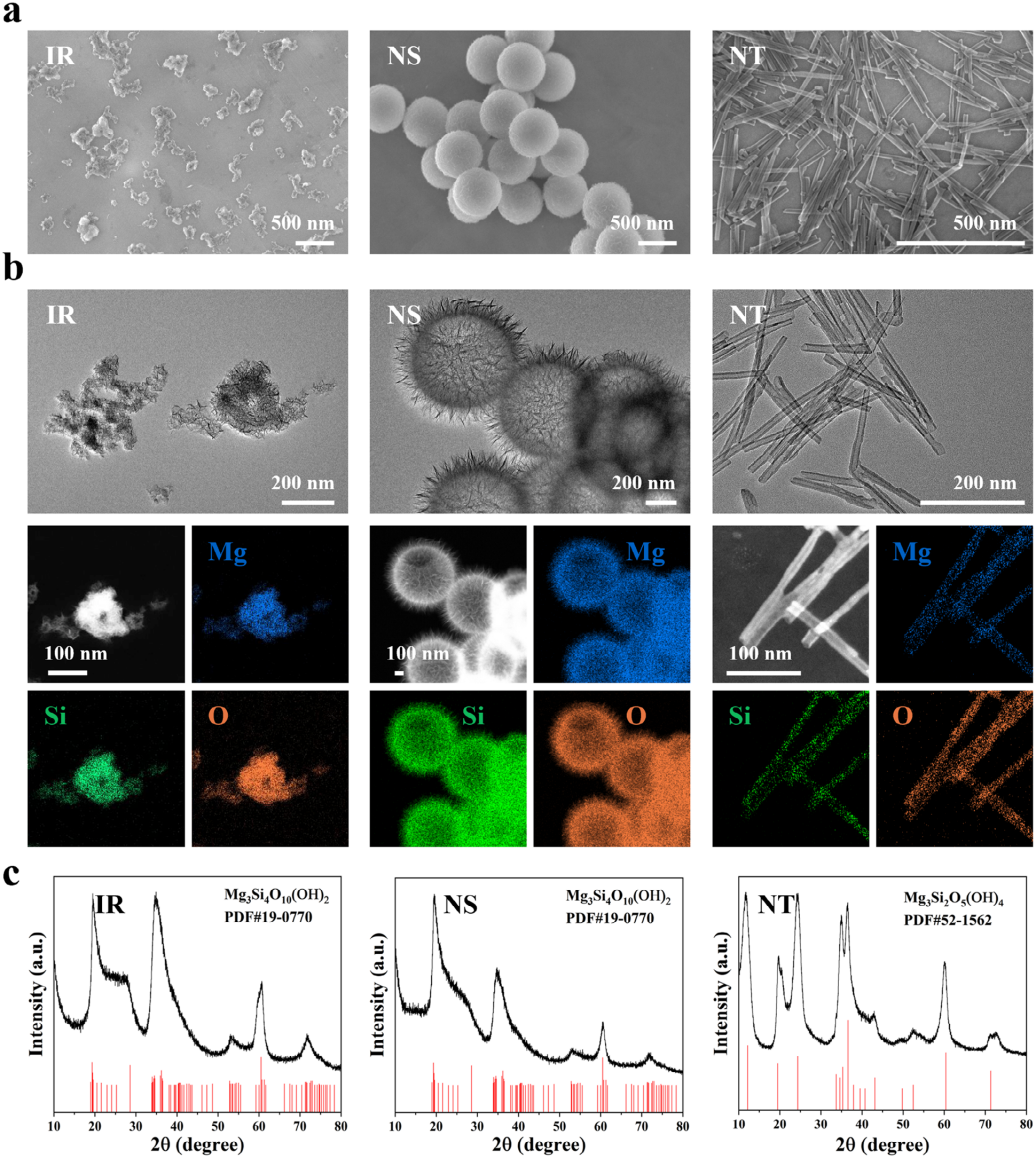
Characterization of magnesium silicate (MS) nanoparticles with different morphologies. (a) The SEM images of three MS nanoparticles with different morphologies, namely irregular particles (IR), nanospheres (NS), and nanotubes (NT); (b) the TEM images and Mg, Si, and O element distribution of three MS nanoparticles with different morphologies; and (c) the XRD patterns of three MS nanoparticles with different morphologies. MS nanoparticles with different morphologies were successfully prepared.

Firstly, MS nanoparticles with three different morphologies were successfully synthesized, namely irregular MS particles (IR), MS nanospheres (NS), and MS NT. Scanning electron microscopy (SEM) images of the three particles (Figure 2a) showed that the size of the IR ranged from tens of nanometers to hundreds of nanometers. The lengths of the NT were 100–500 nm, the width was about 10 nm. The diameter of the NS was about 500 nm. Further observation of the three morphologies of MS nanoparticles through transmission electron microscopy (TEM) revealed that both NS and NT had hollow structures (Figure 2b). Moreover, the uniform distribution of Mg, Si, and O elements inside the three nanoparticles was determined by energy‐dispersive spectroscopy (EDS). Then, the phases of the three MS nanoparticles were characterized by X‐ray diffraction (XRD). As shown in Figure 2c, the IR and NS could be indexed into the Mg_3_Si_4_O_10_(OH)_2_ (PDF#19‐0770) with low crystallinity. Meanwhile, the NT could be indexed into the Mg_3_Si_2_O_5_(OH)_4_ (PDF#52‐1562) with low crystallinity.

### 2.2 Fabrication and Characterization of Bioinks Containing MS Nanoparticles With Different Morphologies and the Corresponding 3D Printed Patches

After successfully synthesizing MS nanoparticles with different morphologies, we prepared inorganic‐organic composite bioinks composed of MS nanoparticles and gelatin methacryloyl (GelMA) hydrogel ink, and then fabricated the cardiac patches by 3D bioprinting technology, which could construct a biomimetic, native environment for implanted cells [22]. First, the appropriate concentration of MS nanoparticles in the GelMA ink for 3D printing CMs was investigated. Based on our previous research experience [23], NS were selected as a representative and were incorporated into the GelMA hydrogel in gradient proportions (0, 2%, 4%, and 6% relative to the GelMA mass) to prepare NS‐GelMA composite inks. Rheological analysis showed that the storage modulus (G′) of all bioinks was larger than their loss modulus (G″), indicating that all inks remain in a gel state at 16°C (Figure S1a). Furthermore, the viscosity test showed that all bioinks have excellent shear thinning properties, proving that they were suitable for extrusion 3D printing (Figure S1b). Subsequently, all bioinks were prepared as patches by 3D printing (Figure S1c). As shown in Figure S1d–f, NS‐incorporated patches possessed macro‐porous network structures similar to GelMA patches, and NS were uniformly distributed in the hydrogel matrix. Then, rat CMs (rCMs) were blended into the bioinks and 3D bioprinted as rCMs‐laden cardiac patches. As shown in their live/dead staining results, all encapsulated cells exhibited high viability in the 3D bioprinted cardiac patches (Figure S2) during the culture period of 7 days. Then, the synchronous contraction function of the 3D bioprinted cardiac patches with different MS concentrations was evaluated by a calcium transients test and immunostaining of cardiac‐specific proteins. As shown in Supplementary Video 1, the regular contraction of the entire patches started after 4 days of culture. After culturing for 7 days, synchronous contraction of the entire patches could be observed in the GelMA, 2NS‐GelMA, and 4NS‐GelMA groups. However, only slow contraction could be observed in the 6NS‐GelMA group. As shown in Figure S3a,d and Supplementary Video 2, the cardiac patches in the GelMA, 2NS‐GelMA, and 4NS‐GelMA groups occurred synchronous Ca^2+^ spikes accompanied by rhythmic Ca^2+^ flows after 4 days of culture. After 7 days of culture, the patches showed better synchronous Ca^2+^ spikes and rhythmic Ca^2+^ flows, especially the 2NS‐GelMA and 4NS‐GelMA patches exhibited the best synchronicity and rhythmicity. The related statistical results in Figure S3b–c, e–f showed that the time to reach the calcium peak in 2NS‐GelMA and 4NS‐GelMA groups was significantly less than that in the GelMA group. Meanwhile, more calcium spikes existed in the 2NS‐GelMA and 4NS‐GelMA groups. Furthermore, from the immunostaining images of cardiac‐specific proteins and their statistical results (Figure S3g–h), it can be seen that more gap junction protein connexin 43 (CX‐43) was expressed in the 2NS‐GelMA and 4NS‐GelMA groups, which is conducive to the consistent contraction of CMs [24]. Overall, the above results indicated that appropriate NS addition (2%–4%) enhanced the frequency and rhythmicity of Ca^2+^ flow in rCMs and improved the maturity and synchronous contraction function of the cardiac patches.

**FIGURE 3:**
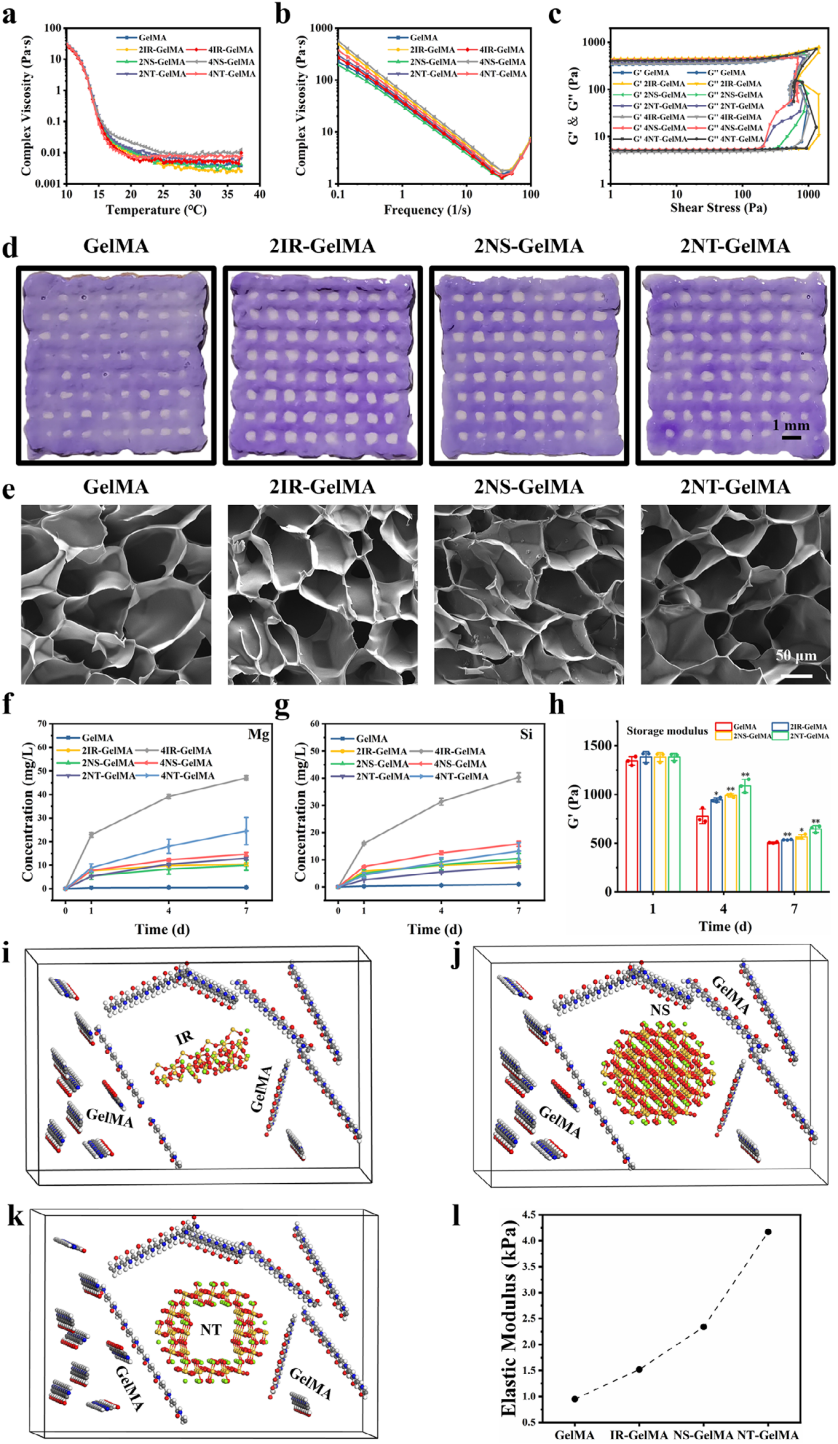
Characterization and theoretical computation of bioinks and 3D printed patches containing MS with different morphologies. (a) The viscosity of the composite bioinks containing the different morphologies of MS nanoparticles under the temperature range of 10–37°C; (b) the viscosity of the composite bioinks containing the different morphologies of MS nanoparticles under the shear rates range of 0.1–100 s^−1^; (c) the storage modulus (G′) and loss modulus (G′′) of the composite bioinks containing the different morphologies of MS nanoparticles at 16°C; (d) the 3D printed patches prepared by the bioinks containing MS with different morphologies (all patches were stained with crystal violet); (e) the SEM images of 3D printed patches; (f) the Mg ion release profile of 3D printed patches (*n* = 3); (g) the Si ion release profile of 3D printed patches (*n* = 3); (h) the storage modulus (G′) of the 3D printed patches during the culture (*n* = 3). The interactions between GelMA molecular fragments and IR (i), NS (j) and NT of MS nanoparticles (k); and (l) distribution of the theoretical computational elastic modulus of GelMA molecular fragments influenced by different morphologies of MS. *p < 0.05, **p < 0.01, or ***p < 0.001. Bioinks containing MS nanoparticles with different morphologies and the corresponding 3D printed patches that exhibited desirable ion release and unique dynamic mechanical properties were successfully prepared.

Therefore, we subsequently prepared the bioinks containing three different morphologies of MS nanoparticles at concentrations of 2% and 4% and tested their rheological properties. As shown in the rheological analysis (Figures 3a–c), all bioinks exhibited excellent temperature sensitive properties, shear thinning properties and similar G′ and G″, proving that they were suitable for extrusion 3D printing. Following that, all bioinks were prepared as patches by 3D printing (Figure 3d and Figure S4). All patches possessed similar macro‐porous network structures (Figure 3e and Figure S5). As shown in the Mg and Si ions release profiles (Figures 3f–g), at the concentration of 2%, the ion release patterns of all bioinks are similar. However, at the concentration of 4%, the ion concentrations released from the 4IR‐GelMA bioinks were much higher than those of the other two groups. This might be due to that IR possessed a distinct fluffy flake‐like layered structure, which resulted in a larger surface area compared to the other two particles, thereby leading to a higher ion release rate in the early period of 1 day for both the 2IR‐GelMA and 4IR‐GelMA bioinks [25]. In comparison to the 2IR‐GelMA bioinks, a more sufficient amount of ions could be released by the 4IR‐GelMA bioinks, thus still demonstrating a high ion release rate at day 4 and day 7.

In addition, we found that the G′ of all patches exhibited an interesting regularity during the culture (Figure 3h). First, the G′ of all patches decreased with the culture prolonging, which was attributed to the degradation of the hydrogel and the collapse of the polymer network [26]. However, in the MS‐incorporating groups, the trend of decreasing G′ was suppressed. It might be because that the MS nanoparticles interacted with the polymer chains, such as winding, thereby delaying the collapse of the polymer network [27]. Among the three MS‐incorporating groups, NT‐containing patches showed the highest G′, and the possible reason was their smaller size and longer aspect ratio leading to more interaction with the polymer chains. Moreover, computational simulation were used to explore the specific mechanism. Simulations were performed to investigate the interaction mechanism between MS nanoparticles and GelMA (Figures 3i–k). The elastic modulus of the composite material was primarily determined by the mechanical reinforcement provided by MS and its interaction with the GelMA matrix. The integration of MS can introduce variations in stiffness and flexibility, thereby governing the overall mechanical properties of the composite. From the microscopic viewpoint, the degree of interaction between MS and GelMA played a crucial role in determining the composite's stiffness. As shown in Figure 3l, the theoretical computational elastic modulus of GelMA, IR‐GelMA, NS‐GelMA, and NT‐GelMA varied significantly. GelMA fragments alone had an elastic modulus of approximately 0.95 kPa. Upon integrating MS with different morphologies, the elastic modulus increased significantly, with IR‐GelMA reaching about 1.52 kPa, NS‐GelMA reaching about 2.34 kPa, and NT‐GelMA reaching approximately 4.17 kPa. This clearly demonstrated the substantial impact of MS morphology on the mechanical properties of GelMA composites. The NT of MS exhibited the highest enhancement in the elastic modulus due to their efficient stress distribution and effective load transfer. This significant impact was attributed to the structural alignment of the NT, which facilitated better reinforcement within the GelMA matrix. The ability of the NT to distribute stress uniformly and transfer load effectively made them particularly advantageous for improving the stiffness of the composite material. In contrast, NS showed a moderate enhancement in the elastic modulus. The spherical structure of NS allowed for a balanced distribution of stress, providing a moderate level of reinforcement. Meanwhile, the IR of MS, with its non‐uniform structure, resulted in a different stress distribution pattern, contributing to varying degrees of stiffness improvement. The findings revealed a clear correlation between the morphology of MS, the stress distribution within the GelMA matrix, and the resulting elastic modulus. The more effective the load transfer from MS to GelMA, the higher the elastic modulus of the composite material becomes, which led to an enhanced interaction between MS and the polymer chains, ultimately inhibiting the degradation of the polymer network and resulting in the 3D printed patch exhibiting unique dynamic mechanical properties (Figure 3h).

### 2.3 In Vitro Physiological Functions of 3D Bioprinted Cardiac Patches Based on Different Morphologies of MS Nanoparticles

**FIGURE 4:**
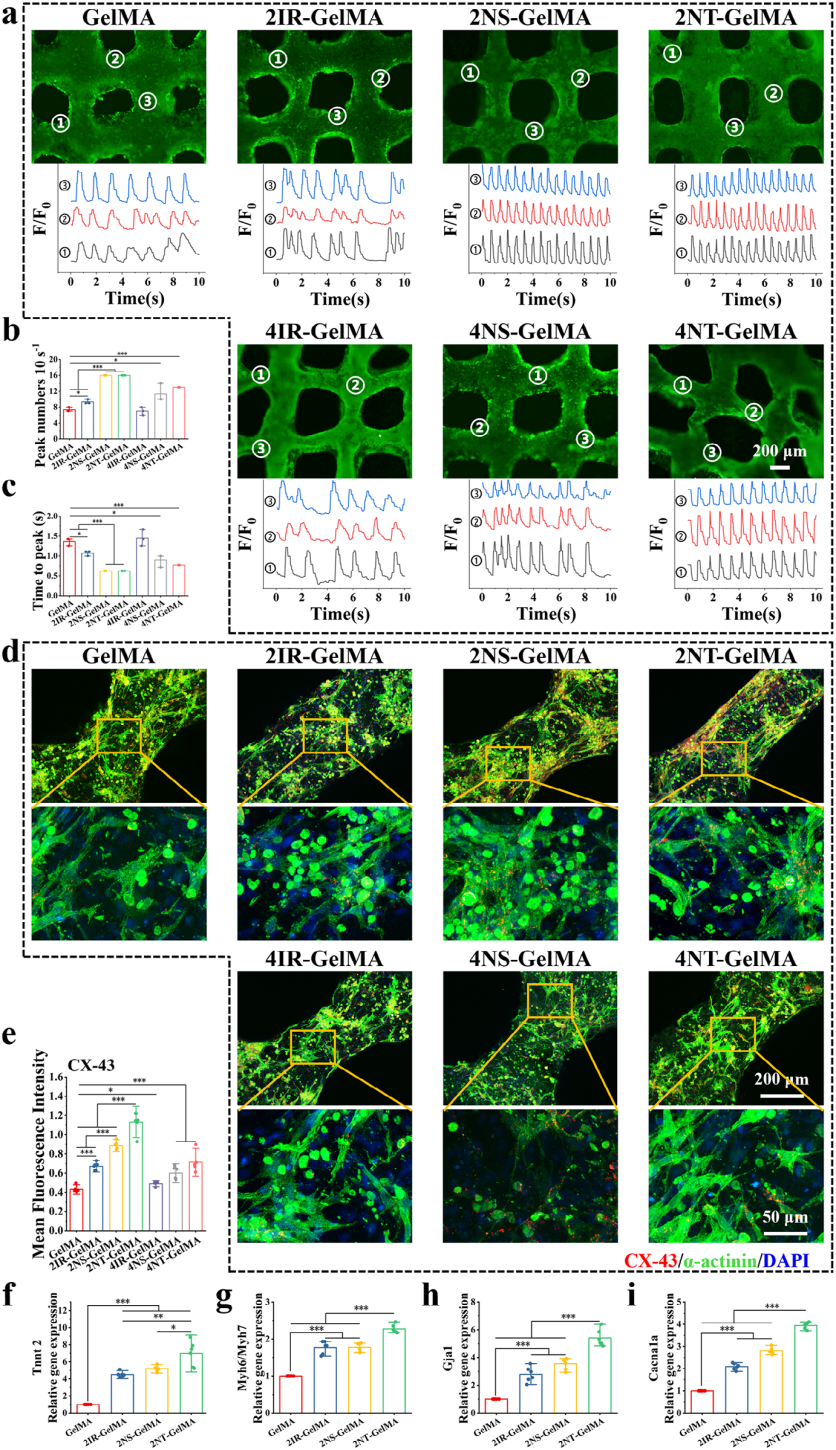
The in vitro physiological functions of 3D bioprinted cardiac patches with different morphologies of MS nanoparticles after 7 days of culture. (a) Calcium transient images and Ca^2+^ flow signals extracted from three different spots of the cardiac patches with 2% and 4% IR, NS, and NT; (b) the corresponding quantitative results of peak number per 10 s (*n* = 3); (c) time to peak according to the calcium‐transient signals (*n* = 3); (d) the immunostaining images of cardiac‐specific proteins connexin43 (CX‐43, red) and α‐actinin (green) of the cardiac patches with 2% and 4% IR, NS, and NT; (e) the semi‐quantitative statistical result of the CX‐43 expression after 7 days of culture (*n* = 6). The relative expression of cardiac‐specific genes, including: (f) Tnnt2; (g) Myh6/Myh7; (h) Gja1; and (i) Cacna1a of rCMs within these 3D bioprinted cardiac patches (*n* = 6). *p < 0.05, **p < 0.01, or ***p < 0.001. Different concentrations and morphologies of MS nanoparticles showed a significant regulatory effect on the in vitro physiological function of the 3D bioprinted cardiac patches, among which NT exhibited the most significant promoting effects.

Seven kinds of cardiac patches were subsequently prepared by printing rCMs with the bioinks containing different morphologies of MS nanoparticles at concentrations of 2% and 4%. The in vitro physiological effects were further investigated. Firstly, the synchronous contraction function of the cardiac patches with different morphologies of MS was evaluated. As shown in the microscopic video of the patches, regular contraction could be observed in all patches from the fourth day of culture (Supplementary Video 3), and several rhythmic Ca^2+^ flows could be observed (Figure S6a, Supplementary Video 4). The number of calcium spikes and the time to reach the calcium peak showed no significant difference between the MS‐containing groups and the GelMA groups (Figure S6b–c). After 7 days of culture, synchronous contraction with strong pulsation of the entire patches appeared (Supplementary Video 5). Moreover, the patches in the 2NS‐GelMA, 2NT‐GelMA, and 4NT‐GelMA groups exhibited synchronous Ca^2+^ spikes accompanied by rhythmic Ca^2+^ flows with high frequency (Figure 4a, Supplementary Video 6). The related statistical results in Figure 4b showed that the peak numbers of Ca^2+^ flow in patches containing NS (2NS‐GelMA, 4NS‐GelMA) and NT (2NT‐GelMA, 4NT‐GelMA) were significantly more than those in GelMA patches and patches containing IR. The low activities of the 4IR‐GelMA patches may be due to the high concentration of magnesium and silicate ions released by the 4IR‐GelMA bioink in the first 4 days (Figures 3f–g) [28]. Similarly, patches containing NS and NT also needed less time to reach the calcium peak (Figure 4c). Moreover, 2NS‐GelMA and 2NT‐GelMA showed the best synchronous contraction, which is similar to myocardial beating.

Besides, the maturity of the rCMs inside the patches was evaluated by immunostaining of cardiac‐specific proteins. After 4 days of culture, the rCMs could migrate to the surface of the patches and gradually spread out. Some spreading rCMs with obvious sarcomere structures could be observed on the surface of the patches (Figure S6d). Notably, the rCMs in the 2NT‐GelMA, 2NS‐GelMA, and 4NT‐GelMA groups were more spread out and had obvious sarcomere structures, while more rCMs in the 2IR‐GelMA and 4IR‐GelMA groups were in spherical morphology. This indicated that the rCMs in the patches containing NT and 2% NS had a more mature phenotype. Besides, after 4 days of culture, the patches containing NS and NT expressed more CX‐43 protein compared to the GelMA and IR‐containing patches (Figure S6e). Furthermore, the cellular networks appeared after 7 days of culture and almost covered the whole patches (Figure 4d). Dense distributions of spreading rCMs could be observed on the surfaces. In particular, the three groups of patches containing 2% MS nanoparticles significantly promoted the spreading and CX‐43 expression of rCMs (Figure 4e). Meanwhile, MS with different morphologies showed different promoting effects at a concentration of 2%, with the 2NT‐GelMA group exhibiting the best, the 2NS‐GelMA group being the second, and the 2IR‐GelMA group being the worst. To further explore the effect of MS morphology on the physiological function of the patches, the cardiac‐specific gene expressions (Tnnt2, Myh6/Myh7, Gja1, and Cacna1a) and functional protein expression levels (α‐actinin and CX‐43) of rCMs in the patches were analyzed. As shown in Figures 4f–i and Figure S7a–b, the incorporation of MS obviously promoted the expression of cardiac‐specific genes and functional proteins. Among the three MS‐incorporating groups, NT‐containing patches showed the highest expression, followed by the NS‐containing patches, and the IR‐containing patches showed the lowest. As shown in Figure S8, at the 4% concentration, the NT‐containing group significantly enhanced the expression of cardiac‐specific genes. However, the IR‐containing and NS‐containing groups showed no significant effect on Cacna1a.

In a word, compared with the GelMA patches, the patches with MS nanoparticles incorporated showed improved physiological functions. The promotion effects were better when the addition amount was 2% than when the addition amount was 4% in general. Especially, the promotion effect of MS with different morphologies on synchronous contraction and myocardial maturation followed the rule that NT was the best, NS was the second, and IR was the lowest. The above results demonstrated that the concentration and morphology of MS do play a role in regulating the maturation and physiological function of 3D bioprinted cardiac patches in vitro. In order to explore whether these regulatory effects of MS are through the modulation of energy metabolism, we then studied the effects of MS with different morphologies on energy metabolism in CMs and analyzed the underlying mechanism.

### 2.4 Energy Metabolism Effects and Mechanisms of MS Morphologies Regulating the Function of 3D Bioprinted Cardiac Patches

**FIGURE 5:**
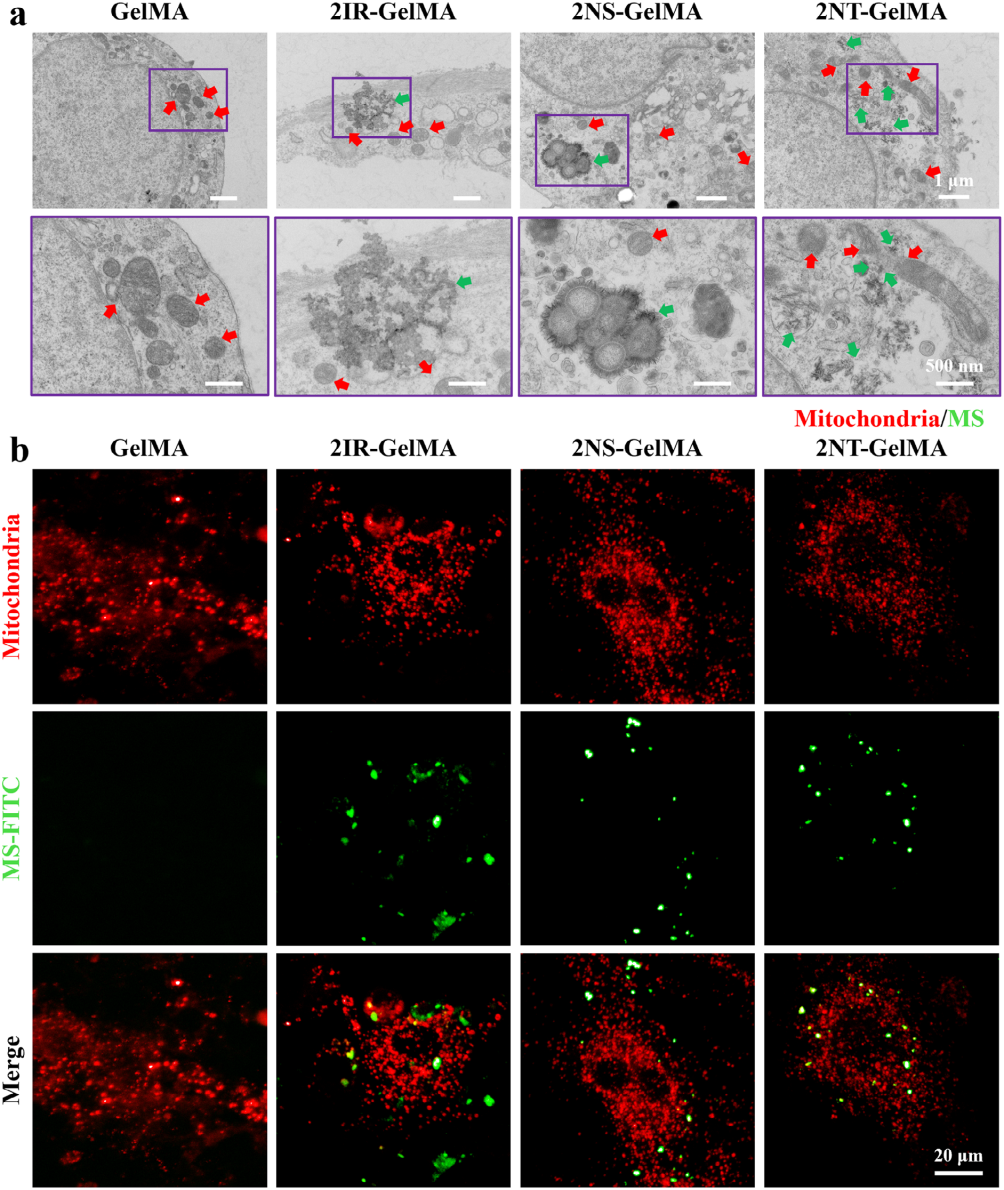
Co‐localization of MS nanoparticles with different morphologies and mitochondria in 3D bioprinted cardiac patches. (a) The TEM images of the section of the cardiac patches showing the inner‐cellular distribution of the MS nanoparticles with different morphologies. Green arrows: the location of MS nanoparticles. Red arrows: the location of mitochondria; and (b) the co‐localization images of the mitochondria (red) and the MS nanoparticles (green) in cardiac patches. Different morphologies of MS regulated the mitochondrial targeting and enrichment effects. More NT of MS targeted mitochondria after being up‐taken by rCMs in the 3D bioprinted cardiac patches.

**FIGURE 6:**
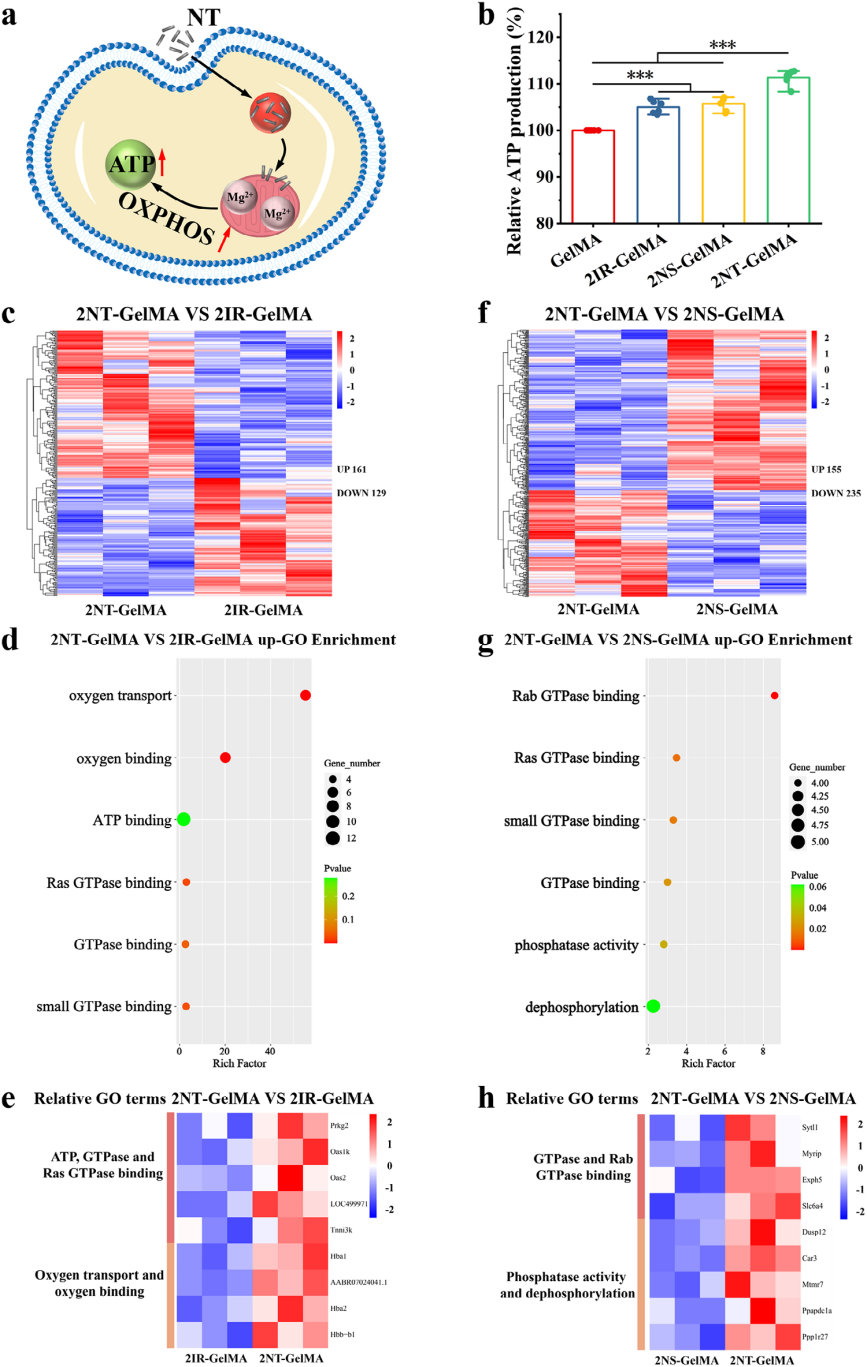
The production of adenosine triphosphate (ATP) and RNA‐Sequence analysis comparing the 2NT‐GelMA group and the 2IR‐GelMA, 2NS‐GelMA groups of the rCMs in the 3D bioprinted cardiac patches after 7 days of culture. (a) Schematic diagram of NT promoting the production of ATP; (b) relative ATP production levels of the cardiac patches after 7 days of culture (*n* = 6); (c) the cluster heatmap of the differentially expressed genes (DEGs) between the 2NT‐GelMA group and the 2IR‐GelMA group; (d) significant up‐GO terms enriched by the up‐regulated genes of 2NT‐GelMA versus 2IR‐GelMA; (e) heatmap of gene expression levels related to the GO terms of ATP binding, GTPase binding, Ras GTPase binding, oxygen transport and oxygen binding (2NT‐GelMA versus 2IR‐GelMA); (f) the cluster heatmap of DEGs between the 2NT‐GelMA group and the 2NS‐GelMA group; (g) significant up‐GO terms enriched by the up‐regulated genes of 2NT‐GelMA versus 2NS‐GelMA; and (h) heatmap of gene expression levels related to the GO terms of GTPase and Rab GTPase binding, phosphatase activity and dephosphorylation (2NT‐GelMA versus 2NS‐GelMA). *p < 0.05, **p < 0.01, or ***p < 0.001. Different morphologies of MS regulated ATP production and the expression of mitochondria metabolism‐related genes, among which NT exhibited the most significant promoting effect.

Firstly, TEM was used to observe the intracellular distribution of MS nanoparticles after they were up‐taken by cells in the 3D bioprinted cardiac patches. As shown in Figure 5a, all three morphologies of MS nanoparticles could be taken up by rCMs. Interestingly, all three MS nanoparticles showed enrichment near the mitochondria, an organelle that is closely related to cellular energy metabolism. We noticed that more NT were close to the mitochondria and some were distributed on the mitochondria. Then, MS nanoparticles and the mitochondria were labeled with different fluorescent markers to evaluate their co‐localization in rCMs. Several IR and NT were located in the mitochondria, while NS were mainly around the mitochondria (Figure 5b). For IR, it may be due to their irregular morphology that some smaller IR may target the mitochondria. Meanwhile, compared with NS, NT have a higher aspect ratio, so their shorter ends may be easier to target the mitochondria [29]. Pearson's correlation coefficient related to their co‐localization was statistically analyzed to show that NT co‐localized most with the mitochondria (Figure S9). These results suggest that the morphology of MS nanoparticles (especially the NT particles) affects their mitochondrial targeting, which may lead to the enrichment of Mg^2+^ in the mitochondria, and then the bioactive ion microenvironment regulates mitochondrial oxidative phosphorylation (OXPHOS) and mitochondrial metabolism (Figure 6a) [30]. Subsequently, the production of ATP, which typically reflects the activities of mitochondrial metabolites [31], was further tested in the 3D bioprinted cardiac patches with different morphologies of MS. As shown in Figure 6b, the 2NT‐GelMA group exhibited the highest ATP production level. Mitochondrial function in cardiac patches under hypoxic/low‐glucose conditions was evaluated via JC‐1 staining (Figure S10). Under normal conditions, JC‐1 forms red fluorescent aggregates in the mitochondria, while hypoxia/low‐glucose conditions cause their conversion to green fluorescent monomers. Notably, the MS‐incorporated cardiac patches, particularly the NT‐incorporated group, showed a higher JC‐1 aggregate/monomer ratio, indicating superior mitochondrial membrane potential and function. These findings demonstrated that the MS‐incorporated bioinks conferred cellular protection against hypoxic damage and improved mitochondrial function. Interestingly, analysis of mitochondrial OXPHOS‐related genes succinate dehydrogenase (SDH) and citrate synthase (CS) showed that MS‐incorporated cardiac patches significantly enhanced the expression of these genes, with the NT‐incorporated group showing the most pronounced effect (Figure S11). As the AMPK/PGC‐1α signaling pathway plays a key role in mitochondrial function activation [32, 33], we further conducted ELISA assays for p‐AMPK (phosphorylated AMPK) and PGC‐1α proteins. As shown in Figure S12, MS‐incorporated cardiac patches significantly activated the AMPK/PGC‐1α signaling pathway, with the NT group exhibiting the highest activation, further confirming the bioenergy‐activation effect of MS. Furthermore, transcriptomic sequencing was conducted on rCMs in the 3D bioprinted cardiac patches after 7 days of culture. The differentially expressed genes (DEGs) between the 2NT‐GelMA group and 2NS‐GelMA group, and between 2NT‐GelMA group and the 2IR‐GelMA group were analyzed. By comparing 2NT‐GelMA group and 2IR‐GelMA group, we found 161 up regulated and 129 down regulated DEGs (Figure 6c). The up regulated DEGs were enriched in several key gene ontology (GO) terms related to oxygen transport and binding, ATP binding, GTPase binding and Ras GTPase binding, which are closely related to cellular energy metabolism (Figures 6d–e) [34, 35, 36]. Then, by comparing 2NT‐GelMA group and the 2NS‐GelMA group, 155 up regulated and 235 down regulated DEGs were found (Figure 6f). Interestingly, the up regulated DEGs were also enriched in several key GO terms related to GTPase binding, Rab GTPase binding, phosphatase activity and dephosphorylation, which are closely related to cellular energy metabolism (Figures 6g–h) [36, 37].

Then, further analysis of the GO enrichment (2NT‐GelMA versus 2IR‐GelMA and 2NT‐GelMA versus 2NS‐GelMA) revealed that the up regulated DEGs were enriched in several key GO terms related to actin filament bundle assembly, cytoskeletal protein binding, cell projection assembly and cell projection organization, which are closely related to sensing the surrounding mechanical microenvironment (Figure S13a–d) [38, 39]. This was attributed to the dynamic mechanical environment of the patches caused by the incorporation of MS nanoparticles (Figures 3h–l). Moreover, the mechanical environment could regulate cellular energy metabolism through mechanotransduction pathways [12]. The mechanosensitive protein vinculin plays a critical role in the development and maturation of CMs [40], which is a potential pathway for activating cardiac energy metabolism. As shown in Figure S14–15, the MS‐incorporated cardiac patches significantly enhanced the protein and gene expression of vinculin, with the NT‐incorporated group displaying the highest level. This phenomenon may be attributed to the regulation of the G′ of GelMA hydrogels by MS morphology (Figures 3h–l). The incorporation of MS nanoparticles, particularly NT morphology, increased the G′ of GelMA, ultimately leading to the enhanced expression of the mechanosensitive protein Vinculin in the cardiac patches. These results suggested that the morphology of MS nanoparticles can regulate cellular energy metabolism by regulating the effect of mitochondrial targeting and the mechanical environment of the patches, with NT showing the best‐promoting effect. Meanwhile, cellular energy metabolism is closely related to the development of cardiac tissue [41]. Therefore, the regulatory effects on cellular energy metabolism can promote the maturation of 3D bioprinted cardiac patches.

Moreover, the results of transcriptomic sequencing for silicate‐containing groups (2IR‐GelMA, 2NS‐GelMA, and 2NT‐GelMA) and the GelMA group were analyzed. Principal component analysis (PCA) of the four groups (Figure S16a) revealed that the whole transcriptome of GelMA was obviously different from the three MS‐containing groups. 140 up regulated and 48 down regulated common DEGs were detected in the three MS‐containing groups compared to the GelMA group (Figure S16b). These results indicated that some similar cellular responses were triggered by all three MS nanoparticles. GO enrichment analysis showed that the common up regulated DEGs were enriched in several key GO terms related to cardiac development and maturation, cell‐extracellular matrix interactions, and G‐protein coupled receptor and peptide receptor activity (Figure S16c). Specifically, the cell‐extracellular matrix interactions are closely related to the maturation of CMs [42]. Besides, the G‐protein coupled receptors possessed the function of regulating myocardial function and protecting the myocardium [43]. Moreover, the heat map of the main up regulated DEGs in these GO terms was displayed in Figure S16d. Among the enriched genes in these GO terms, Nrg1 and Erbb4, which are beneficial for CM proliferation and heart repair [44], showed significant up regulation in all three MS‐containing groups. According to the above transcriptomic analysis, we may infer that the MS nanoparticles with three morphologies might commonly modulate the function of CMs by influencing the above biological functions. These modulating effects may be attributed to the magnesium and silicate ions released by MS nanoparticles. As shown in Figures 3f–g, the ion release profiles of 2IR‐GelMA, 2NS‐GelMA, and 2NT‐GelMA bioinks were similar. Previous studies have confirmed that silicate ions released by silicate biomaterials can improve the survival rate of CMs, effectively protect CMs, and inhibit their apoptosis by suppressing the phosphorylation of p38 mitogen‐activated protein kinase under low glucose/hypoxia conditions [45]. Besides, magnesium ions also play an important role in MI. Studies have found that magnesium supplementation can reduce myocardial oxygen consumption, enhance mitochondrial function, and protect CMs from the harsh microenvironment after MI [46, 47].

MS could target and stimulate mitochondria, activate their energy metabolism, and thus produce more ATP. Therefore, we speculate that the mechanism of the composition and morphology of MS regulating the physiological function of CMs can be attributed to the following reasons. Firstly, the microenvironment of bioactive ions constructed by MS can not only promote energy metabolism but also activate genes related to CM maturation. More importantly, the morphology of MS reflects the regulatory effect on energy metabolism. This can be attributed to two reasons. First, the morphology affects the enrichment effect of MS in mitochondria, thereby regulating mitochondrial oxidative phosphorylation process. Second, the morphology affects the dynamic mechanical microenvironment of the bioink, thereby regulating cellular energy metabolism through mechanical transduction pathways.

### 2.5 Repair Functions of 3D Bioprinted Cardiac Patches Based on MS Nanoparticles With Different Morphologies in Rat MI Models

**FIGURE 7:**
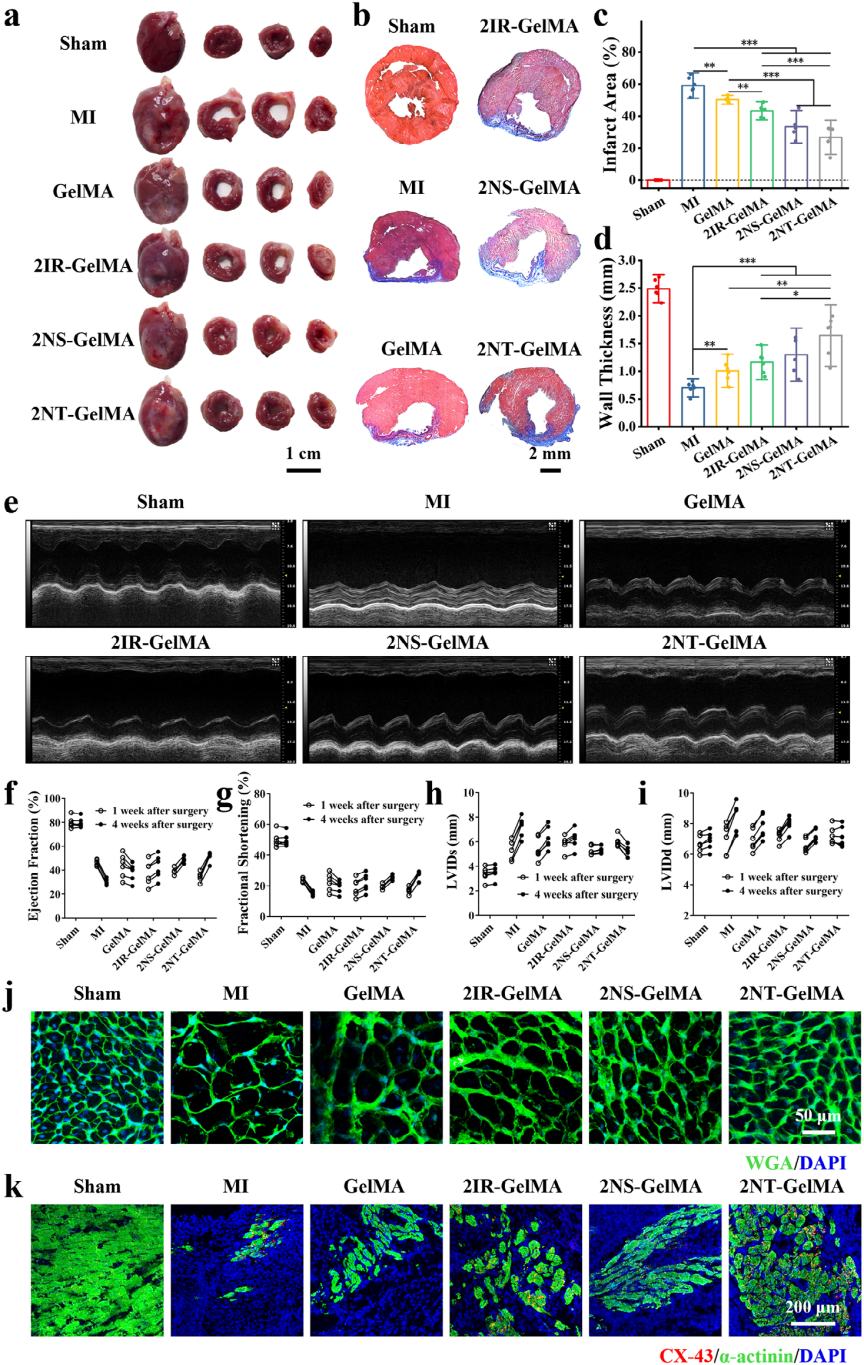
The in vivo repair effects of 3D bioprinted cardiac patches based on MS nanoparticles with different morphologies in rats. (a) Representative photos of the whole rat hearts and three cross sections of the hearts with different treatments at 4 weeks post‐surgery; (b) Masson's trichrome staining sections of the rat hearts with different treatments at 4 weeks post‐surgery (blue: fibrosis tissue; red: myocardium); (c) statistical result of the infarct area according to the Masson's trichrome staining images (*n* = 6); (d) statistical result of the infarct wall thickness according to the Masson's trichrome staining images (*n* = 6); (e) representative echocardiograms of rat hearts at 4 weeks post‐surgery; (f) quantitative results of the ejection fraction (EF); (g) fractional shortening (FS); (h) left ventricle internal diameter in systole (LVIDs); (i) left ventricle internal diameter in diastole (LVIDd) according to echocardiography results at 1 week and 4 weeks post‐surgery (*n* = 6); (j) Immunofluorescent staining images of wheat germ agglutinin (WGA) in the infarction regions of heart sections at 4 weeks post‐surgery (green: WGA, blue: DAPI); and (k) immunofluorescent staining images of CX‐43 and α‐actinin in the infarction regions of rat heart sections at 4 weeks post‐surgery (red: CX‐43, green: α‐actinin, blue: DAPI). *p < 0.05, **p < 0.01, or ***p < 0.001. The 3D bioprinted cardiac patches with different morphologies of MS nanoparticles enhanced the myocardium repair in rats, among which cardiac patches with NT‐morphology MS exhibited the most significant promoting effect.

The MI model was established by ligating the left anterior descending artery (LAD) in rats. 3D bioprinted cardiac patches containing 2% MS with three different morphologies were prepared and implanted on the infraction region. Meanwhile, the Sham group (thoracotomy without ligation) and the MI group (without patch implantation) served as control groups. After 4 weeks post‐surgery, all hearts were collected and cut to observe the morphology of the infarction area (Figure 7a). The cardiac patches implanted on the heart surface were almost completely degraded. To investigate this observation, we evaluated the in vitro degradation of GelMA, 2IR‐GelMA, 2NS‐GelMA, and 2NT‐GelMA hydrogels over one month. As shown in Figure S17, the hydrogels underwent minimal degradation at pH 8.5, with gradual degradation at pH 5.5, and underwent complete degradation before day 14 in the presence of Type II collagenase. No significant difference in degradation was observed among the hydrogel groups, which could be attributed to the low loading amount of the MS nanoparticles. The nearly complete degradation of the cardiac patches within one month aligns with these in vitro findings, considering the complex in vivo microenvironment. The gross photos and the Masson's Trichrome staining images (Figure 7b) of rat hearts with different treatments showed that the left ventricular wall of the MI group showed obvious dilatation and necrosis. The statistical results corresponding to the Masson's Trichrome staining images are shown in Figures 7c–d. Infarct areas in all patch‐implanted groups were smaller than the MI group. Meanwhile, compared with the GelMA group, the infarct areas in MS‐containing groups were significantly smaller, among which the 2NT‐GelMA group showed the smallest infarct area. A similar trend could be observed in the statistical results of ventricular wall thickness. The implantation of MS‐containing patches significantly inhibited ventricular wall thinning. Particularly, the 2NT‐GelMA patches showed the best effects on myocardial repair and ventricular wall thickening. In addition, the cardiac function recovery was evaluated by echocardiography. At one week post‐surgery, all ligation groups showed an infarct phenotype with no obvious contraction of the left ventricular wall (Figure S18a). After 4 weeks of implantation, the echocardiograms revealed that a contraction wave could be found in 2IR‐GelMA, 2NS‐GelMA, and 2NT‐GelMA groups (Figure 7e). In addition, the important parameters of the left ventricle for presenting cardiac functions were analyzed from echocardiograms. As shown in Figures 7f–g and Figure S18b, the three MS‐containing groups, 2IR‐GelMA, 2NS‐GelMA and 2NT‐GelMA, improved the ejection fraction (EF) and fractional shortening (FS). Among them, the 2NT‐GelMA group showed the most significant improvement. Besides, the implantation of 2NT‐GelMA patches clearly decreased the left ventricle internal diameter in systole (LVIDs) and left ventricle internal diameter in diastole (LVIDd), suggesting that 2NT‐GelMA patches were most beneficial to beating compliance of heart (Figures 7h–i, Figure S18b). Subsequently, the quality of myocardial repair after 4 weeks of implantation was further evaluated by histological staining. Immunofluorescent staining images of wheat germ agglutinin (WGA) in the infarction regions of heart sections showed that adverse cardiac hypertrophy could be observed in both the MI group and the GelMA group (Figure 7j, Figure S19a). In contrast, the implantation of MS‐containing patches suppressed hypertrophy, and the smallest size of CMs appeared in the 2NT‐GelMA group. Moreover, the α‐actinin and CX‐43 expression in the infarction regions were investigated. Compared with MI and GelMA groups, 2IR‐GelMA, 2NS‐GelMA, 2NT‐GelMA groups exhibited more α‐actinin‐positive myocardium and more CX‐43 expression (Figure 7k, Figure S19b–c). The 2NT‐GelMA group showed the highest functional myocardium coverage. Additionally, according to our earlier findings that MS is beneficial for angiogenesis [23], a key biological process in MI repair, we further investigated the vascular formation in the infarct region. As shown in Figure S20, many vessels, which were marked by von Willebrand factor (vWF) and α‐smooth muscle actin (α‐SMA), could be observed in 2IR‐GelMA, 2NS‐GelMA, and 2NT‐GelMA groups. The infarct region of the 2NT‐GelMA group generated the most dense microvessels, which is conducive to the repair of the myocardium. This improvement in vascularization can be explained in two ways. For one thing, MS can promote vascular formation by releasing magnesium and silicate ions. The beneficial effects of magnesium ions on vascularization in tissues, such as skin and bone, have been widely studied [48, 49]. Furthermore, Si ions can activate the KDR receptor to initiate the angiogenic pathway [50]. For another thing, CMs also closely interact with vascular endothelial cells. For instance, CMs can release the secretion of VEGF and Ang‐1 and activate angiogenesis [51, 52]. Therefore, rCMs in 2NT‐GelMA exhibited a more mature phenotype and physiological functions, thus promoting positive myocardial‐vascular interaction and inducing more vascular formation.

From the above results, we may conclude that the implantation of 3D bioprinted cardiac patches based on MS nanoparticles accelerated myocardium repair after MI in rats. Furthermore, their in vivo repair effects were regulated by the morphologies of MS. The promotional effects followed the rule that NT was the best, NS was the second, and IR was the lowest, which was consistent with the in vitro results. In a word, the morphologies of MS nanoparticles concurrently regulated the in vitro physiological functions and in vivo repair activities of the 3D bioprinted cardiac patches. In particular, 2NT‐GelMA patches possessed the best bioactivities and functions.

### 2.6 Repair Functions of 3D Bioprinted Cardiac Patches Based on NT‐Morphology MS in Porcine MI Models

**FIGURE 8:**
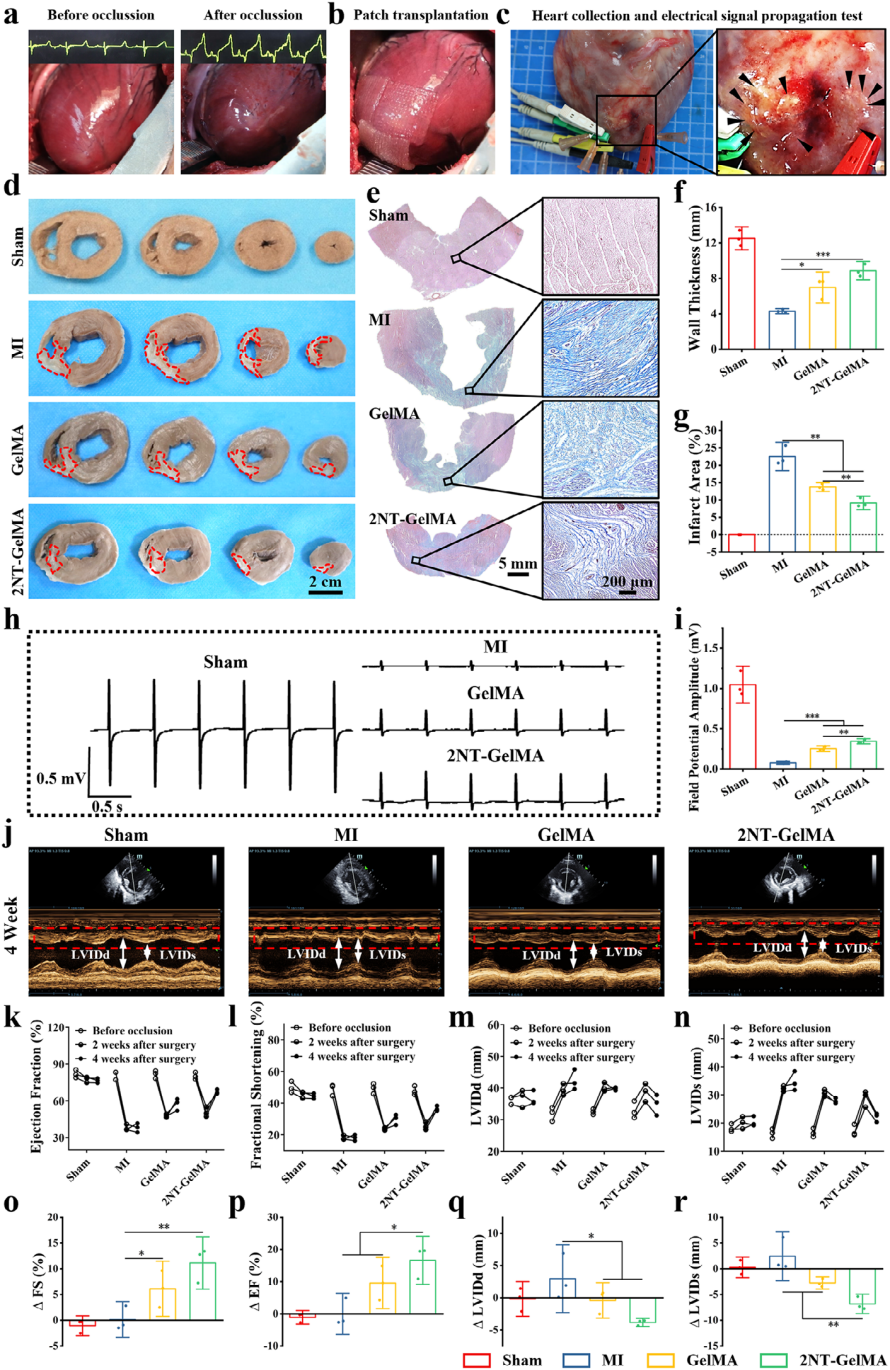
The in vivo repair effects of 3D bioprinted cardiac patches based on NT‐morphology MS nanoparticles in minipigs. (a) Representative photos and electrocardiogram patterns of the minipig hearts before and after the occlusion; (b) representative photos of the minipig heart after the transplantation of the cardiac patches; (c) representative photos of the ex vivo hearts for the assessment of electrical signal propagation at 4 weeks post‐surgery. Black arrows: residual undegraded patches; (d) transverse section photos of the hearts with different treatments from the apex to the ligation site at 4 weeks post‐surgery. The fibrotic regions were indicated by the circles with red dotted lines; (e) Masson's trichrome staining sections of the minipig hearts with different treatments at 4 weeks post‐surgery (blue: fibrosis tissue; red: myocardium); (f) statistical result of the infarct wall thickness according to the transverse section photos (*n* = 3); (g) statistical result of the infarct area according to the transverse section photos (*n* = 3); (h) representative electrical signal propagation patterns of the stimulation signal from ex vivo hearts; (i) statistical result of the amplitude of the local field potential according to the electrical signal propagation patterns (*n* = 3); (j) representative echocardiographic images of minipig hearts at 4 weeks post‐surgery; (k) quantitative results of the ejection fraction (EF); (l) fractional shortening (FS); (m) left ventricle internal diameter in diastole (LVIDd); (n) left ventricle internal diameter in systole (LVIDs), according to echocardiography results before occlusion and at 2 weeks and 4 weeks post‐surgery (*n* = 3); (o) statistical results of the changes of the EF; (p) FS; (q) LVIDd; and (r) LVIDs from 2 weeks to 4 weeks post‐surgery according to the echocardiography results (*n* = 3). *p < 0.05, **p < 0.01, or ***p < 0.001. The 3D bioprinted cardiac patches based on NT‐morphology MS significantly accelerated the myocardium repair and heart function recovery in minipigs.

Since the anatomical structure and beating characteristics of the pig heart are similar to those of the human heart [53], the in vivo repair effects of the 2NT‐GelMA patches were verified using a porcine MI model. For this purpose, human CMs (hCMs) differentiated from human‐induced pluripotent stem cells (hiPSCs) were used to prepare the 3D bioprinted cardiac patches [54]. Meanwhile, the Sham group (thoracotomy without ligation), the MI group (without patch implantation), and the GelMA patches implantation group served as control groups. As shown in Figure 8a and Supplementary Video 7, an infarct region with a dark purple color was caused by occlusion, along with the anteroseptal ST‐segment elevation on electrocardiograph patterns. This verified the formation of acute myocardial ischemia. After that, the 3D bioprinted cardiac patches were implanted into the infarct regions by using fibrin glue. The 3D bioprinted cardiac patch was firmly attached to the heart and completely covered the infarcted area (Figure 8b, Supplementary Video 8).

After 4 weeks of implantation, all minipig hearts were collected to evaluate their repair effects. As shown in Figure 8c, several residual undegraded patches could be observed on the heart surface, indicating the stable adhesion of the patches. The gross photos and the Masson's Trichrome staining images of the minipig hearts showed obvious fibrotic scars formed in the anteroseptal and anterior left ventricular walls of the MI group. On the contrary, implantation of the cardiac patches inhibited scar formation (Figures 8d–e). The 2NT‐GelMA group exhibited the most similar tissue morphology to the Sham group, with the thickest ventricular wall and smallest infarct size (Figures 8f–g). Moreover, ex vivo electrical signal propagation was investigated by testing the electrocardiography under specific electrical signal stimulation (Figure 8c). As shown in Figures 8h–i, the 2NT‐GelMA group produced a local field potential amplitude much stronger than the MI group, indicating the electroactivity, which is closely related to the recovery of cardiac function [55], was significantly enhanced by 2NT‐GelMA patches. Furthermore, the cardiac function was evaluated by echocardiography. By comparing the echocardiograms before surgery, 2 weeks post‐surgery, and 4 weeks post‐surgery, we found that 2NT‐GelMA patches enhanced the contraction activity of the left ventricular anterior wall (Figure S21, Figure 8j). Then, key parameters associated with cardiac function were analyzed from echocardiograms (Figures 8k–r). In the MI group, the FS and EF values of the left ventricle continued to decrease in the 4 weeks after surgery. In contrast, the 3D bioprinted cardiac patches inhibited the decrease of FS and EF in the first 2 weeks. In particular, 2NT‐GelMA patches significantly promoted the FS and EF from 2 weeks post‐surgery to 4 weeks post‐surgery in comparison to the MI and GelMA groups. Besides, the 2NT‐GelMA group exhibited significantly decreased LVIDs and LVIDd, indicating that 2NT‐GelMA patches were beneficial to recover the beating compliance of the heart. Moreover, the NT‐GelMA cardiac patch demonstrated superior efficacy compared to most reported therapeutic strategies (Supplementary Data 2). Compared to ionic cocktail injection therapy [56], the NT‐GelMA cardiac patch demonstrated superior improvement in cardiac function (EF and FS). This can be attributed to the combination of the chemical composition and physical morphology cues, integrated into functional 3D bioprinted cardiac patches to enable more effective repair of the infarcted myocardium. Although conductive and metabolically activated cardiac patches based on carbon NT and graphene oxide may achieve better therapeutic effects (EF and FS) [55, 57], potential biosafety concerns limit their clinical application. Therefore, the bioink design strategy based on biocompatible MS is clinically promising for regenerating complex tissues.

**FIGURE 9:**
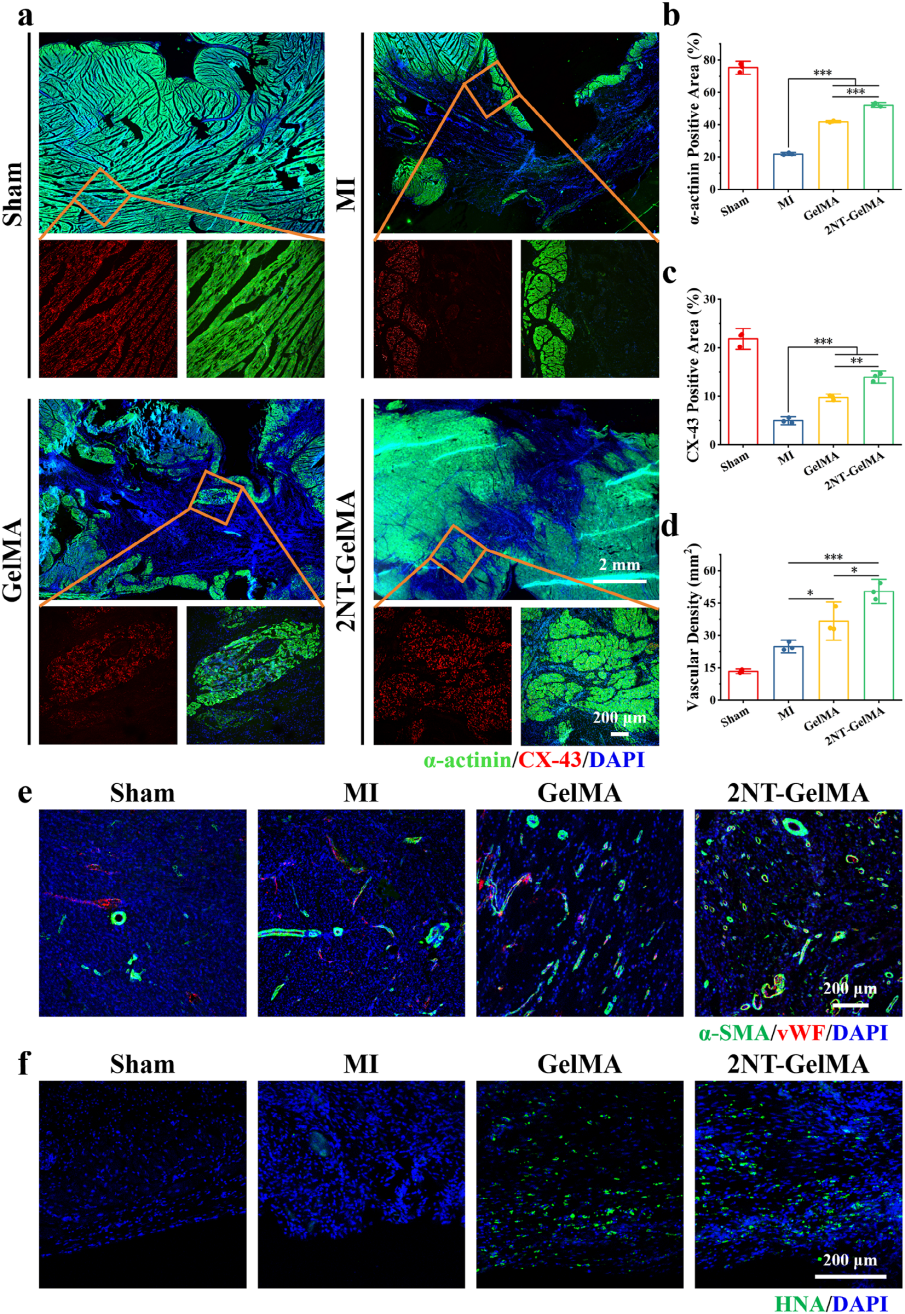
Histological assessment of the infarction regions in minipig hearts after implantation of the 3D bioprinted cardiac patches for 4 weeks. (a) Immunofluorescent staining images of CX‐43 and α‐actinin in the infarction regions of minipig heart sections; (b) statistical results of α‐actinin area in the infarction regions of heart sections (*n* = 3); (c) statistical results of CX‐43 area in the infarction regions of heart sections (*n* = 3); (d) statistical results of vascular density in the infarction regions of heart sections according to the immunofluorescent staining images of vWF and α‐SMA (*n* = 3); (e) immunofluorescent staining images of vWF and α‐SMA in the infarction regions of heart sections (red: vWF; green: α‐SMA; blue: DAPI); and (f) immunofluorescent staining images of human nuclear antigen (HNA) indicating the integration of the exogenous cells in the patches with the host tissues. *p < 0.05, **p < 0.01, or ***p < 0.001. The 3D bioprinted cardiac patches based on NT‐morphology MS significantly accelerated the repair of functionalized myocardial tissues in minipigs.

Subsequently, the myocardium repair effects of the minipig heart were further assessed by immunostaining of the infarction region. As shown in Figure 9a, more α‐actinin‐positive myocardium could be observed in the 2NT‐GelMA group, whereas more fibrous tissue existed in MI and GelMA groups. Meanwhile, more CX‐43 proteins appeared in the infarction regions of the 2NT‐GelMA group, indicating the functional integration among CMs within the tissue [58]. Compared with the MI group, two patches‐implantation groups showed higher α‐actinin and CX‐43 expression in the infarction area, with the 2NT‐GelMA group showing the greatest coverage of α‐actinin and CX‐43‐positive areas (Figures 9b–c). The revascularization effects were also evaluated by immunostaining for vWF and α‐SMA. As shown in Figures 9d–e, more vessels, which were indicated by the co‐localization of vWF and α‐SMA, were formed in the 2NT‐GelMA group. This suggested that in the repair process, 2NT‐GelMA patches activated angiogenesis, which is quite important for the transportation of oxygen and nutrition in cardiac recovery [59].

In order to explore the integration of the patch with the host tissue and the fate of the cells loaded by the patches, the specific expression of human nuclear antigen (HNA) was detected in the infarction region. The presence of HNA‐positive cells demonstrated the stability of the 3D bioprinted cardiac patches after implantation, and some of the hCMs within the patches migrated into the host tissue during the repair process (Figure 9f). This result indicated that the 3D bioprinted cardiac patches integrated well with the host after implantation and the loaded hCMs played a role in the heart repair. In addition, the in vivo safety of implanting the 3D bioprinted cardiac patches was assessed by H and E staining of liver, spleen, lung, and kidney and by blood biochemistry. For one thing, there is no obvious pathological change in the liver, spleen lung and kidney of the patch‐implantation group compared with the Sham group according to the H and E staining images (Figure S22a). For another, the results of blood biochemistry showed that the liver functions and kidney functions in the patch implantation group had no significant difference compared with those in the Sham group (Figure S22b). These results indicated that the implantation of 3D bioprinted cardiac patches showed high safety towards vital organs. While the present study demonstrated promising short‐term repair potential of the 2NT‐GelMA cardiac patch, we recognize that the 4 week experimental period limits the understanding of its long‐term effects post‐implantation. Key aspects such as long‐term tissue remodeling, functional recovery, and the complete degradation process of the patch require further investigation. The long‐term degradation kinetics of MS, along with the in vivo distribution and clearance pathways of its degradation products, are crucial for evaluating its clinical translation potential. In vitro experiments have shown that MS can be endocytosed by cells and exhibits enrichment in mitochondria, suggesting a potential intracellular clearance pathway. Nevertheless, the long‐term fate of MS nanoparticles, particularly NT nanoparticles, needs further investigation in future studies. Therefore, conducting longer‐term animal studies is essential to evaluate the clinical translation potential of this approach. Moreover, due to the high experimental costs associated with animal models (minipigs) and in accordance with the “3R” principles (replacement, reduction, refinement) of animal ethics, the sample size per group was small (*n* = 3). This limited sample size means that the study has limitations in statistical power, increasing the risk of false‐negative results. However, the primary aim of this experiment was to investigate the trend and provide preliminary proof‐of‐concept, while larger‐scale follow‐up studies are essential to draw definitive conclusions.

Taken together, the NT‐containing 3D bioprinted cardiac patches could be safely integrated with the porcine host and significantly promote the repair of myocardial tissue after MI, demonstrating their potential to be used in human myocardial repair and MI treatment.

## 3 Conclusion

In this study, bioenergy‐activating bioinks based on morphology‐modulated MS nanoparticles were developed and the corresponding cardiac patches were 3D bioprinted for infarcted myocardium repair. Firstly, the incorporation of MS with appropriate concentrations enhanced ATP production and contractile function of CMs, which could be attributed to the magnesium and silicon ions released by MS. More importantly, the morphology of MS significantly mediated myocardial energy metabolism and physiological functions. The morphology of MS nanoparticles affected their accumulation in the mitochondria after being taken up by CMs, thereby regulating ATP production capacity. The morphology adjusted the dynamic stiffness of the bioink, thereby indirectly affecting energy metabolism. In summary, the composition and morphology of MS systematically mediated myocardial ATP production of the 3D bioprinted cardiac patches, thus regulating the in vitro physiological activity and in vivo repair function. Finally, 3D bioprinted cardiac patches with MS nanotubes significantly promoted myocardial repair and cardiac function recovery in minipigs. Overall, the bioinks based on morphology‐adjustable MS nanoparticles offer a strategy for promoting myocardium repair from the point of view of bioenergy‐activation and will shed light on bioenergy‐activating inorganic biomaterials design for complex tissue regeneration.

## 4 Materials and Methods

### 4.1 Materials

Magnesium nitrate hexahydrate (Mg(NO_3_)_2_·6H_2_O), sodium metasilicate nonahydrate (Na_2_SiO_3_·9H_2_O), ammonium hydroxide (NH_3_·H_2_O), magnesium chloride hexahydrate (MgCl_2_·6H_2_O), sodium hydroxide (NaOH), tetraethyl orthosilicate (TEOS), ammonium chloride (NH_4_Cl) and ethanol were purchased from Sinopharm Chemical Reagent Co., Ltd. Lithium phenyl (2, 4, 6‐trimethylbenzoyl) phosphinate (LAP), and gelatin were purchased from Sigma‐Aldrich (USA). Methacrylic anhydride was purchased from Shanghai Titan Scientific Co., Ltd.

### 4.2 Synthesis of Magnesium Silicate (MS) Nanoparticles With Different Morphologies

Three MS nanoparticles with different morphologies were synthesized by a hydrothermal method under different conditions.

For irregular particles (IR), 2.1 g Mg(NO_3_)_2_·6H_2_O was added to the solution of ethanol (60 mL) and deionized water (30 mL), 2.3 g Na_2_SiO_3_·9H_2_O was dissolved in deionized water (10 mL). After being stirred for 1 h, the Na_2_SiO_3_ solution was added into the Mg(NO_3_)_2_ solution, and the liquid was stirred for 10 min. Then, the liquid was added into Teflon‐lined hydrothermal reactors (50 mL) and reacted for 24 h at 170°C. Then, the precipitate was harvested and washed using deionized water followed by ethanol. Finally, the IR particles were dried at 50°C for 24 h and saved at 4°C.

For Nanospheres (NS), the silica nanospheres were synthesized first. 64 g of ethanol and 24 g ammonium hydroxide were mixed and stirred at 30°C for 10 min. Then, TEOS (4.2 mL) was added to the liquid and stirred for 1 h. Then, the liquid underwent centrifugation at 5000 rpm for 5 min to get the white precipitates. After being washed using deionized water and ethanol, the SiO_2_ particles were dried at 50°C and saved at 4°C. Then, the NS were synthesized using the following protocol. 0.2 g SiO_2_ particles were added to deionized water (40 mL) and then ultrasonically dispersed for 30 min. The SiO_2_ solution was named Liquid 1. Then, 0.3 g MgCl_2_·6H_2_O and 1.08 g of NH_4_Cl were added to deionized water (60 mL). Then, NH_3_·H_2_O (2 mL) was added slowly to the liquid and stirred for 30 min. The liquid was named Liquid 2. Then, Liquid 1 and ethanol (100 mL) were added quickly to Liquid 2 and stirred for 1 h. Then, the liquid was added to a Teflon‐lined hydrothermal reactors (50 mL) and reacted for 12 h at 140°C. After that, the precipitate was harvested and washed using deionized water followed by ethanol. Finally, the NS particles were dried at 50°C and saved at 4°C.

For NT, 0.28 g Na_2_SiO_3_·9H_2_O and 0.26 g Mg(NO_3_)_2_·6H_2_O were added in deionized water (2 mL) in two containers separately. Then, the Mg(NO_3_)_2_ solution and Na_2_SiO_3_ solution were added to a solution of ethanol (50 mL) and ethylene glycol (10 mL) and stirred for 10 min. Then, 1.00 g NaOH was added to the liquid and stirred for 1 h. Then, the liquid was added to Teflon‐lined hydrothermal reactors (50 mL) and reacted for 24 h at 210°C. Then, the precipitate was harvested and washed using deionized water followed by ethanol. Finally, the NT particles were dried at 50°C and saved at 4°C.

### 4.3 Characterization of Magnesium Silicate (MS) Nanoparticles With Different Morphologies

The morphologies of MS nanoparticles were observed using a scanning electron microscope (SEM, SU‐8200, Hitachi, Japan). The powder samples were dispersed in ethanol by ultrasonicating for 10 min, dropped onto aluminum foil, and allowed to dry before observation. The SEM images were acquired using the secondary electron (SE) imaging mode. The accelerating voltages applied for the different morphologies were optimized as follows: 10 kV for the IR, 5 kV for the NS, and 15 kV for the NT. Microstructure and elemental distribution of MS nanoparticles were studied using a transmission electron microscope (TEM, Tecnai G2 F20, FEI Electron Optics, Netherlands). The powder samples were ultrasonically dispersed in ethanol for 10 min, dropped onto carbon‐coated copper grids, and dried prior to observation at an accelerating voltage of 200 kV. The phases of MS nanoparticles were studied using XRD, Geigerflex, Rigaku Co., Japan. The XRD measurement was performed using a Cu Kα radiation source over a 2θ range of 10° to 80°, with a step size of 0.02° and a scan speed of 0.6 s per step.

### 4.4 Cell Culture

rCMs were isolated referring to a prior study [60]. Firstly, the newborn Sprague‐Dawley (SD) rats (1–3‐day‐old) were purchased from the Shanghai Experimental Animal Center. Then, the hearts of SD rats were harvested quickly and washed with phosphate buffered solution (PBS, E607008, Sangon Biotech, China) three times. Then, the hearts were treated with trypsin for 12 h at 4°C. Then, the hearts were digested with 0.1% collagenase type II (Sigma‐Aldrich, USA) and filtered out the dispersive cells using a cell strainer (70 µm). Finally, rCMs were isolated by differential adhesion for 2 h. All nonadherent cells were harvested for 3D bioprinting. rCMs were cultured in Dulbecco's modified Eagle medium (DMEM, 11965092, Gibco, USA) supplemented with 1% penicillin‐streptomycin (P/S, Gibco, USA), and 15% fetal bovine serum (FBS, Gibco, USA).

Human CMs (hCMs) were differentiated from human‐induced pluripotent stem cells (hiPSCs) via a cardiomyocyte differentiation kit. Both the hiPSCs and the kit were purchased from Nanjing Cosmos Biotechnology Co., Ltd. After following the differentiation steps of the kit for 10 days, beating hCMs were harvested for 3D bioprinting. All cells were cultured in an incubator (37°C, 5% CO_2_).

### 4.5 Preparation and Characterization of MS‐GelMA Bioinks

MS‐GelMA bioinks were prepared from GelMA and MS nanoparticles with different morphologies. GelMA was synthesized referring to a prior study [61]. Firstly, 0.6 g GelMA and 0.025 g LAP were dissolved in deionized water (5 mL) at 60°C for 20 min and then sterilized via a 0.22 µm filter. Secondly, MS nanoparticles (0 g, 0.012 g, 0.024 g, 0.036 g) were sterilized by ultraviolet and added to the PBS solution (5 mL). Thirdly, MS nanoparticles dispersed in PBS were added to the GelMA solution in a 1:1 volume ratio. Based on the mass proportion of GelMA and MS nanoparticles (0%, 2%, 4%, 6%), the inks were named GelMA, 2NS (2IR, 2NT)‐GelMA, 4NS (4IR, 4NT)‐GelMA and 6NS‐GelMA, respectively.

Microstructural and elemental compositions of the bioinks were studied by SEM. Rheological properties of the bioinks were assessed by a rheometer (MCR301, Anton‐Paar, Austria). The G′ and G″ of the bioinks were tested a frequency 1 Hz at 16°C. Shear‐thinning properties of the bioinks were studied with a shear rate range of 0.1–100 s^−1^ at 16°C.

In order to assess the Mg and Si ions release properties of the bioinks, 3D printed patches (height: 0.7 mm, distance of filaments: 1.2 mm, diameter: 8 mm) were soaked in 1 mL tris‐HCl (ST774, Beyotime, China) and incubated in an incubator. The Tris‐HCl was harvested and filtered via a 0.22 µm filter at 1, 4 and 7 days. The concentration of Mg and Si ions in the tris‐HCl was tested by inductively coupled plasma atomic emission spectroscopy (ICP‐AES, Varian 715ES, Palo Alto, USA). The operating parameters were set as follows: a radio frequency power of 1.1 kW, with a plasma gas flow rate of 15 L/min and a nebulizer pressure of 200 kPa.

### 4.6 3D Bioprinting of Cardiac Patches

For the printing of cardiac patches, a cell suspension (200 µL) containing 3 × 10^7^ rCMs/mL or hCMs/mL was incorporated into the bioinks. The cardiac patches with different morphologies of MS nanoparticles were obtained by a machine of 3D printing (Bioscaffolder 3.2, GeSim, Germany). Specifically, the bioinks were added into a stainless steel cartridge fitted with a 27G needle (250 µm inner diameter) and cooled for 15 min at 4°C. Extrusion pressures were adjusted to a range from 20 to 60 kPa, with cartridge temperatures maintained at 16°C. The platform for deposition was cooled to 4°C. After being printed, the cardiac patches were cross linked for 20 s by blue light (EFL‐LS‐1601, EFL, China). Finally, the cardiac patches were cultured in an incubator (37°C, 5% CO_2_).

For the in vitro experiments, the height of the patches was 0.7 mm, the distance of filaments was 1.2 mm, and the diameter of the patches was 8 mm. For the animal experiments in rats, the height of the patches was 1 mm, the distance of filaments was 1.2 mm, and the diameter of the patches was 16 mm. For the animal experiments in minipigs, the height of the patches was 1 mm, the distance of filaments was 1.2 mm, and the diameter of the patches was 30 mm.

### 4.7 Rheological Characterization of the Cardiac Patches

In order to evaluate the change of the G′ of the patches during culture, the 3D bioprinted patches (height: 1 mm, distance of filaments: 1.2 mm, diameter: 16 mm) were soaked in a 12‐well plate with 2 mL culture medium, and incubated in an incubator (37°C, 5% CO_2_). The G′ of the patches was tested using a rheometer after being incubated for 1, 4, and 7 days.

### 4.8 Computational Simulation

The calculations utilizing spin‐polarized density functional theory (DFT) were executed with the Vienna Ab initio simulation package (VASP), employing the generalized gradient approximation of Perdew‐Burke‐Ernzerhof for modeling electron exchange and correlation [62]. A plane‐wave basis set with a cutoff energy of 350 eV was applied. The projector‐augmented wave (PAW) method was utilized to depict [63]. For the geometric optimization, a (3 × 3 × 1) k‐point mesh was implemented, with convergence criteria set to 10^−4^ eV for energy and 0.05 eV/Å for force. The models of GelMA, IR‐GelMA, NS‐GelMA, and NT‐GelMA were first chosen for simulating the elastic modulus of different morphologies of MS on GelMA molecular fragments.

### 4.9 Cell Viability Assay

Cell viability in 3D bioprinted cardiac patches was assessed by the live/dead assay using the Calcein‐AM/PI kit (Dojindo, Japan). Firstly, the working solution was concocted following the volume ratio as culture medium: PI: AM = 1000: 3: 2. Then, the cardiac patches were incubated with the working solution for 30 min in an incubator. After that, the images of live and dead cells in the cardiac patches were recorded using a fluorescence microscope (DMi8 S, Leica, Germany). Finally, the ImageJ software was used to count the numbers of live and dead cells.

### 4.10 Calcium Transient Assay

The calcium transient in rCMs of the cardiac patches was assessed by a calcium assay kit (F14201, Thermo Fisher Scientific, USA). After being cultured for 4 and 7 days, the cardiac patches were stained with the kit according to the instruction manual. Then, the calcium transient images and videos were recorded using a fluorescence microscope. Finally, the ImageJ software was used to measure the peak numbers per 10 s and the time to peak of the calcium transient signals in the cardiac patches.

### 4.11 Immunofluorescent Protein Staining Assay

The relative protein expression levels in the cardiac patches were assessed by an immunofluorescence staining assay. Firstly, the cardiac patches were fixed in 4% paraformaldehyde for 1 h after being cultured for 4 and 7 days. Subsequently, they were treated with 0.1% Triton X‐100 (Sigma Aldrich, USA) for 5 min. Then, the cardiac patches were treated with a 5% BSA solution to avoid nonspecific staining for 1 h. Then, the cardiac patches were treated with a primary antibody for 12 h at 4°C and washed with PBS. Finally, the cardiac patches were treated with a secondary antibody for 1 h at room temperature and then with DAPI for nuclear staining for 10 min.

The images were recorded by a confocal laser scanning microscope (CLSM, TCS SP8, Leica, Germany), and the protein expressions were semi‐quantified by the ImageJ software. The relevant primary antibodies were as below: α‐actinin (1:500, ab9465, Abcam, USA), CX‐43 (1:500, ab11370, Abcam, USA), and vinculin (1:500, ab129002, Abcam, USA). The secondary antibodies were as follows: Alexa Fluor 488 Goat Anti‐Mouse IgG (H and L) (1:1000, ab150113, Abcam, USA) and Alexa Fluor 647 Goat Anti‐Rabbit IgG (H&L) (1:1000, ab150079, Abcam, USA).

### 4.12 Relative Gene Expressions of rCMs in the Cardiac Patches

In order to assess the function of rCMs in the cardiac patches at the gene level, the RT‐qPCR assay was performed. Firstly, the cardiac patches were treated with GelMA Lysis Buffer (EFL, EFL‐GM‐LS‐001, China) for 2 h after being cultured for 7 days and the cells were harvested. Secondly, the RNA of the rCMs in the cardiac patches was extracted via TRIzol reagent (Invitrogen, USA), and the complementary DNA (cDNA) was obtained using a PrimeScript first Strand cDNA synthesis kit (TOYOBO, Japan). The housekeeping gene was GAPDH, and the gene expression levels were calculated using the 2^−ΔΔCT^ approach. The primers′ sequences were provided in Table S1.

### 4.13 Enzyme‐Linked Immunosorbent Assay (ELISA)

After 7 days of culture, the cells were harvested from cardiac patches under a 2 h treatment with GelMA Lysis Buffer.The total proteins were extracted by a RIPA lysis buffer, and the concentration of α‐actinin, CX‐43, p‐AMPK and PGC‐1α was measured using an ELISA Kit (XY9R1777 for α‐actinin, XY9R0881 for CX‐43, XY9R785631 for p‐AMPK and XY9R09901 for PGC1‐α, X‐Y Biotechnology, China). Absorbance at 450 nm was tested by microplate reader (Epoch, BIO‐TEK, USA).

### 4.14 Inner‐Cellular Distribution of the MS Nanoparticles

The uptake of MS nanoparticles by cells in the cardiac patches was observed by TEM (HIT7800, Hitachi, Japan). Firstly, the cardiac patches were fixed in glutaraldehyde fixative (BL911A, Biosharp, China) for 1 h after being cultured for 7 days. Then, the cardiac patches were treated with GelMA Lysis Buffer for 2 h. Then, the cells were harvested and fixed in glutaraldehyde fixative again. Then, the samples for TEM were prepared by Shiyanjia Lab and observed by TEM.

In order to assess the co‐localization of mitochondria and the MS nanoparticles in the cardiac patches, MS nanoparticles were labeled with fluorescence (FITC, F8070, Solarbio, China) according to a prior study. Specifically, 0.4 g NaOH and 4 mg FITC were added to a solution of 0.5 mL (3‐aminopropyl) triethoxysilane (APTES, Thermo, USA) and 5 mL ethanol. The liquid was kept in the dark and stirred for 24 h. Then, 50 mg of MS nanoparticles were added to the liquid and stirred for 12 h. Then, the mixture was centrifuged at 10,000 rpm for 15 min to obtain the precipitate and was washed using deionized water followed by ethanol. After that, the MS‐FITC nanoparticles were dried at 50°C for 24 h and saved at 4°C. Subsequently, the MS‐FITC nanoparticles were added to the bioinks and the prepared the 2IR‐GelMA, 2NS‐GelMA and 2NT‐GelMA cardiac patches. After being cultured for 7 days, the cardiac patches were stained with a Mito‐Tracker kit (C1034, Beyotime, China). Finally, the images were obtained using CLSM, and the Pearson's correlation coefficient for the co‐localization of mitochondria and MS nanoparticles was measured using ImageJ software.

### 4.15 Adenosine Triphosphate (ATP) Content Detection

ATP production levels of the 3D bioprinted cardiac patches were assessed by an ATP content assay kit (BL852B, Biosharp, China). The cardiac patches were treated with GelMA Lysis Buffer for 2 h and the cells were harvested to assess the ATP content by the kit. The absorbance values A0 (0 min) and A1 (15 min) at 340 nm were tested by a microplate reader (Epoch, BIO‐TEK, USA). Relative ATP production = (A1–A0)/(A1_GelMA_—A0_GelMA_).

### 4.16 Mitochondrial Membrane Potential Assessment

To evaluate mitochondrial function under harsh conditions, cardiac patches were treated with 4 h of hypoxic and low‐glucose conditions after 7 days of culture, following previous reports [64]. Mitochondrial membrane potential was assessed using the Enhanced Mitochondrial Membrane Potential Assay Kit with JC‐1 (C2003S, Beyotime, China) for 2 h at 37°C. J‐monomer (green) and J‐aggregate (red) fluorescence were acquired by CLSM, and the mean fluorescence intensity ratio (red/green) was quantified with ImageJ software to determine the mitochondrial membrane potential.

### 4.17 RNA Sequencing

rCMs were harvested from the 3D bioprinted cardiac patches after being cultured for 7 days. The RNA was extracted according to the PCR protocol. Then, the RNA sequencing was performed by Shanghai Biotechnology Corporation.

### 4.18 MI Rat Model and Cardiac Patches Implantation

Male SD rats (225–250 g) were used for establishing MI model and categorized into 6 groups: Sham (*n* = 6), MI (*n* = 6), GelMA (*n* = 6), 2IR‐GelMA (*n* = 6), 2NS‐GelMA (*n* = 6), and 2NT‐GelMA (*n* = 6). The MI model was induced by ligating the LAD according to a prior study [57]. All cardiac patches (diameter: 16 mm, height: 1 mm) were cultured for 7 days before being implanted. For the Sham group, the rats underwent only thoracotomy, while the other rats underwent ligation operations of the LAD after thoracotomy. For the GelMA, 2IR‐GelMA, 2NS‐GelMA, and 2NT‐GelMA groups, the cardiac patches were implanted on the infarcted myocardium after ligation for 15 min. All patches were secured to the heart with fibrin glue according to a prior study [65]. The fibrin glue has been widely used for cardiac patch implantation in MI models due to its excellent biocompatibility and adhesive properties. As shown in Supplementary Video 9, the patch was secured onto the heart surface with 20 µL fibrinogen (F36700, Acmec, China, 110.5 mg/mL in deionized water) and 20 µL thrombin (T832140, Macklin, China, 500 IU/mL in deionized water with 0.04 mmol/mL CaCl2). The patch remained securely attached to the heart under hydrodynamic flow.

### 4.19 Degradation Behavior of GelMA, 2IR‐GelMA, 2NS‐GelMA, and 2NT‐GelMA Hydrogels

GelMA, 2IR‐GelMA, 2NS‐GelMA, and 2NT‐GelMA hydrogels (1 cm in height, *n* = 3) were prepared and placed in a 24‐well plate. Each well received 2 mL of tris‐HCl buffer (pH 5.5/8.5, or pH 7.4 containing 0.01% Type II collagenase). Following incubation at 37°C, the initial mass (*M*_0_) was recorded. At designated time points, samples were collected and lyophilized to determine the dry mass (*M*). The remaining hydrogel percentage was calculated as (*M*/*M*_0_) × 100%.

### 4.20 Echocardiography Evaluation of Rat Cardiac Function

The cardiac function of rats was assessed by an IE33 echocardiography machine (Vevo2100, Visual Sonics, Canada) after 1 and 4 weeks post‐surgery. M‐mode images were captured via a 40 MHz transducer, and relative parameters were measured in short‐axis views. The parameters included left ventricle fraction shorting (FS), left ventricle ejection fraction EF, LVIDd, and LVIDs.

### 4.21 Histological and Immunofluorescence Assay of Tissue Sections of Rats

After 4 weeks post‐surgery, the rats were euthanized, and the whole hearts were collected and sectioned into three sections. Then, the transverse sections of the hearts were recorded. Subsequently, the slices were frozen in optimal cutting temperature (OCT) compound for 48 h at −20°C. Finally, the tissues were sectioned at a thickness of 10 µm by a freezing microtome machine (CryoStar NX70, Thermo, USA).

The infarct area and wall thickness were assessed by a Masson's trichrome staining kit (G1340, Solarbio, China). The slices were fixed in 4% paraformaldehyde for 20 min and washed with PBS. Then, the tissue sections were stained using the kit. The images were recorded by an optical microscope (Zeiss, Germany) and subsequently analyzed using ImageJ software. The assessment of the infarct area was determined by calculating the ratio of the internal boundary length of the scar to the total internal boundary length of the left ventricle. The wall thicknesses were assessed three times, and the values were then averaged.

For immunofluorescence staining, the slices were fixed in 4% paraformaldehyde for 20 min and washed with PBS. Then, the slices were treated with proteinase K solution (P78893, Abcone, China) at 37°C for 15 min. Then, the slices were treated with 0.3% Triton X‐100 and 10% donkey serum (BL939A, Biosharp, China) for 1 h at room temperature. Then, the slices were treated with a primary antibody for 12 h at 4°C, and secondary antibody for 1 h at room temperature. Finally, the slices were treated with DAPI for 10 min and covered with a fluorescent mounting medium (S3023, Dako, Denmark).

The images were recorded by CLSM and analyzed by ImageJ software. The relevant primary antibodies were as below: α‐actinin (1:500, ab9465, Abcam, USA), CX‐43 (1:500, ab11370, Abcam, USA), WGA (4 µg/mL, L4895, Sigma‐Aldrich, USA), vWF (1:500, ab6994, Abcam, USA), and α‐SMA (1:500, ab7817, Abcam, USA). The secondary antibodies were as follows: Alexa Fluor 488 Goat Anti‐Mouse IgG (H and L) (1:1000, ab150113, Abcam, USA) and Alexa Fluor 647 Goat Anti‐Rabbit IgG (H and L) (1:1000, ab150079, Abcam, USA).

### 4.22 MI Minipig Model and Cardiac Patches Implantation

Male minipigs (1 year, 25–30 kg) were used to establish the MI model and categorized into four groups: Sham (*n* = 3), MI (*n* = 3), GelMA (*n* = 3) and 2NT‐GelMA (*n* = 3). The minipigs were anesthetized with isoflurane (1–3%) and propofol (10 mL/h) and then subjected to thoracotomy. The MI model in minipigs was conducted according to a prior study [55]. Specifically, the MI model was induced by ligating the LAD for 10 min with a 5–0 polypropylene suture (Prolene, Ethicon) and reperfused twice before permanently tied. MI models were verified by ischemic pale discoloration of the myocardial surface and ST‐segment elevation on the electrocardiogram (ECG). All cardiac patches (diameter: 30 mm, height: 1 mm, three patches per heart) were cultured for 7 days before being implanted. For the Sham group, the minipigs underwent only thoracotomy. For the GelMA and 2NT‐GelMA groups, the cardiac patches were implanted on the infarcted myocardium by being secured to the infarcted myocardium with fibrin glue [65].

### 4.23 Echocardiography of Minipigs

Echocardiography of minipigs was assessed by an echocardiograph (M90 SCI, Mindray Animal, China) before operation, and at 2 and 4 weeks after occlusion. The minipigs were anesthetized, and then dynamic images of the left ventricle were recorded. The relative parameters (EF, FS, LVIDs, and LVIDd) were measured from M‐mode tracings.

### 4.24 Electrical Signal Propagation of Ex Vivo Minipig Heart

The electrical signal propagation of the ex vivo minipig heart was measured according to a prior study [55]. After the echocardiographic test was completed at 4 weeks post‐surgery, the minipigs were euthanized. The whole hearts were harvested quickly and soaked in KH solution (PB180348, Pricella, China). The electrical signal propagation of ex vivo minipig heart was assessed using a signal acquisition system (BL‐420, Tech Man, China). The stimulating electrode was connected to the tissue at the end of the infarcted area, and two‐lead method ECG electrodes were used for signal detection. The output pulse was set to 2 Hz.

### 4.25 Histological and Immunofluorescence Assay of Tissue Sections of Minipigs

The lung, liver, spleen, and kidney were fixed in 4% paraformaldehyde for 72 h, then encased in paraffin, sectioned into 10 µm slices and stained with Hematoxylin and Eosin (H and E) staining kit (C0105S, Beyotime, China) for pathological examination. The hearts were sliced into 4 slices (thickness of 1 cm for each slice) from the apex to the atrium, and the gross morphological images of the slices were recorded. Next, the heart slices were dehydrated with 15% sucrose solution for 24 h and 30% sucrose solution for 24 h, then encased in OCT compound and sectioned into 10 µm slices. Then the sections were stained with Masson's trichrome staining kit to assess the infarct area. Moreover, the immunofluorescence staining of specific proteins in the sections was conducted by using the same method as described above.

The relevant primary antibodies were as below: α‐actinin (1:500, ab9465, Abcam, USA), CX‐43 (1:500, ab11370, Abcam, USA), HNA (1:500, ab191181, Abcam, USA), vWF (1:500, ab6994, Abcam, USA), α‐SMA (1:500, ab7817, Abcam, USA). The secondary antibodies were as follows: Alexa Fluor 488 Goat Anti‐Mouse IgG (H and L) (1:1000, ab150113, Abcam, USA) and Alexa Fluor 647 Goat Anti‐Rabbit IgG (H and L) (1:1000, ab150079, Abcam, USA).

### 4.26 Collection and Testing of Blood Samples

In order to assess the potential toxicity of implanted cardiac patches, blood specimens were obtained before occlusion and at 2 and 4 weeks after occlusion. Liver and kidney functions were assessed by measuring the level of serum creatinine (Cr), blood urea nitrogen (BUN), serum aspartate aminotransferase (AST), and alanine transaminase (ALT) in the blood of minipigs. All blood specimens were analyzed by Guangzhou Huayin Medical Laboratory Center Co., Ltd.

### 4.27 Statistical Analysis

All data were expressed as mean ± standard deviation and analyzed in Origin 2022 software (OriginLab, USA). One‐way analysis of variance (ANOVA) was utilized to conduct multiple group comparisons. Significant difference was considered when *p < 0.05, **p < 0.01, or ***p < 0.001.

## Author Contributions

**Zhibin Liao**: conceptualization, data curation, investigation, formal analysis, methodology, writing – original draft, writing – review and editing. **Chen Qin**: conceptualization, data curation, investigation, formal analysis, methodology, writing – original draft, writing – review and editing. **Erhong Song**: formal analysis, methodology, writing – original draft. **Chengbin Ding**: formal analysis, methodology. **Zhixu Wang**: formal analysis, methodology. **Yan Sun**: methodology. **Chen Song**: methodology. **Junjie Liu**: methodology. **Jingge Ma**: investigation, methodology. **Hongjian Zhang**: methodology. **Leyu Wang**: conceptualization, resources, funding acquisition, writing – review and editing, supervision. **Chengtie Wu**: conceptualization, resources, funding acquisition, writing – review & editing, supervision. all authors discussed the results and revised the manuscript.

## Conflicts of Interest

The authors declare no conflicts of interest.

## Ethics Statement

The rat animal experiments were approved by the Southern Medica University Animal Ethics Committee (NO. SMUL202311025). The minipig animal experiments were approved by the Animal Ethics Committee (Longgui Xingke, Guangzhou, NO. XK20240228001).

## Supporting information




**Supporting File 1**: exp270161‐sup‐0001‐SuppMat.docx.

**Supporting File 2**: exp270161‐sup‐0002‐Supp‐Data1.docx.

**Supporting File 3**: exp270161‐sup‐0003‐Supp‐Data2.docx.

**Supporting File 4**: exp270161‐sup‐0004‐Video1.mov.

**Supporting File 5**: exp270161‐sup‐0005‐Video2.mov.

**Supporting File 6**: exp270161‐sup‐0006‐Video3.mov.

**Supporting File 7**: exp270161‐sup‐0007‐Video4.mov.

**Supporting File 8**: exp270161‐sup‐0008‐Video5.mov.

**Supporting File 9**: exp270161‐sup‐0009‐Video6.mov.

**Supporting File 10**: exp270161‐sup‐0010‐Video7.mov.

**Supporting File 11**: exp270161‐sup‐0011‐Video8.mov.

**Supporting File 12**: exp270161‐sup‐0012‐Video9.mov.

## Data Availability

All data needed to evaluate the conclusions in the paper are present in the paper and/or the Supplementary Information. Additional data related to this paper may be requested from the authors.

## References

[1] N. D. Wong , “Epidemiological Studies of CHD and the Evolution of Preventive Cardiology,” Nature Reviews Cardiology 11 (2014): 276–289, 10.1038/nrcardio.2014.26. 24663092

[2] R. Song , C. Dasgupta , C. Mulder , and L. Zhang , “MicroRNA‐210 Controls Mitochondrial Metabolism and Protects Heart Function in Myocardial Infarction,” Circulation 145, no, 15 (2022): 1140–1153, 10.1161/CIRCULATIONAHA.121.056929. 35296158 PMC9007902

[3] G. D. Lopaschuk , Q. G. Karwi , R. Tian , A. R. Wende , and E. D. Abel , “Cardiac Energy Metabolism in Heart Failure,” Circulation Research 128, no. 10 (2021): 1487–1513, 10.1161/CIRCRESAHA.121.318241. 33983836 PMC8136750

[4] X. Li , F. Wu , S. Guenther , et al., “Inhibition of Fatty Acid Oxidation Enables Heart Regeneration in Adult Mice,” Nature 622 (2023): 619–626, 10.1038/s41586-023-06585-5. 37758950 PMC10584682

[5] X. Sun , H. Chen , R. Gao , et al., “Intravenous Transplantation of an Ischemic‐Specific Peptide‐TPP‐Mitochondrial Compound Alleviates Myocardial Ischemic Reperfusion Injury,” Acs Nano 17, no. 2 (2023): 896–909, 10.1021/acsnano.2c05286. 36625783 PMC9878726

[6] X. Zhang , Y. Sun , R. Yang , et al., “An Injectable Mitochondria‐Targeted Nanodrug Loaded‐Hydrogel for Restoring Mitochondrial Function and Hierarchically Attenuating Oxidative Stress to Reduce Myocardial Ischemia‐Reperfusion Injury,” Biomaterials 287 (2022): 121656. 35792386 10.1016/j.biomaterials.2022.121656

[7] G. Gao , J. Li , Y. Ma , et al., “Dual‐Responsive Multi‐Functional Silica Nanoparticles with Repaired Mitochondrial Functions for Efficient Alleviation of Spinal Cord Injury,” Exploration (Beijing, China) 5 (2025): 270012–270012. 40585767 10.1002/EXP.70012 PMC12199316

[8] M. Sun , W. Jiang , N. Mu , Z. Zhang , L. Yu , and H. Ma , “Mitochondrial Transplantation as a Novel Therapeutic Strategy for Cardiovascular Diseases,” Journal of Translational Medicine 21 (2023): 347. 37231493 10.1186/s12967-023-04203-6 PMC10210445

[9] P. Li , J. Ge , and H. Li , “Lysine acetyltransferases and lysine deacetylases as targets for cardiovascular disease,” Nature Reviews Cardiology 17 (2020): 96–115, 10.1038/s41569-019-0235-9. 31350538

[10] H. Liu , Y. Du , J.‐P. St‐Pierre , et al., “Bioenergetic‐Active Materials Enhance Tissue Regeneration by Modulating Cellular Metabolic state,” Science Advances 6, no. 13 (2020): eaay7608. 32232154 10.1126/sciadv.aay7608 PMC7096169

[11] X. Hong , G. Tian , B. Dai , et al., “Copper‐Loaded Milk‐Protein Derived Microgel Preserves Cardiac Metabolic Homeostasis After Myocardial Infarction,” Advanced science 11, no. 35 (2024): e2401527, 10.1002/advs.202401527. 39007192 PMC11425262

[12] J. Na , Z. Yang , Q. Shi , et al., “Extracellular Matrix Stiffness as an Energy Metabolism Regulator Drives Osteogenic Differentiation in Mesenchymal Stem Cells,” Bioactive Materials 35 (2024): 549–563, 10.1016/j.bioactmat.2024.02.003. 38434800 PMC10909577

[13] A. V. Singh , M. Raymond , F. Pace , et al., “Astrocytes Increase ATP Exocytosis Mediated Calcium Signaling in Response to Microgroove Structures,” Scientific Reports 5 (2015): 7847. 25597401 10.1038/srep07847 PMC4297955

[14] A. M. Brokesh and A. K. Gaharwar , “Inorganic Biomaterials for Regenerative Medicine,” ACS Applied Materials and Interfaces 12, no. 5 (2020): 5319–5344, 10.1021/acsami.9b17801. 31989815

[15] W. Dang , X. Wang , J. Li , et al., “3D Printing of Mo‐Containing Scaffolds With Activated Anabolic Responses and Bi‐Lineage Bioactivities,” Theranostics 8, no. 16 (2018): 4372–4392, 10.7150/thno.27088. 30214627 PMC6134938

[16] X.‐T. He , X. Li , M. Zhang , et al., “Role of Molybdenum in Material Immunomodulation and Periodontal Wound Healing: Targeting Immunometabolism and Mitochondrial Function for Macrophage Modulation,” Biomaterials 283 (2022): 121439, 10.1016/j.biomaterials.2022.121439. 35247634

[17] R. M. Visalakshan , L. E. G. Garcia , M. R. Benzigar , et al., “The Influence of Nanoparticle Shape on Protein Corona Formation,” Small 16, no. 25 (2020): e2000285. 32406176 10.1002/smll.202000285

[18] W. C. W. Chan , “Principles of Nanoparticle Delivery to Solid Tumors,” BME frontiers 4 (2023): 16, 10.34133/bmef.0016. PMC10085247 37849661

[19] J. S. Park , C. J. Burckhardt , R. Lazcano , et al., “Mechanical Regulation of Glycolysis via Cytoskeleton Architecture,” Nature 578 (2020): 621–626, 10.1038/s41586-020-1998-1. 32051585 PMC7210009

[20] M. Liu , E.‐M. Jeong , H. Liu , et al., “Magnesium Supplementation Improves Diabetic Mitochondrial and Cardiac Diastolic Function,” JCI Insight 4 (2019): e123182. 30626750 10.1172/jci.insight.123182 PMC6485371

[21] Y. Que , J. Shi , Z. Zhang , et al., “Ion Cocktail Therapy For Myocardial Infarction by Synergistic Regulation of Both Structural and Electrical Remodeling,” Exploration 4, no. 3 (2024): 20230067–20230067, 10.1002/EXP.20230067. 38939858 PMC11189571

[22] B. Kong and Y. Zhao , “3D Bioprinting for Biomedical Applications,” BME Frontiers 4 (2023): 10, 10.34133/bmef.0010. PMC10521671 37849677

[23] J. Ma , C. Qin , J. Wu , et al., “3D Multicellular Micropatterning Biomaterials For Hair Regeneration and Vascularization,” Materials Horizons 10, no. 9 (2023): 3773–3784, 10.1039/D3MH00528C. 37409407

[24] L. Luo , Y. Li , Z. Bao , et al., “Pericardial Delivery of SDF‐1 α Puerarin Hydrogel Promotes Heart Repair and Electrical Coupling,” Advanced Materials 36 (2024): 202302686. 10.1002/adma.202302686 37665792

[25] R. Hang , S. Liu , Y. Liu , et al., “Preparation, Characterization, Corrosion Behavior and Cytocompatibility of NiTiO3 Nanosheets Hydrothermally Synthesized on Biomedical NiTi Alloy,” Materials Science and Engineering: C 97 (2019): 715–722, 10.1016/j.msec.2018.12.124. 30678960

[26] G. Jahanmir , M. J. Abdekhodaie , and Y. Chau , “Stochastic Modeling of Degradation Behavior of Hydrogels,” Macromolecules 51, no. 11 (2018): 3941–3952, 10.1021/acs.macromol.8b00165.

[27] D. Hou , J. Xu , Y. Zhang , and G. Sun , “Insights Into the Molecular Structure and Reinforcement Mechanism of the Hydrogel‐Cement Nanocomposite: An Experimental and Molecular Dynamics Study,” Composites Part B‐Engineering 177 (2019): 107429, 10.1016/j.compositesb.2019.107421.

[28] A. Namdar and E. Salahinejad , “Advances in Ion‐Doping of Ca‐Mg Silicate Bioceramics For Bone Tissue Engineering,” Coordination Chemistry Reviews 478 (2023): 215001, 10.1016/j.ccr.2022.215001.

[29] E. Hinde , K. Thammasiraphop , H. T. T. Duong , et al., “Pair Correlation Microscopy Reveals the Role of Nanoparticle Shape in Intracellular Transport and Site of Drug Release,” Nature Nanotechnology 12 (2017): 81–89, 10.1038/nnano.2016.160. 27618255

[30] I. Pilchova , K. Klacanova , Z. Tatarkova , P. Kaplan , and P. Racay , “The Involvement of Mg^2+^ in Regulation of Cellular and Mitochondrial Functions,” Oxidative Medicine and Cellular Longevity 2017 (2017): 6797460, 10.1155/2017/6797460. 28757913 PMC5516748

[31] S. Yu , J. Du , Q. Zhang , Z. Li , S. Ge , and B. Ma , “4D Printed Protein‐AuNR Nanocomposites With Photothermal Shape Recovery,” Advanced Functional Materials 34, no. 14 (2024): 2311209.PMID: 38966003. 38966003 10.1002/adfm.202311209 PMC11221775

[32] Z. Qin , J. Chen , F. Liu , et al., “Jellyfish Stings‐Induced Cardiac Failure Was Ameliorated Through AAG‐mediated Glycogen‐Driven ATP Production,” Exploration (Beijing, China) 5, no. 1 (2025): 20230089–20230089. 40040825 10.1002/EXP.20230089 PMC11875447

[33] X. Han , R. Zheng , J. Zhang , et al., “Cardiomyocyte OTUD1 Drives Diabetic Cardiomyopathy via Directly Deubiquitinating AMPKα2 and Inducing Mitochondrial Dysfunction,” Nature Communications 16 (2025): 6668. 10.1038/s41467-025-61901-z PMC12276362 40683882

[34] U. Flögel , T. Laussmann , A. Gödecke , et al., “Lack of Myoglobin Causes a Switch in Cardiac Substrate Selection,” Circulation Research 96, no. 8 (2005): E68–E75. 15817884 10.1161/01.RES.0000165481.36288.d2

[35] G. M. Ducasa , A. Mitrofanova , S. K. Mallela , et al., “ATP‐Binding Cassette A1 Deficiency Causes Cardiolipin‐Driven Mitochondrial Dysfunction in Podocytes,” Journal of Clinical Investigation 129 (2019): 3387–3400, 10.1172/JCI125316. 31329164 PMC6668702

[36] Q. Zhu , M. E. Combs , J. Liu , et al., “GRAF1 Integrates PINK1‐Parkin Signaling and Actin Dynamics to Mediate Cardiac Mitochondrial Homeostasis,” Nature Communications 14 (2023): 8187. 10.1038/s41467-023-43889-6 PMC10713658 38081847

[37] R. Ma , L. Ma , W. Weng , et al., “DUSP6 SUMOylation Protects Cells from Oxidative Damage via Direct Regulation of Drp1 Dephosphorylation,” Science Advances 6 (2020): eaaz0361. 32232156 10.1126/sciadv.aaz0361 PMC7096176

[38] Y. Mulla , M. J. Avellaneda , A. Roland , et al., “Weak Catch Bonds Make Strong Networks,” Nature Materials 21 (2022): 1019–1023, 10.1038/s41563-022-01288-0. 36008604 PMC7613626

[39] R. S. Krauss and A. P. Kann , “Muscle Stem Cells Get a New Look: Dynamic Cellular Projections as Sensors of the Stem Cell Niche,” BioEssays 45, no. 5 (2023): 2200249, 10.1002/bies.202200249. PMC10170654 36916774

[40] R. Fukuda , F. Gunawan , R. Ramadass , et al., “Mechanical Forces Regulate Cardiomyocyte Myofilament Maturation via the VCL‐SSH1‐CFL Axis,” Developmental Cell 51, no. 1 (2019): 62–77, 10.1016/j.devcel.2019.08.006. 31495694

[41] M. Cui , S. Bezprozvannaya , T. Hao , et al., “Transcription Factor NFYa Controls Cardiomyocyte Metabolism and Proliferation During Mouse Fetal Heart Development,” Developmental Cell 58, no. 24 (2023): 2867–2880.E7, 10.1016/j.devcel.2023.10.012. 37972593 PMC11000264

[42] E. Bassat , Y. E. Mutlak , A. Genzelinakh , et al., “The Extracellular Matrix Protein Agrin Promotes Heart Regeneration in Mice,” Nature 547 (2017): 179–184, 10.1038/nature22978. 28581497 PMC5769930

[43] W.‐W. Wang , S.‐Y. Ji , W. Zhang , et al., “Structure‐Based Design of Non‐Hypertrophic Apelin Receptor Modulator,” Cell 187, no. 6 (2024): 1460–1475. 38428423 10.1016/j.cell.2024.02.004

[44] K. Bersell , S. Arab , B. Haring , and B. Kuehn , “Neuregulin1/ErbB4 Signaling Induces Cardiomyocyte Proliferation and Repair of Heart Injury,” Cell 138, no. 2 (2009): 257–270, 10.1016/j.cell.2009.04.060. 19632177

[45] M. Yi , H. Li , X. Wong , et al., “Ion Therapy: A Novel Strategy for Acute Myocardial Infarction,” Advanced Science 6, no. 1 (2019): 1801260, 10.1002/advs.201801260. 30643722 PMC6325593

[46] R. Ferrari , A. Albertini , S. Curello , et al., “Myocardial Recovery During Post‐Ischaemic Reperfusion: Effects of Nifedipine, Calcium and Magnesium,” Journal of Molecular and Cellular Cardiology 18, no. 5 (1986): 487–498, 10.1016/S0022-2828(86)80914-2. 3723596

[47] M. N. Sharikabad , K. M. Ostbye , and O. Brørs , “Increased [Mg_2_ ^+^]o Reduces Ca_2_ ^+^ Influx and Disruption of Mitochondrial Membrane Potential During Reoxygenation,” American Journal of Physiology. Heart and Circulatory Physiology 281, no. 5 (2001): H2113–H2123. 11668073 10.1152/ajpheart.2001.281.5.H2113

[48] C. Lian , J. Liu , W. Wei , et al., “Mg‐Gallate Metal‐Organic Framework‐Based Sprayable Hydrogel For Continuously Regulating Oxidative Stress Microenvironment and Promoting Neurovascular Network Reconstruction in Diabetic Wounds,” Bioactive Materials 38 (2024): 181–194, 10.1016/j.bioactmat.2024.04.028. 38711758 PMC11070761

[49] Y. Xu , C. Xu , L. He , et al., “Stratified‐Structural Hydrogel Incorporated With Magnesium‐Ion‐Modified Black Phosphorus Nanosheets For Promoting Neuro‐Vascularized Bone Regeneration,” Bioactive Materials 16 (2022): 271–284, 10.1016/j.bioactmat.2022.02.024. 35386320 PMC8965728

[50] N. Kong , K. Lin , H. Li , and J. Chang , “Synergy Effects of Copper and Silicon Ions on Stimulation of Vascularization by Copper‐Doped Calcium Silicate,” Journal of Materials Chemistry B 2, no. 8 (2014): 1100–1110, 10.1039/C3TB21529F. 32261627

[51] J. Grego‐Bessa , P. Gomez‐Apinaniz , B. Prados , M. J. Gomez , D. MacGrogan , and J. L. de la Pompa , “Nrg1 Regulates Cardiomyocyte Migration and Cell Cycle in Ventricular Development,” Circulation Research 133, no. 11 (2023): 927–943, 10.1161/CIRCRESAHA.123.323321. 37846569 PMC10631509

[52] X. Ai , B. Yan , N. Witman , et al., “Transient Secretion of VEGF Protein From Transplanted hiPSC‐CMs Enhances Engraftment and Improves Rat Heart Function Post MI,” Molecular Therapy 31, no. 1 (2023): 211–229, 10.1016/j.ymthe.2022.08.012. PMC9840120 35982619

[53] K. Huang , E. W. Ozpinar , T. Su , et al., “An Off‐the‐Shelf Artificial Cardiac Patch Improves Cardiac Repair After Myocardial Infarction in Rats and Pigs,” Science Translational Medicine 12, no. 538 (2020): eaat9683. 32269164 10.1126/scitranslmed.aat9683 PMC7293901

[54] S. Miyagawa , T. Kawamura , E. Ito , et al., “Pre‐Clinical Evaluation of the Efficacy and Safety of Human Induced Pluripotent Stem Cell‐Derived Cardiomyocyte Patch,” Stem Cell Research and Therapy 15 (2024): 73, 10.1186/s13287-024-03690-8. 38475911 PMC10935836

[55] R. Qiu , X. Zhang , C. Song , et al., “E‐Cardiac Patch to Sense and Repair Infarcted Myocardium,” Nature communications 15 (2024): 4133–4133, 10.1038/s41467-024-48468-x. PMC11099052 38755124

[56] Y. Que , J. Shi , Z. Zhang , et al., “Ion Cocktail Therapy for Myocardial Infarction by Synergistic Regulation of both Structural and Electrical Remodeling,” Exploration (Beijing, China) 4 (2024): 20230067–20230067. 38939858 10.1002/EXP.20230067 PMC11189571

[57] L. Wang , Y. Liu , G. Ye , et al., “Injectable and Conductive Cardiac Patches Repair Infarcted Myocardium in Rats and Minipigs,” Nature Biomedical Engineering 5 (2021): 1157–1173, 10.1038/s41551-021-00796-9. 34593988

[58] M. A. J. De Smet , A. Lissoni , T. Nezlobinsky , et al., “Cx43 Hemichannel Microdomain Signaling at the Intercalated Disc Enhances Cardiac Excitability,” Journal of Clinical Investigation 131 (2021): e137752, 10.1172/JCI137752. 33621213 PMC8011902

[59] X. Mei , D. Zhu , J. Li , et al., “A Fluid‐Powered Refillable Origami Heart Pouch for Minimally Invasive Delivery of Cell Therapies in Rats and Pigs,” Med 2, no. 11 (2021): 1253–1268, 10.1016/j.medj.2021.10.001. 34825239 PMC8612456

[60] Y. He , H. Hou , S. Wang , et al., “From Waste of Marine Culture to Natural Patch in Cardiac Tissue Engineering,” Bioactive Materials 6, no. 7 (2021): 2000–2010, 10.1016/j.bioactmat.2020.12.011. 33426372 PMC7782558

[61] H. Zhang , C. Qin , M. Zhang , et al., “Calcium Silicate Nanowires‐Containing Multicellular Bioinks for 3D Bioprinting of Neural‐Bone Constructs,” Nano Today 46 (2022): 101584.

[62] G. Kresse and J. Furthmuller , “Efficient Iterative Schemes for *ab Initio* Total‐Energy Calculations Using a Plane‐Wave Basis Set,” Physical Review B 54 (1996): 11169–11186, 10.1103/PhysRevB.54.11169. 9984901

[63] P. E. Blochl , “Projector Augmented‐Wave Method,” Physical Review B 50 (1994): 17953–17979, 10.1103/PhysRevB.50.17953. 9976227

[64] C. Qin , Z. Liao , Z. Wang , H. Zhang , and C. Wu , “Silicate Biomaterials‐Based Multicellular Scaffolds With Specific Cellular Spatial Distribution for Infarcted Myocardium Repair,” Bioactive Materials 56 (2026): 57–76, 10.1016/j.bioactmat.2025.09.039. 41293571 PMC12641195

[65] M. Montgomery , S. Ahadian , L. D. Huyer , et al., “Flexible Shape‐Memory Scaffold For Minimally Invasive Delivery of Functional Tissues,” Nature Materials 16 (2017): 1038–1046, 10.1038/nmat4956. 28805824

