# Supplementary material for: Bioactive Magnesium Silicate Activating Myocardial Energy Metabolism For Infarcted Myocardium Repair
Source: Exploration (Beijing). 2026 Apr 13;6(3):70161. doi: 10.1002/exp2.70161 (PMC13317557; doi:10.1002/exp2.70161)
Supplement: Supplementary file 1 — Supporting File 1: exp270161‐sup‐0001‐SuppMat.docx. [file EXP2-6-70161-s007.docx]

***Supporting Information***

**Bioactive Magnesium Silicate Activating Myocardial Energy Metabolism for Infarcted Myocardium Repair**

**
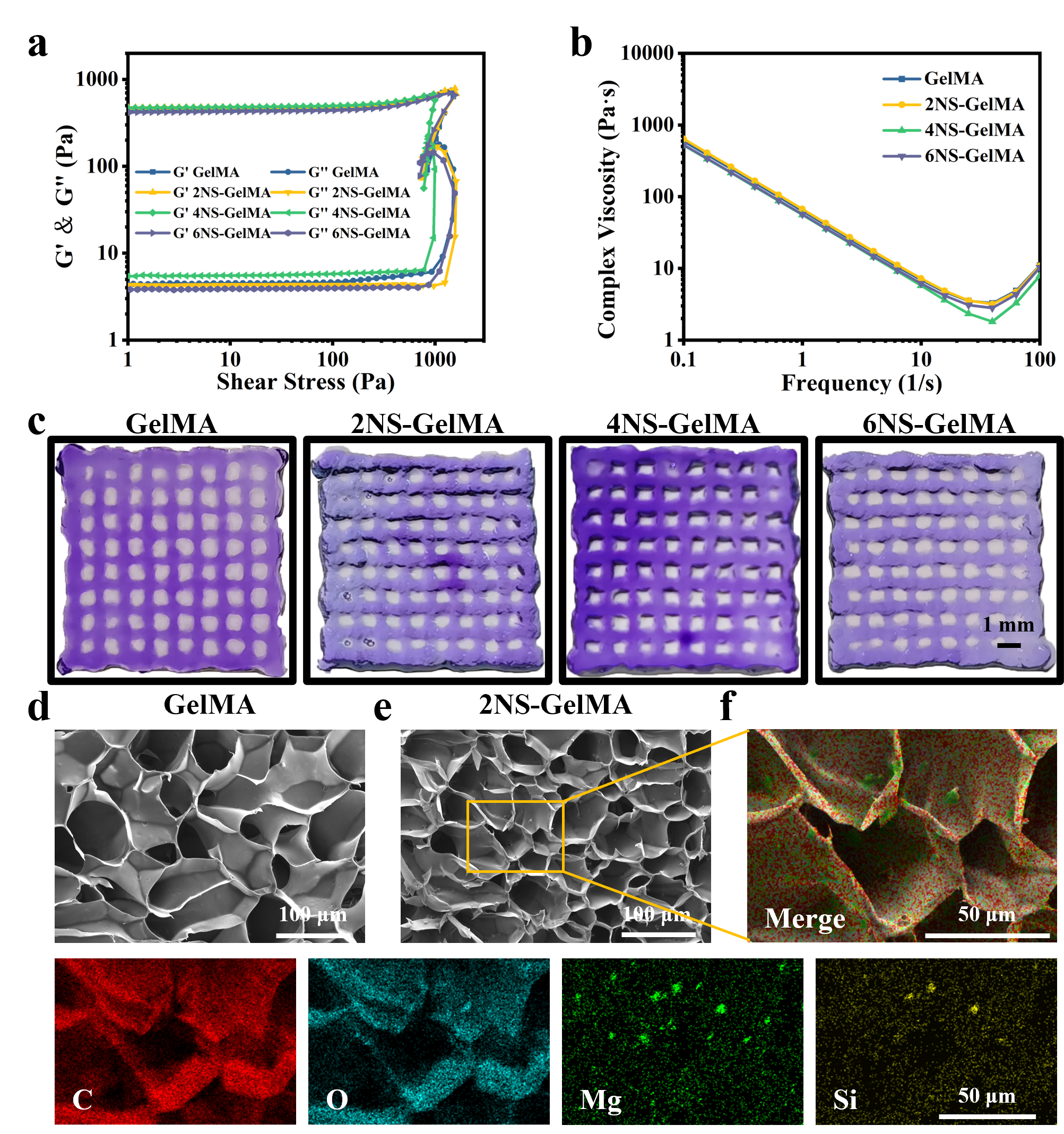
**

**Figure S1. Characterization of the bioinks containing different concentrations of MS nanospheres (NS).** (a) The storage modulus (G′) and loss modulus (G′′) of the bioinks containing different concentrations of NS in the shear stress in the frequency of 1 Hz at 16 ℃. (b) The viscosity of the bioinks containing different concentrations of NS under shear rates of 0.1~100 s^-1^. (c) The 3D printed patches prepared by the bioinks containing different concentrations of NS (All patches were stained with crystal violet). (d) The SEM images of GelMA patches. (e) The SEM images and (f) elements distribution of 2NS-GelMA patches.


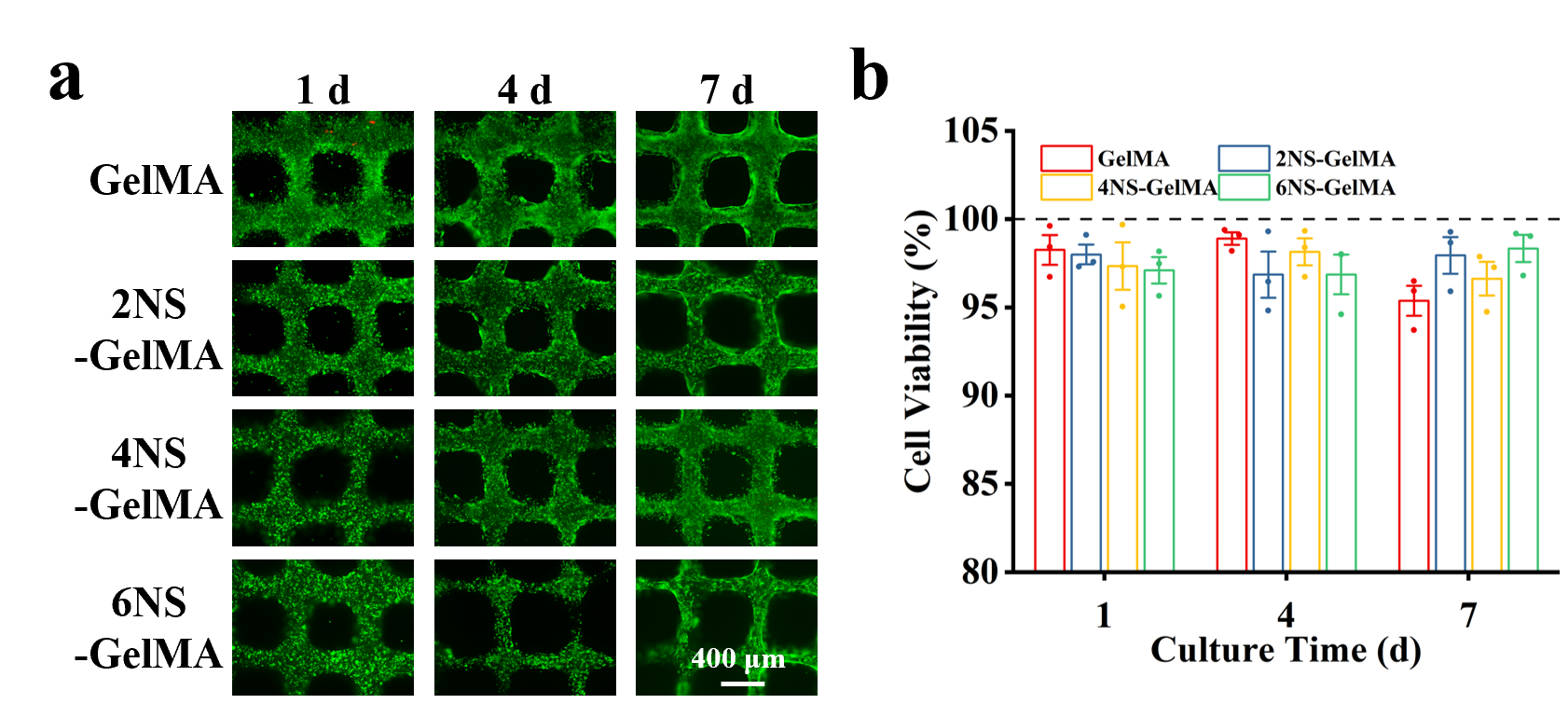


**Figure S2. Cell viability of the 3D bioprinted cardiac patches with different concentrations of NS.** (a) The live/dead staining images of the 3D bioprinted cardiac patches with different concentrations of NS after 1, 4, and 7 days of culture. (b) The viability of the rat cardiomyocytes (rCMs) in the 3D bioprinted cardiac patches with different concentrations of NS (n=3). *P < 0.05, **P < 0.01, or ***P < 0.001.


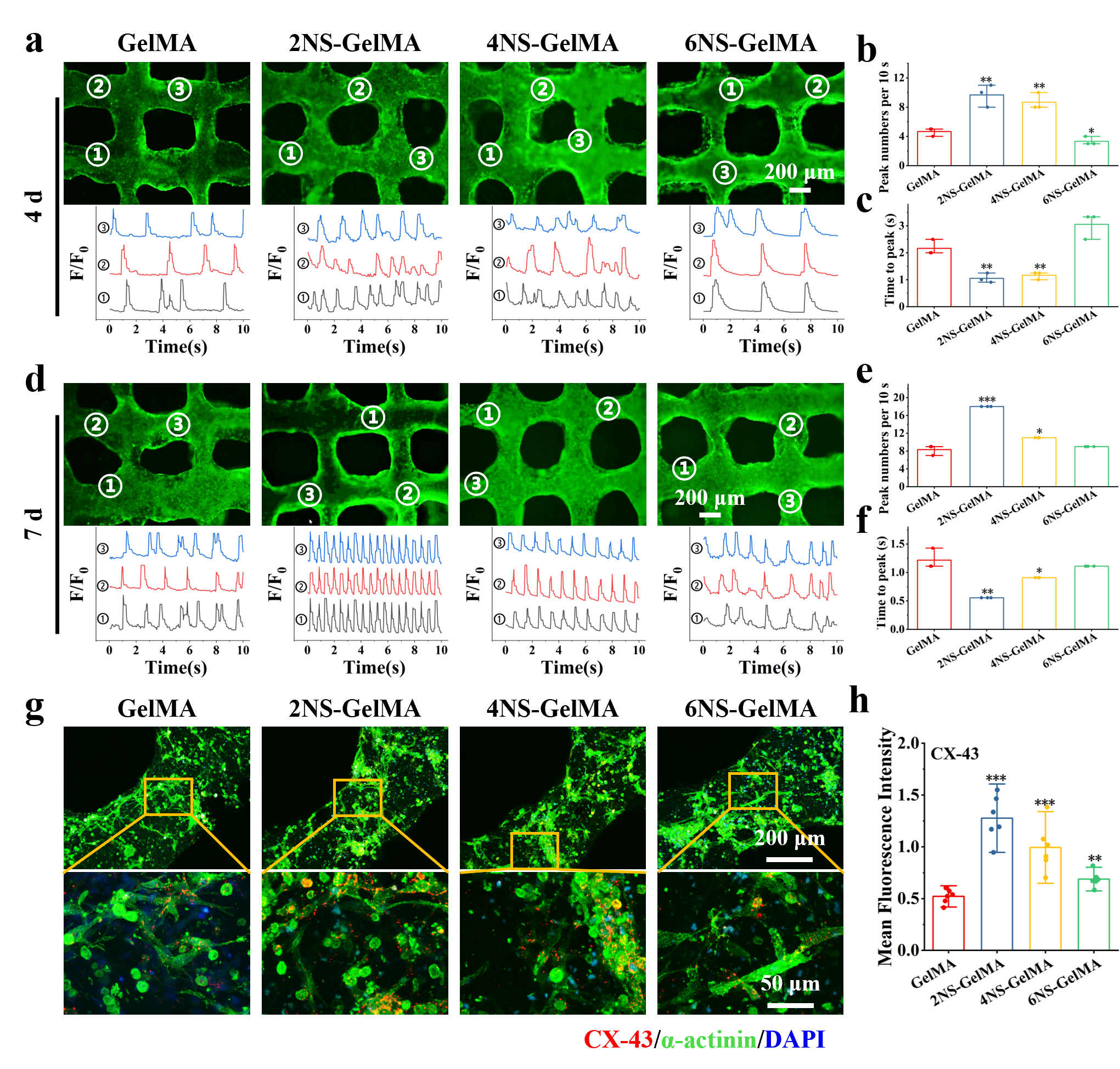


**Figure S3. Characterization of the *in vitro* physiological function of the 3D bioprinted cardiac patches with different concentrations of NS.** (a) Calcium transient images and Ca^2+^ flow signals extracted from three different spots on the 3D bioprinted cardiac patches after 4 days of culture. The corresponding statistical results of (b) peak number per 10s and (c) time to peak according to calcium-transient signals after 4 days of culture (n=3). (d) Calcium transient images and Ca^2+^ flow signals extracted from three different spots on the 3D bioprinted cardiac patches after 7 days of culture. The corresponding statistical results of (e) peak numbers per 10s and (f) time to peak according to calcium-transient signals after 7 days of culture (n=3). (g) The immunostaining images of cardiac-specific proteins connexin43 (CX-43, red), and α-actinin (green) of the 3D bioprinted cardiac patches after 7 days of culture. (h) The semi-quantitative statistical result of the CX-43 expression (n=6). *P < 0.05, **P < 0.01, or ***P < 0.001. **The maturation and synchronous contraction function of the 3D bioprinted cardiac patches were improved by the bioinks containing 2% and 4% NS.**

**
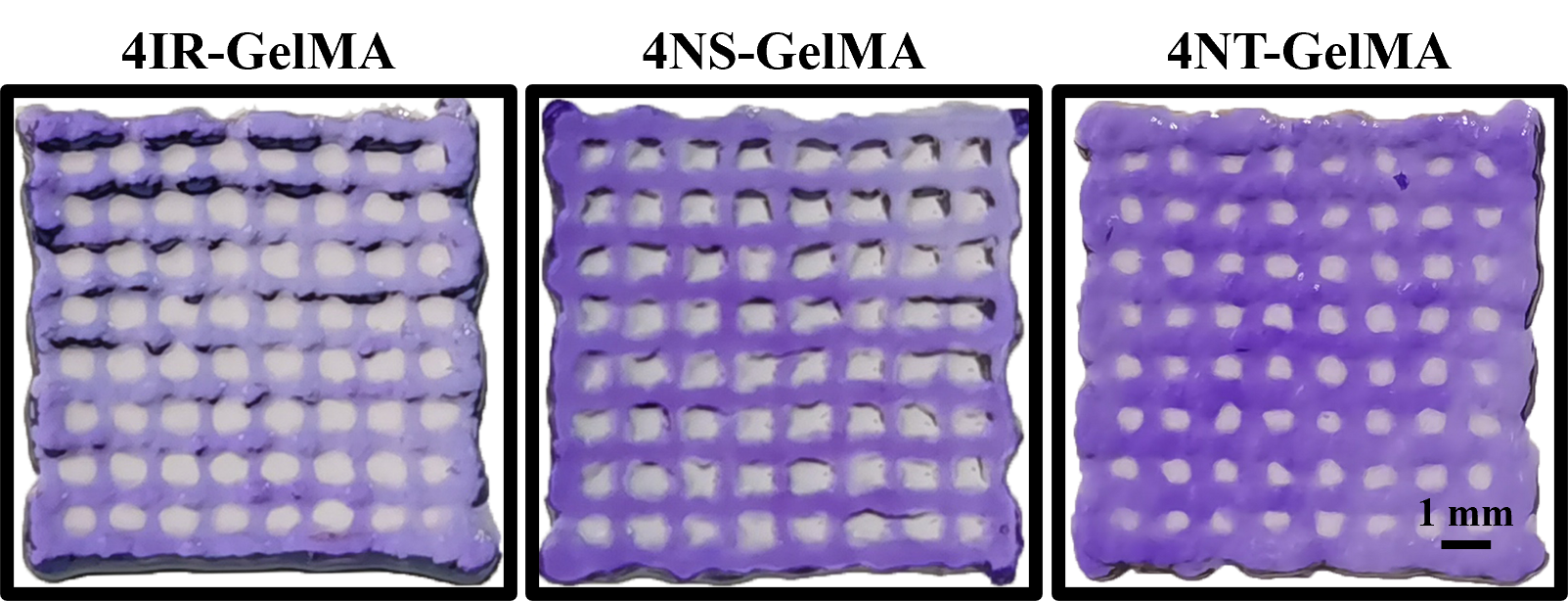
**

**Figure S4.** The 3D printed patches prepared by the bioinks containing 4% MS with different morphologies (All patches were stained with crystal violet).

**
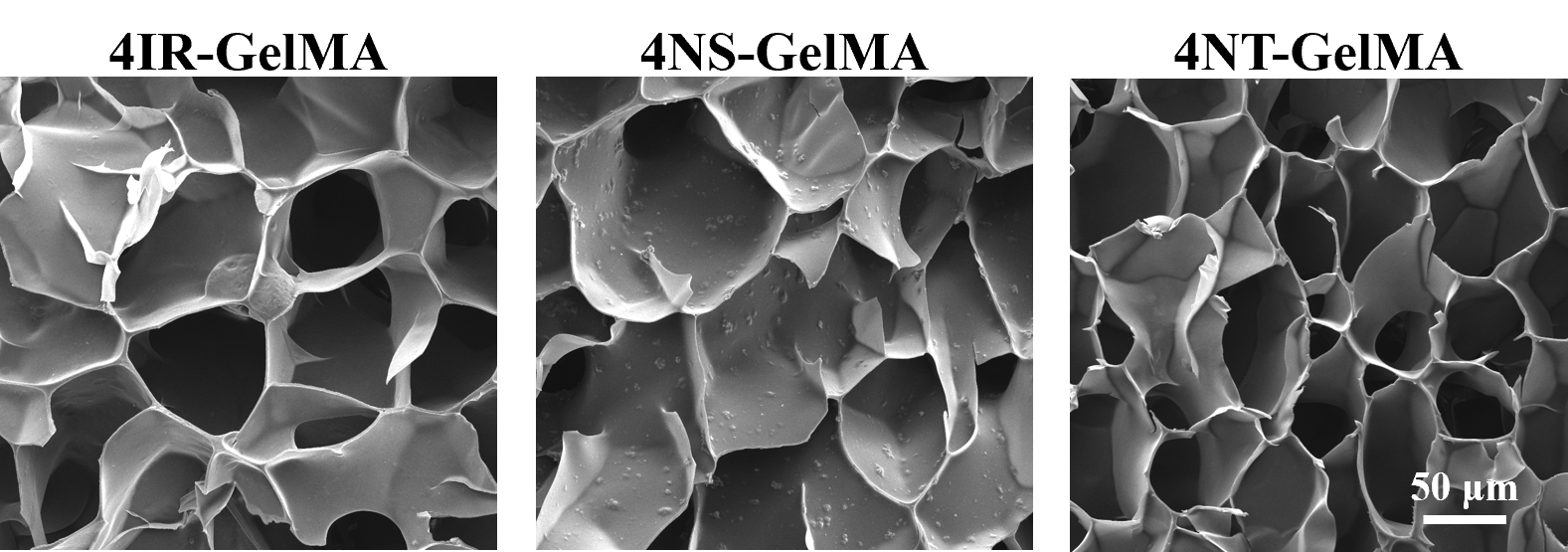
**

**Figure S5.** The SEM images of the 3D printed patches prepared by the bioinks containing 4% MS with different morphologies.


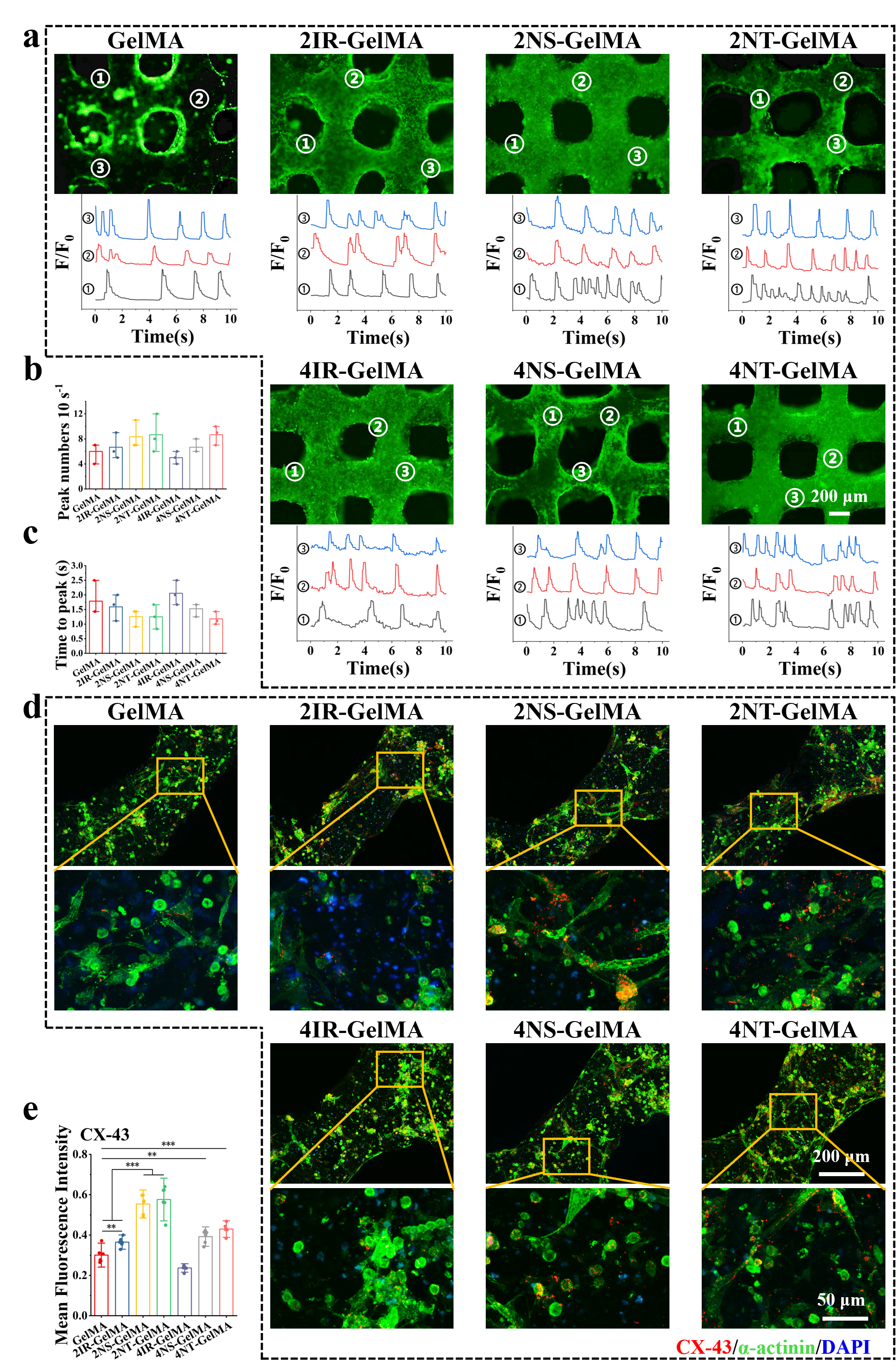


**Figure S6. The *in vitro* physiological functions of 3D bioprinted cardiac patches with different morphologies of MS nanoparticles after 4 days of culture.** (a) Calcium transient images and Ca^2+^ flow signals extracted from three different spots of the cardiac patches with 2% and 4% IR, NS, and NT. The corresponding quantitative results of (b) peak numbers per 10s and (c) time to peak according to the calcium-transient signals (n=3). (d) The immunostaining images of cardiac-specific proteins connexin43 (CX-43, red), and α-actinin (green) of the cardiac patches with 2% and 4% IR, NS, and NT. (e) The semi-quantitative statistical result of the CX-43 expression after 4 days of culture (n=6). *P < 0.05, **P < 0.01, or ***P < 0.001.


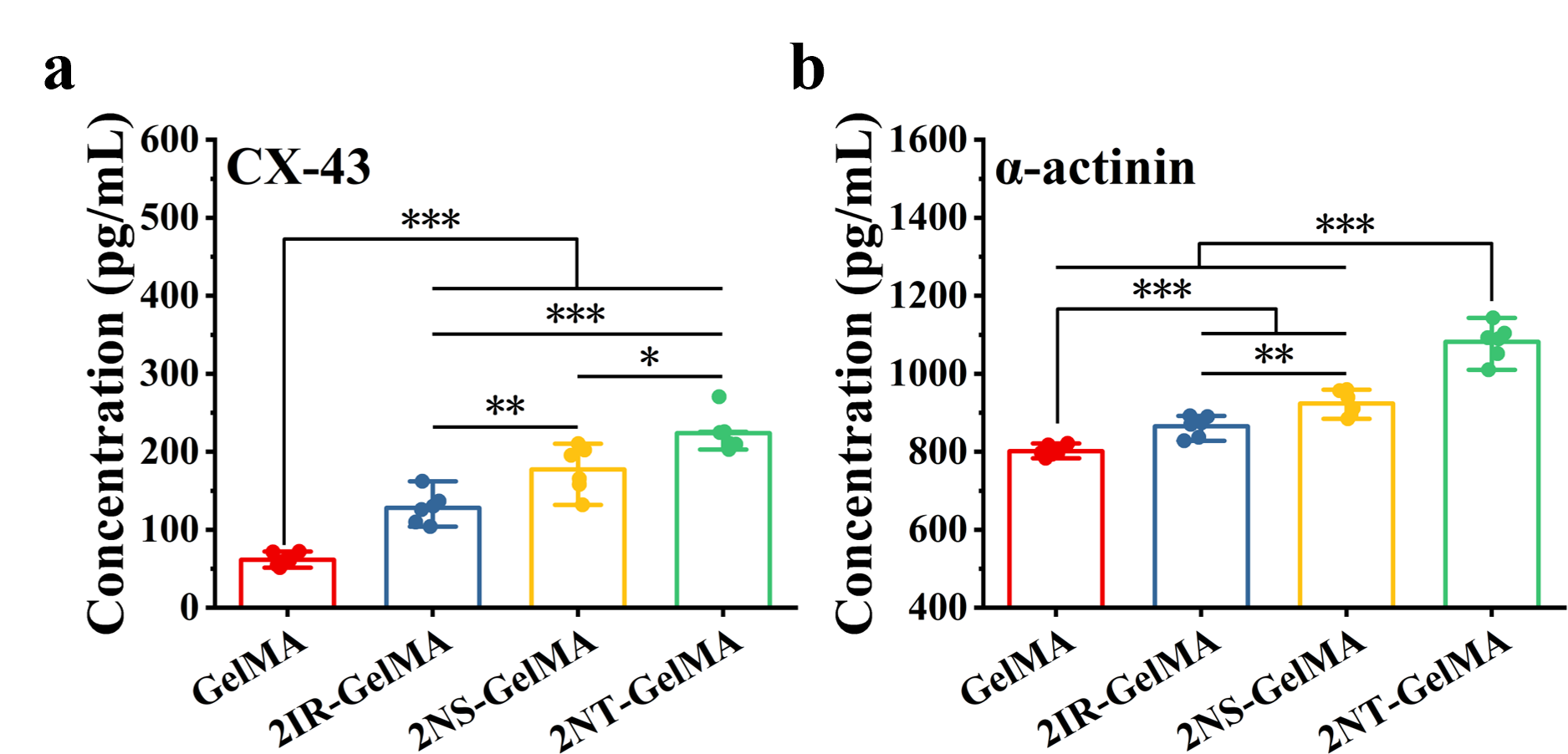


**Figure S7.** ELISA analyses of the protein-levels of (a) CX-43 and (b) α-actinin of the CMs loaded in the cardiac patches (n=6). *P < 0.05, **P < 0.01, or ***P < 0.001.


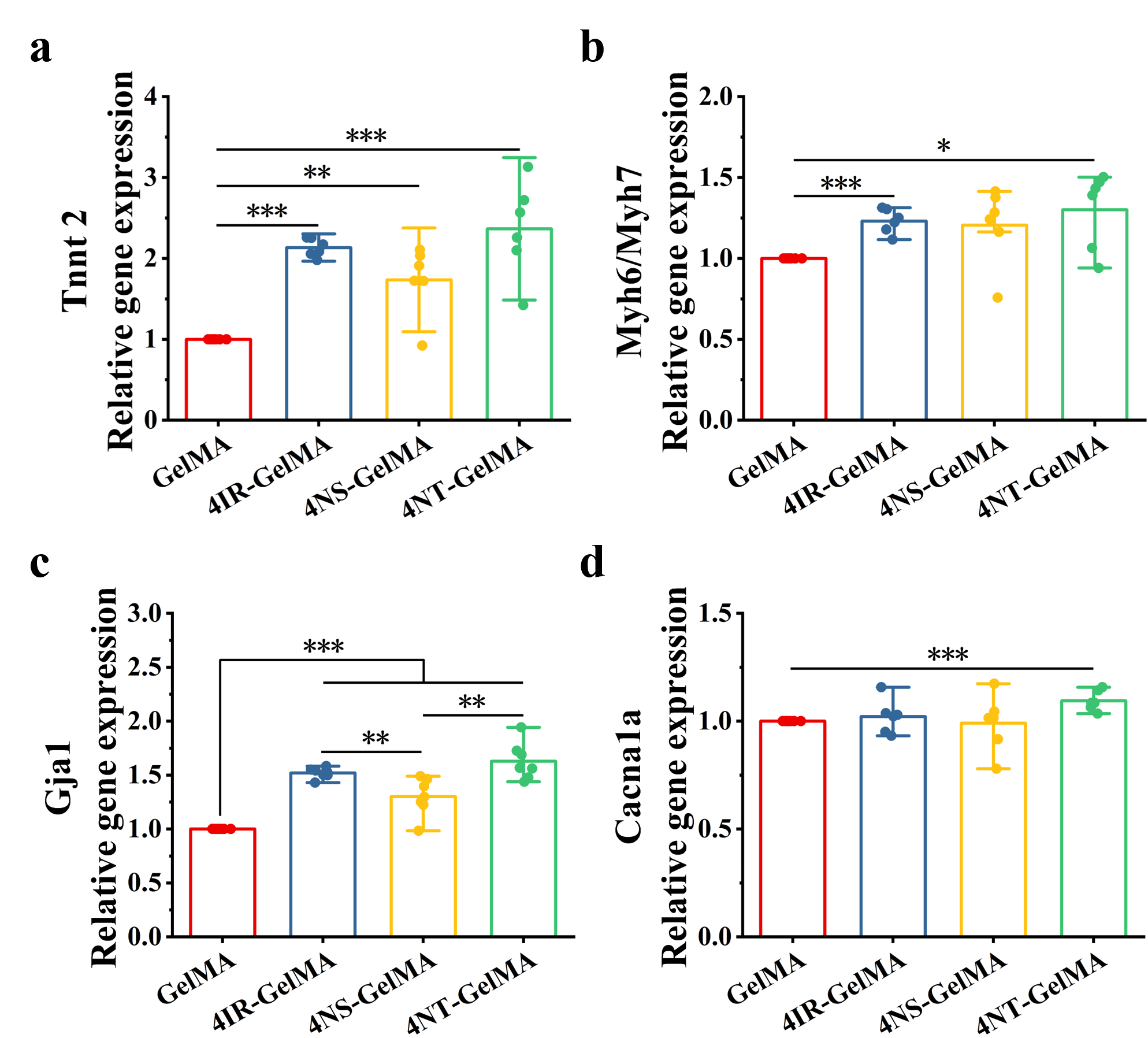


**Figure S8.** The relative expression of (a) Tnnt2, (b) Myh6/Myh7, (c) Gja1, (d) Cacna1a of rCMs within these 3D bioprinted cardiac patches (n=6). *P < 0.05, **P < 0.01, or ***P < 0.001.

**
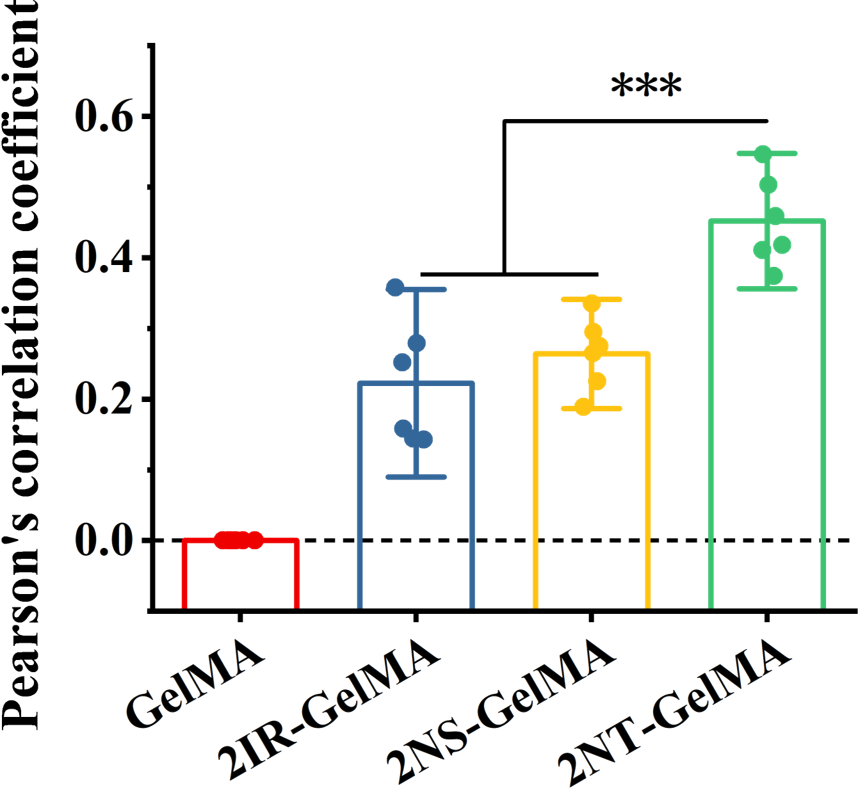
**

**Figure S9.** The person’s correlation coefficient related to the co-localization of the mitochondria and MS nanoparticles with different morphologies (n=6). *P < 0.05, **P < 0.01, or ***P < 0.001.


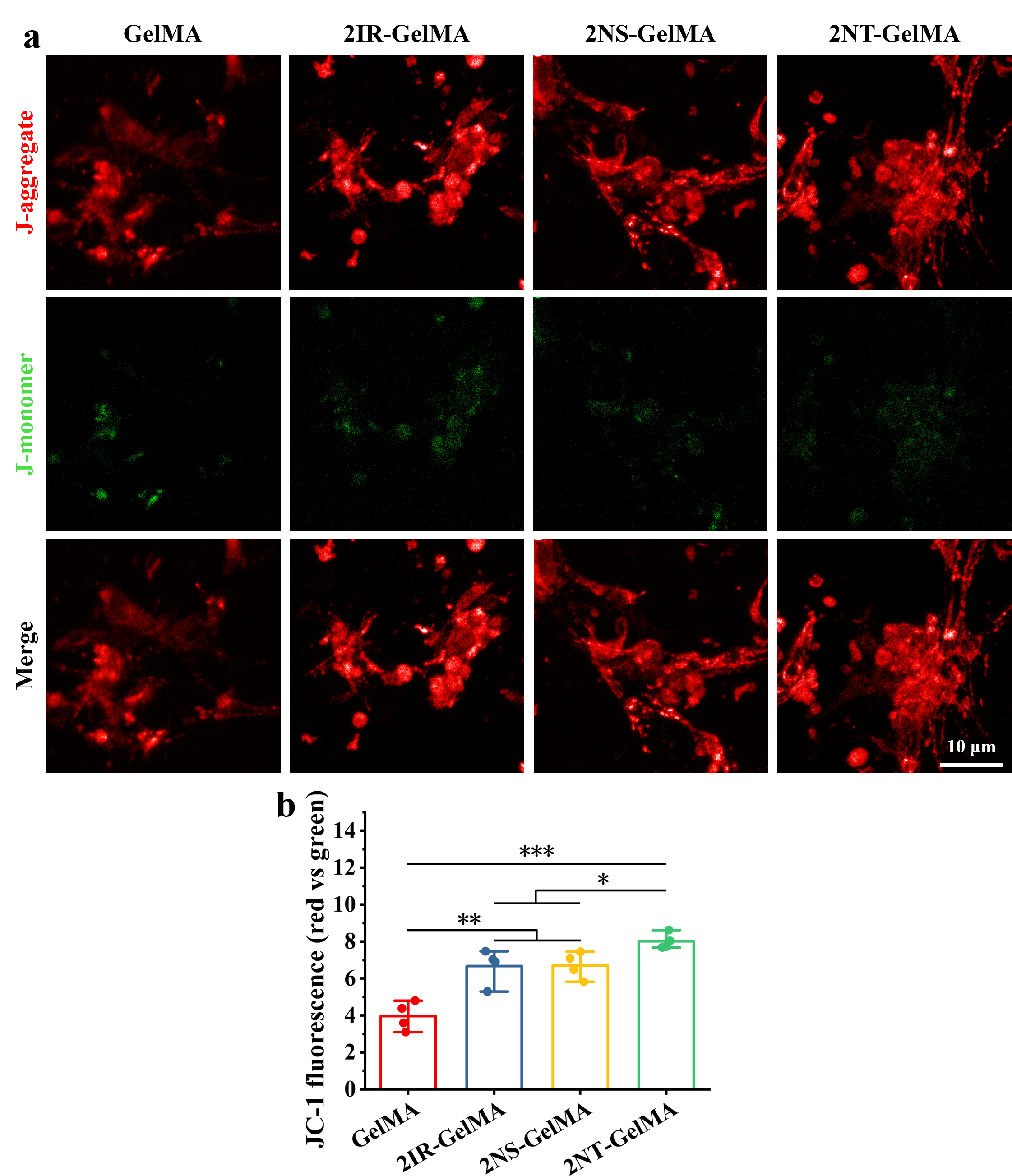


**Figure S10.** (a) Representative immunofluorescence images of JC-1 staining assessing mitochondrial membrane potential in cardiac patches. (b) Statistical results of the JC-1 aggregate/monomer ratio (n=4). *P < 0.05, **P < 0.01, or ***P < 0.001.

**
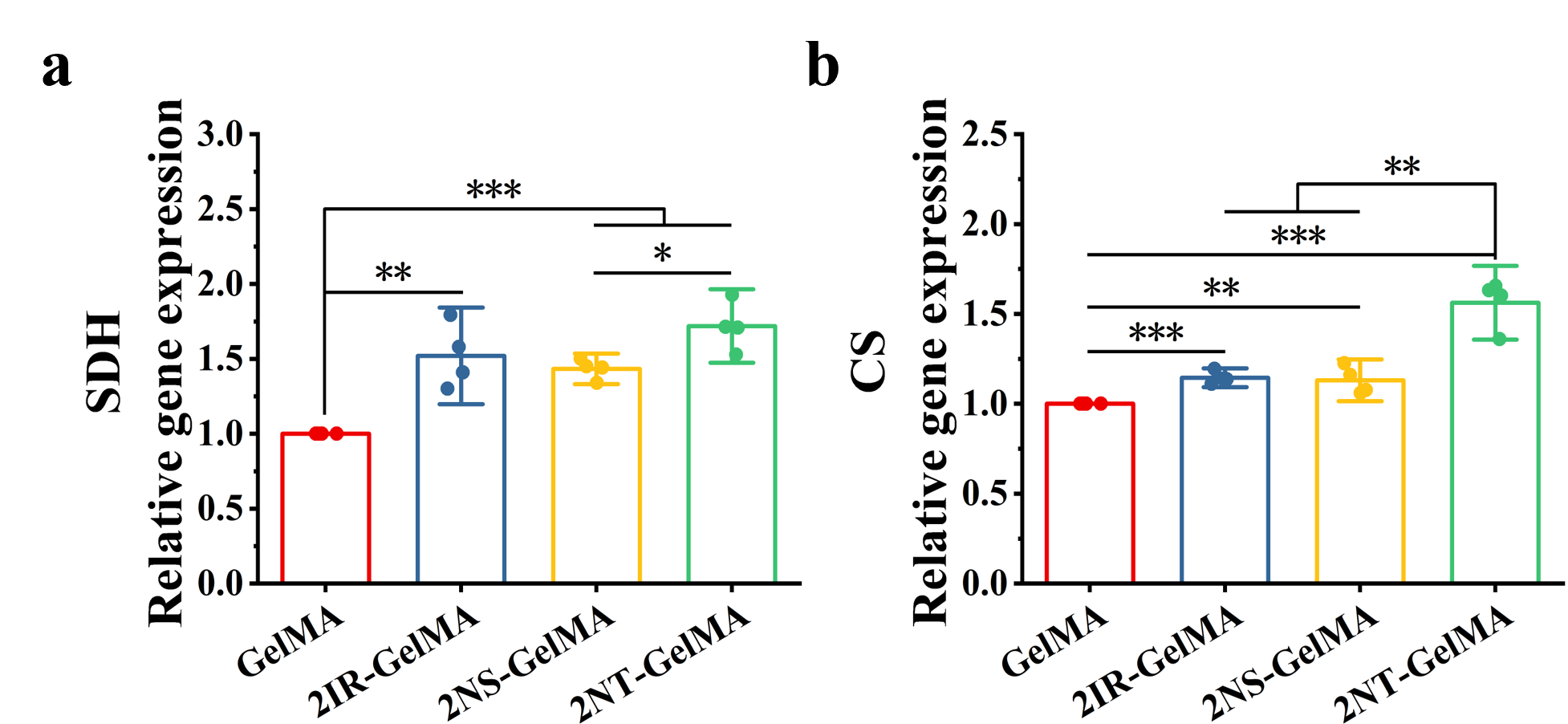
**

**Figure S11.** The relative gene expression of (a) SDH and (b) CS of rCMs within these 3D bioprinted cardiac patches (n=4). *P < 0.05, **P < 0.01, or ***P < 0.001.


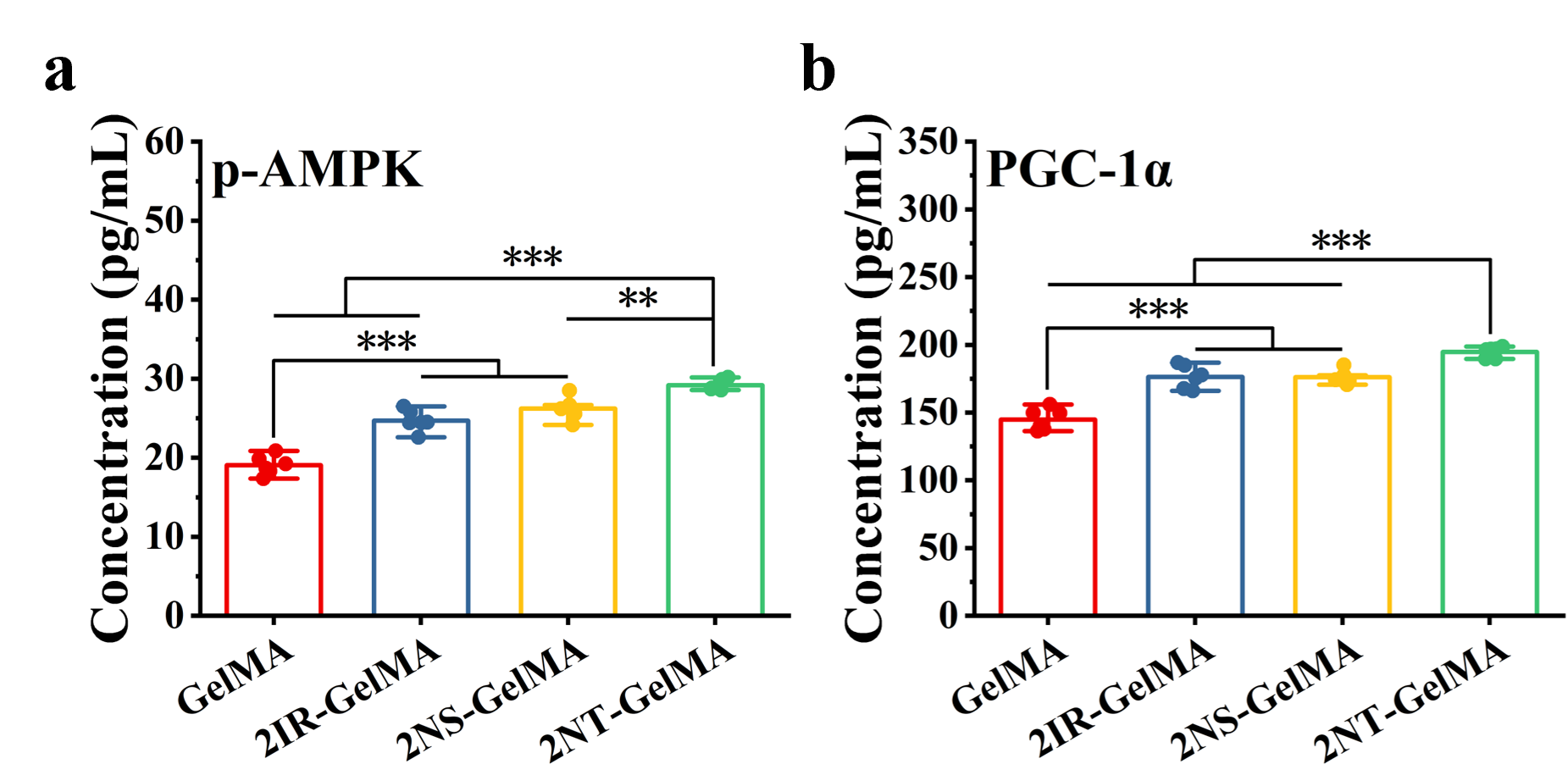


**Figure S12.** ELISA analyses of the protein-levels of (a) p-AMPK and (b) PGC1-α of the rCMs loaded in the cardiac patches (n=6). *P < 0.05, **P < 0.01, or ***P < 0.001.

**
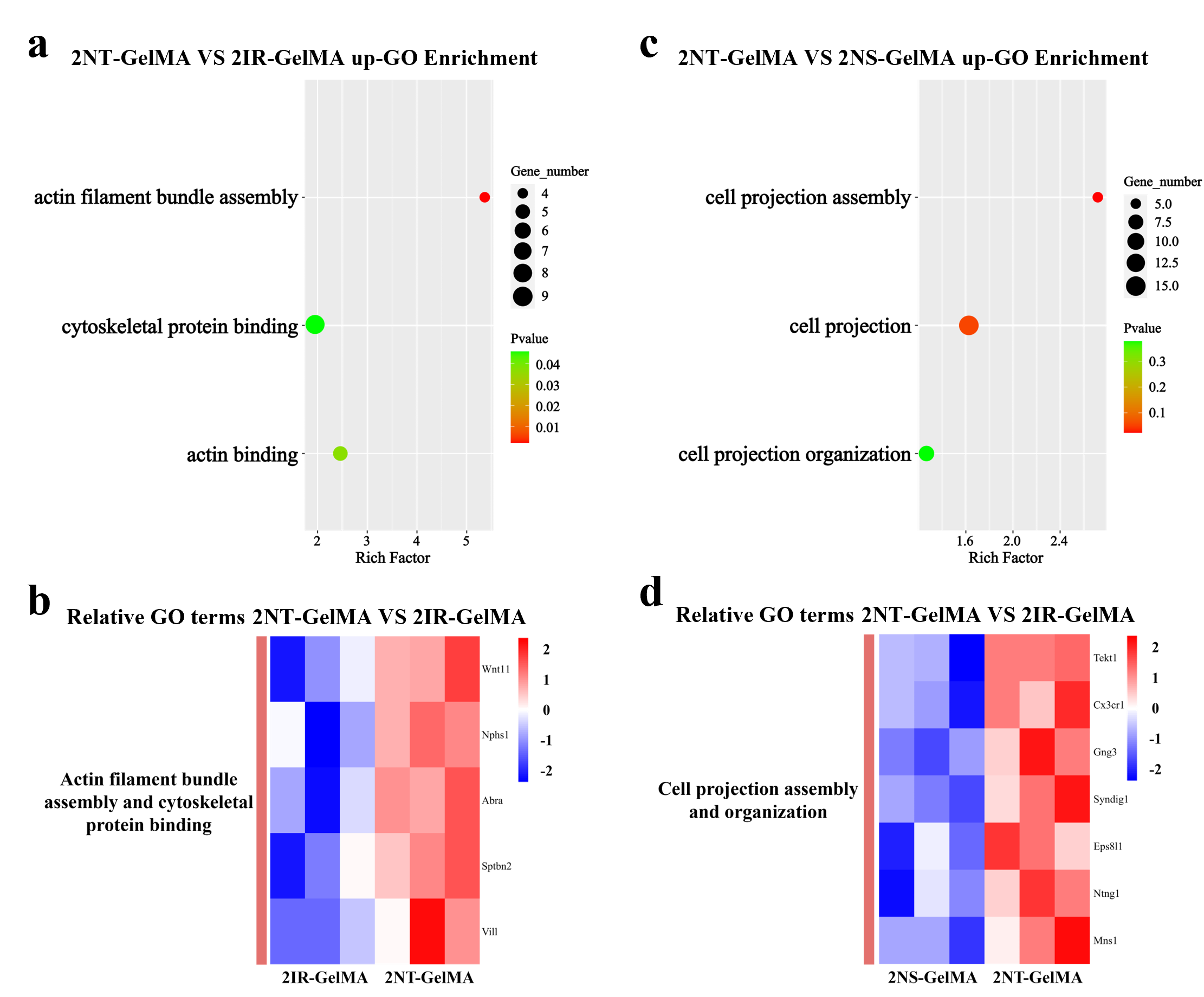
**

**Figure S13. RNA-Sequence analysis of the rCMs in the 3D bioprinted cardiac patches after 7 days of culture, comparing the 2NT-GelMA group and the 2IR-GelMA, 2NS-GelMA groups, related to sensing the dynamic mechanical environment.** (a) Significant up-Gene Ontology (GO) terms enriched by the up-regulated genes of 2NT-GelMA versus 2IR-GelMA. (b) Heatmap of gene expression levels related to the GO terms of actin filament bundle assembly and cytoskeletal protein binding (2NT-GelMA versus 2IR-GelMA). (c) Significant up-GO terms enriched by the up-regulated genes of 2NT-GelMA versus 2NS-GelMA. (d) Heatmap of gene expression levels related to the GO terms of cell projection assembly and organization (2NT-GelMA versus 2NS-GelMA).

**
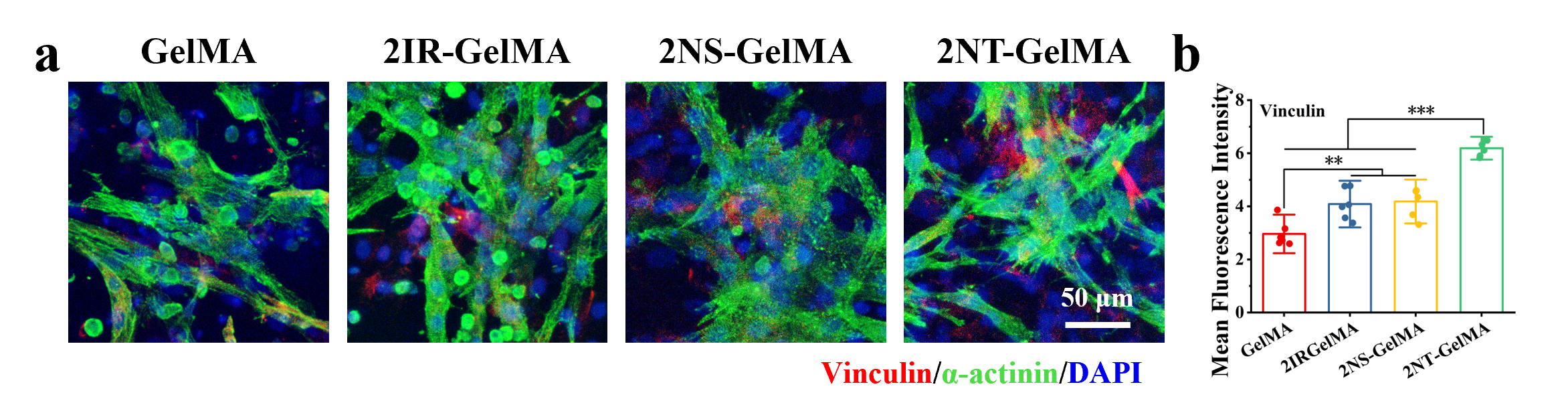
**

**Figure S14.** (a) Immunostaining images of the mechanosensitive protein Vinculin (red) and the cardiac-specific protein α-actinin (green) in cardiac patches containing 2% IR, NS, and NT. (b) Quantitative analysis of Vinculin expression after 7 days of culture (n=6). **P < 0.01, ***P < 0.001.


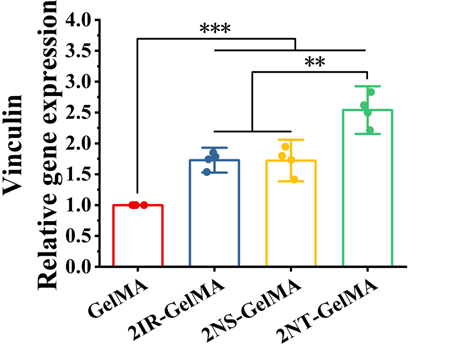


**Figure S15.** The relative mRNA expression of the mechanosensitive protein Vinculin of rCMs within these 3D bioprinted cardiac patches (n=4).**P < 0.01, or ***P < 0.001.


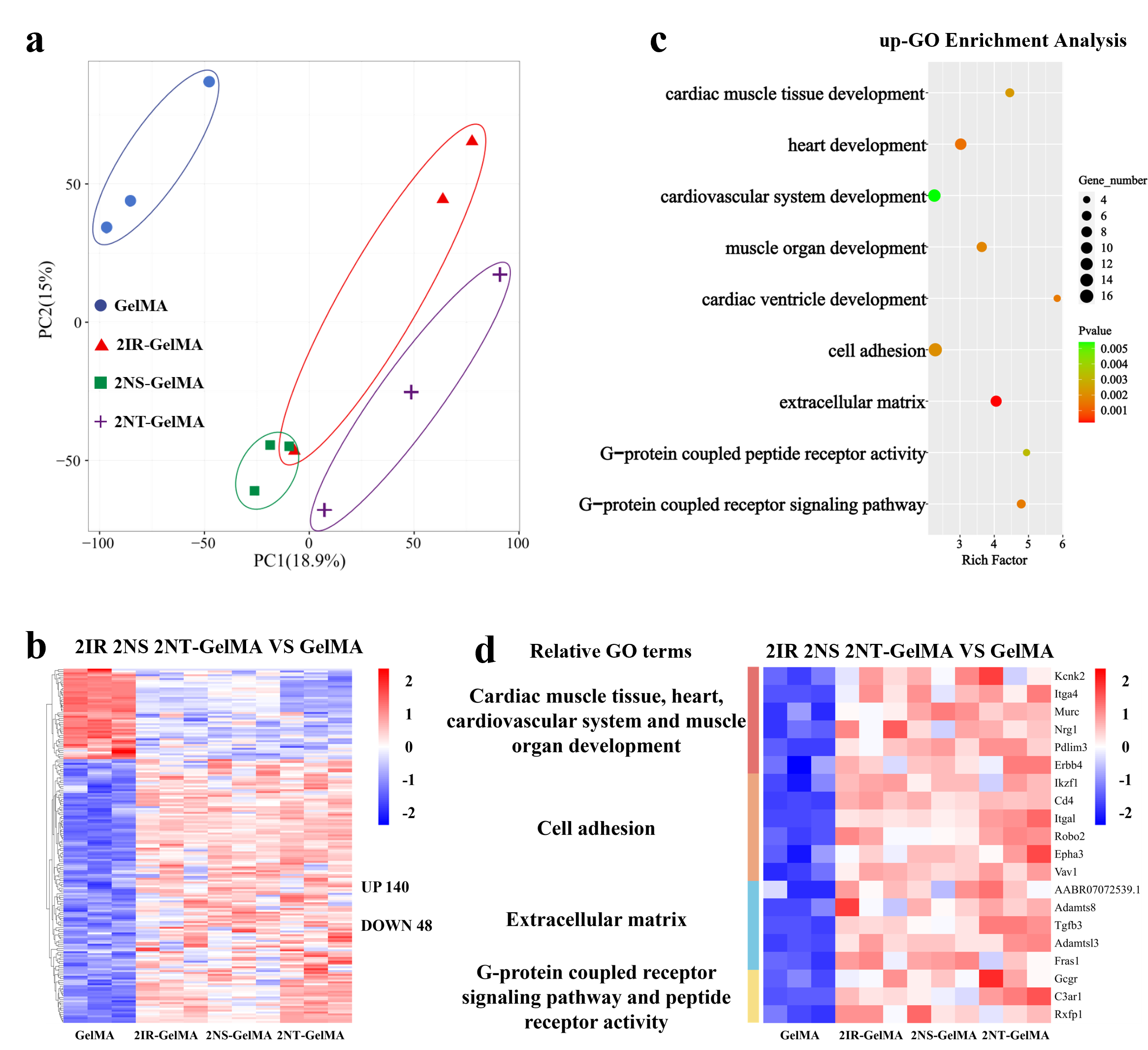


**Figure S16. RNA-Sequence analysis of the rCMs in the 3D bioprinted cardiac patches after 7 days of culture by comparing the three MS-containing groups (2IR-GelMA, 2NS-GelMA, 2NT-GelMA) and the GelMA group.** (a) Principal component analysis (PCA) of the four groups. (b) The cluster heatmap of DEGs, illustrating the common differential genes between the three MS-containing groups (2IR-GelMA, 2NS-GelMA, 2NT-GelMA) and the GelMA group. (c) Significant up-GO terms enriched by the up-regulated genes of 2IR-GelMA, 2NS-GelMA, 2NT-GelMA versus GelMA. (d) Heatmap of gene expression levels in the GO terms that related to cardiac development, cardiac maturation and cardiac protection (2IR-GelMA, 2NS-GelMA, 2NT-GelMA versus GelMA).


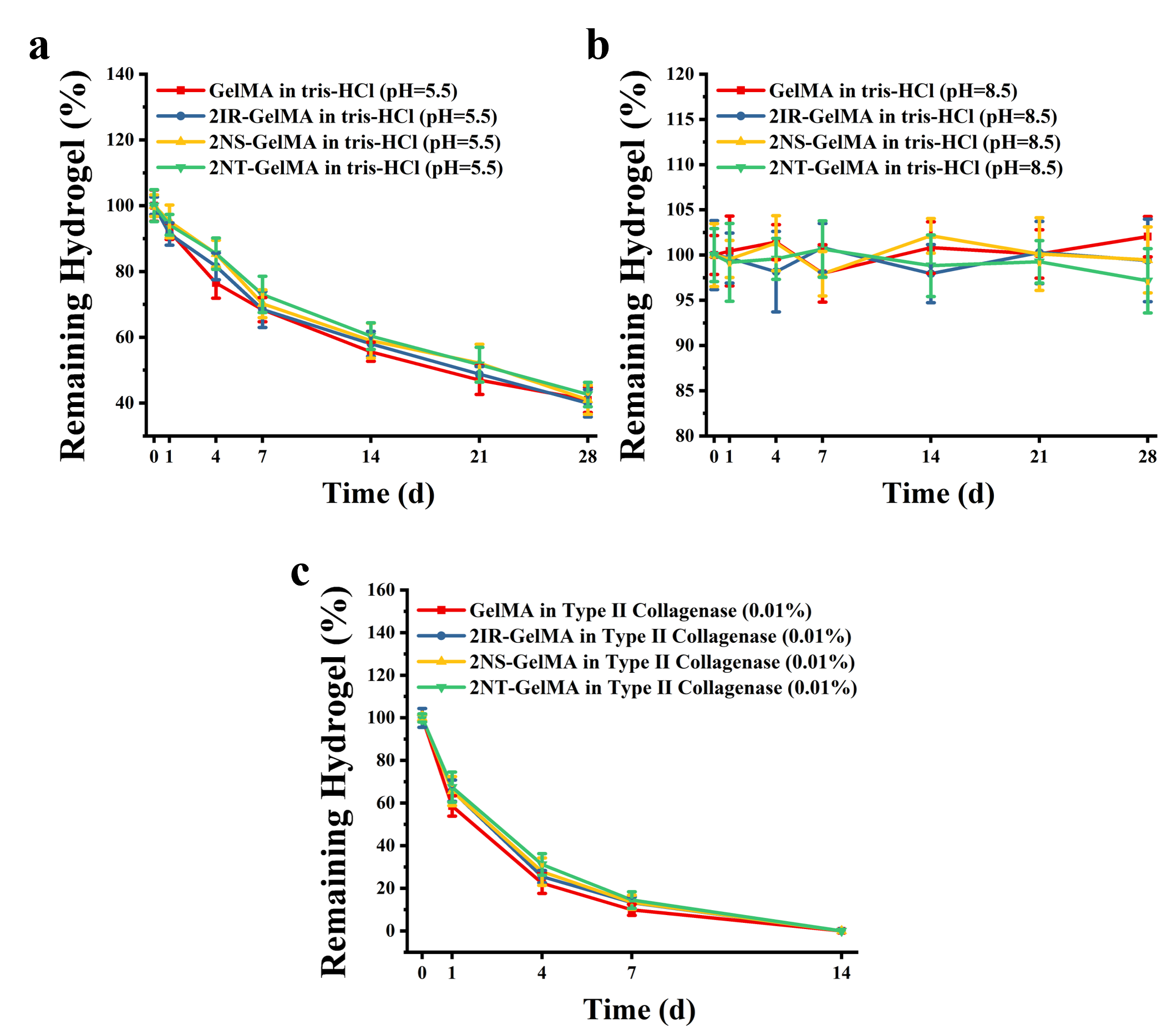


**Figure S17.** Degradation behavior of GelMA, 2IR-GelMA, 2NS-GelMA, and 2NT-GelMA hydrogels under different conditions: (a) acidic (pH 5.5), (b) basic (pH 8.5), and (c) enzymatic (0.01% Type II collagenase) (n=3).


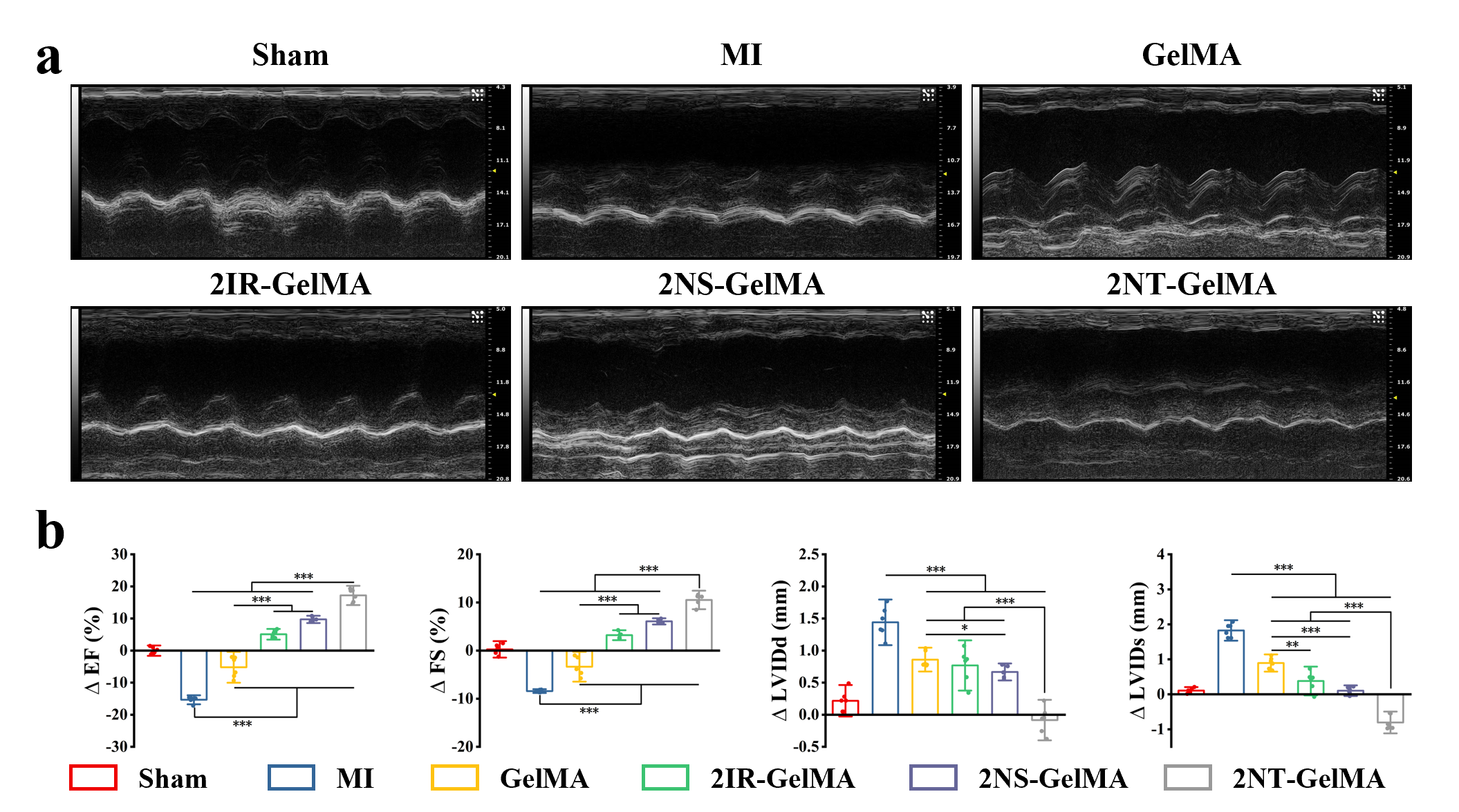


**Figure S18.** (a) Representative echocardiograms of rat hearts at 1 week post-surgery. (b) Statistical results of the changes of the EF, FS, LVIDd, and LVIDs from 1 week to 4 weeks post-surgery according to the echocardiography results (n=6). *P < 0.05, **P < 0.01, or ***P < 0.001.


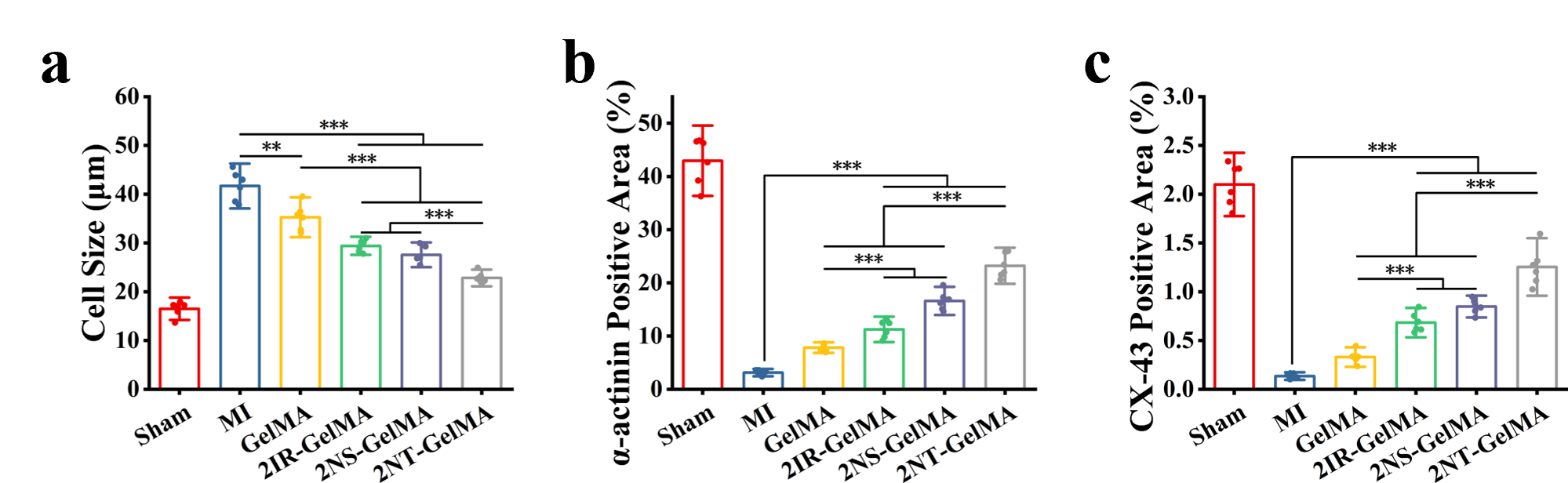


**Figure S19.** (a) Statistical results of cardiomyocyte size according to WGA immunofluorescent staining images (n=6). (b) Statistical results of α-actinin area in the infarction regions of rat heart sections at 4 weeks post-surgery (n=6). (c) Statistical results of CX-43 area in the infarction regions of rat heart sections at 4 weeks post-surgery (n=6). *P < 0.05, **P < 0.01, or ***P < 0.001.


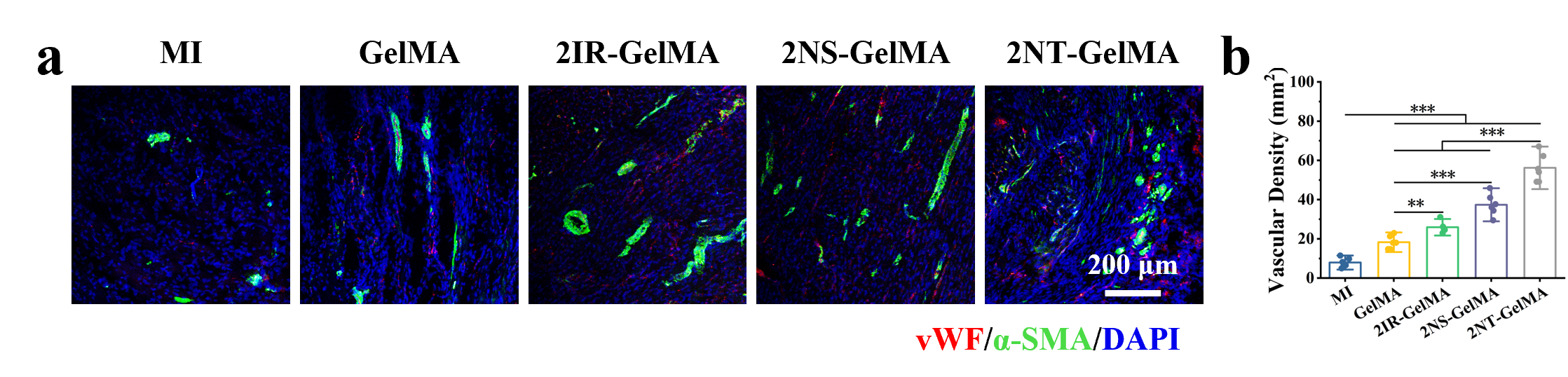


**Figure S20.** (a) Immunofluorescent staining images of von willebrand factor (vWF) and α-smooth muscle actin (α-SMA) in the infarction regions of rat heart sections at 4 weeks post-surgery (red: vWF; green: α-SMA; blue: DAPI). (b) Statistical results of vascular density in the infarction regions of rat heart sections at 4 weeks post-surgery (n=6). *P < 0.05, **P < 0.01, or ***P < 0.001.


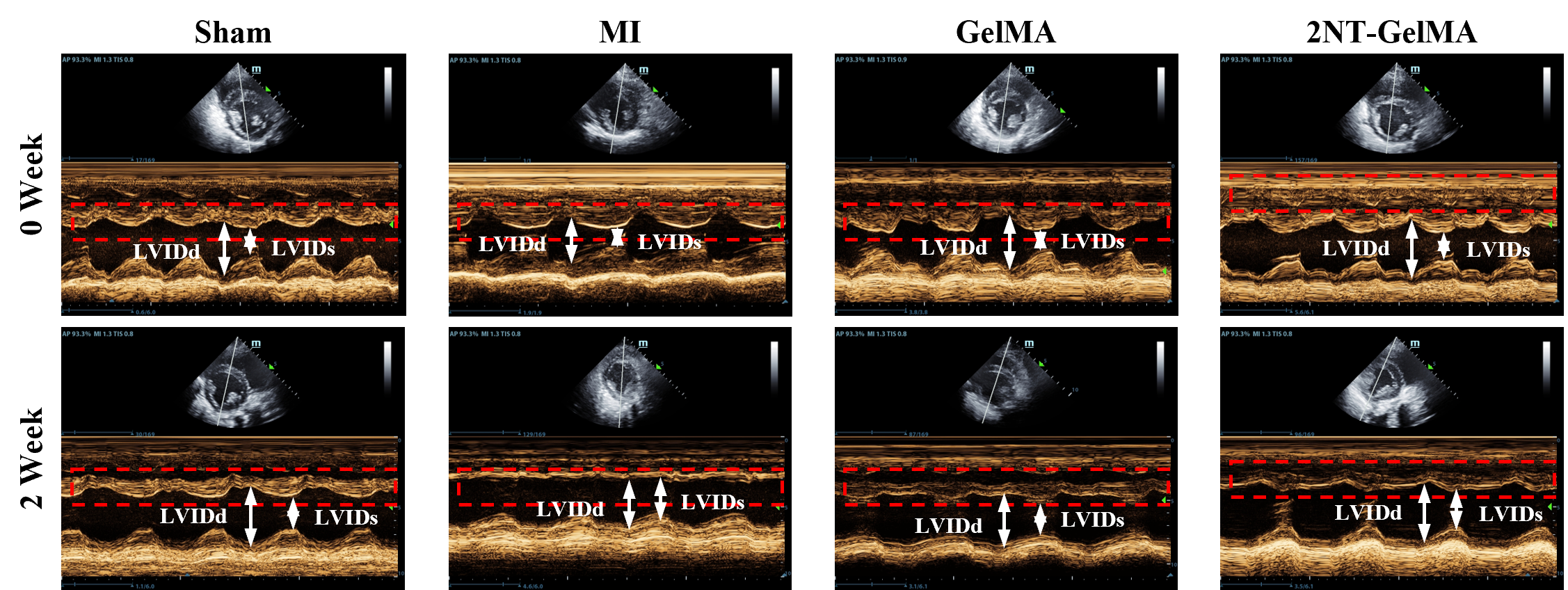


**Figure S21.** Representative echocardiographic images of minipig hearts before surgery and at 2 weeks post-surgery.


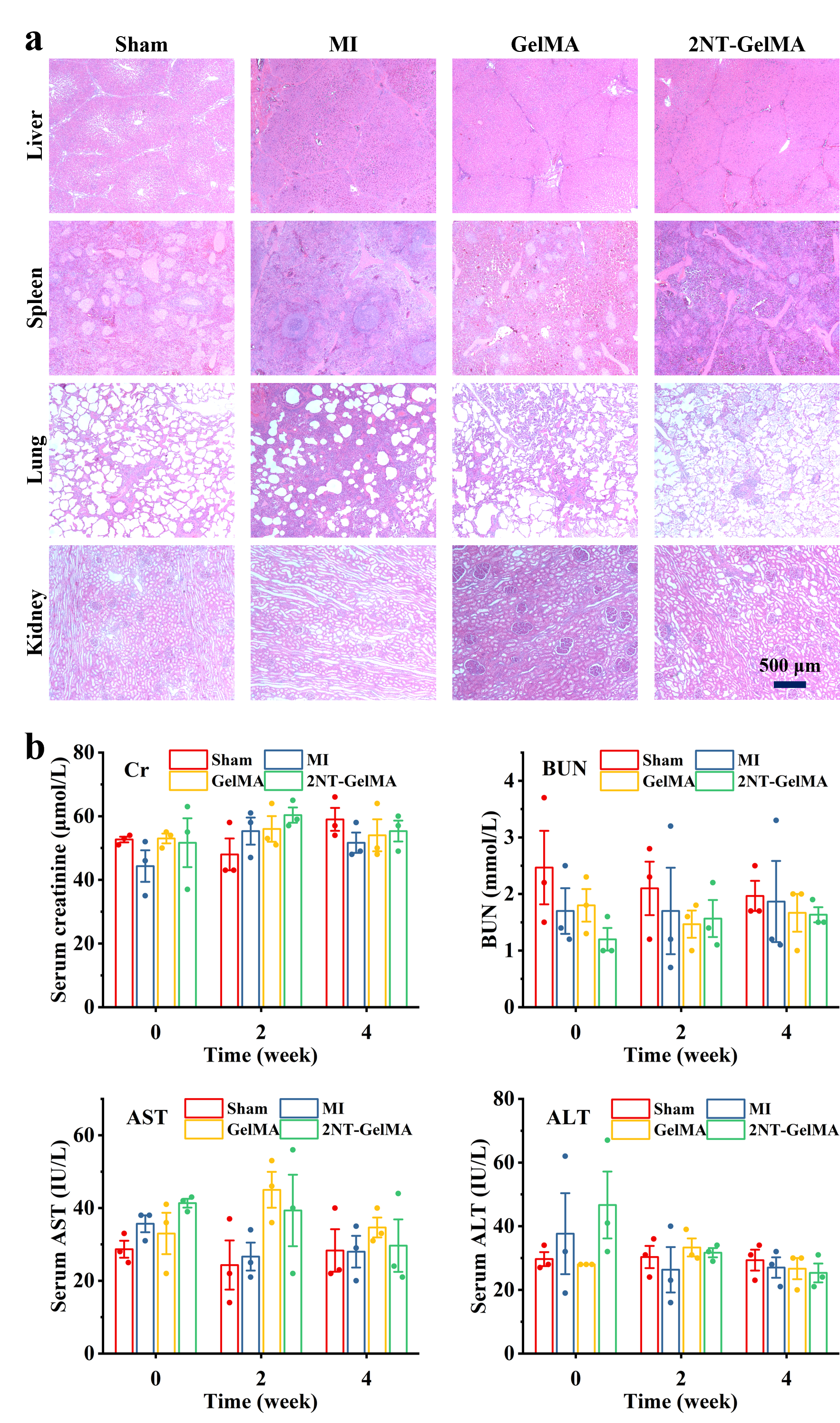


**Figure S22. *In vivo* safety assessment in minipigs.** (a) H&E staining images of the liver, spleen, lung, and kidney of minipigs with different treatments at 4 weeks post-surgery. (b) The concentration of kidney functions-related indicators (serum creatinine (Cr), blood urea nitrogen (BUN)) and liver functions-related indicators (serum aspartate aminotransferase (AST), alanine transaminase (ALT)) in the blood of minipigs at 0, 2 and 4 weeks post-surgery (n=3). *P < 0.05, **P < 0.01, or ***P < 0.001.

**Table S1.** The primer sequences used in RT-qPCR assays.

| **Gene** | **Primer sequences** |
| --- | --- |
| GAPDH | 5’-GGCATCGTGGAAGGGCTCAT-3’ (forward) |
| GAPDH | 5’-GGGATGACCTTGCCCACAG-3’ (reverse) |
| Tnnt2 | 5’-ATGATGCATTTTGGGGGTTA-3’ (forward) |
| Tnnt2 | 5’-CAGCACCTTCCTCCTCTCAG-3’ (reverse) |
| Myh6 | 5’-TCAAGCGGGAGAACAAGAACCT-3’ (forward) |
| Myh6 | 5’-CTCCAGCTCGTGCACATTTTTAC-3’ (reverse) |
| Myh7 | 5’-ACCCCTACGATTATGCG-3’ (forward) |
| Myh7 | 5’-GTGACGTACTCGTTGCC-3’ (reverse) |
| Gja1 | 5’-GCTATGACAAGTCTTTCCCA-3’ (forward) |
| Gja1 | 5’-CAGTTTCTCTTCCTTTCGCA-3’ (reverse) |
| Cacna1a | 5’-CTCTTGCGGATGGACCTACC-3’ (forward) |
| Cacna1a | 5’-GCTCCACCCTTTGCGATTTTGATA-3’ (reverse) |
| Vinculin | 5’-ACCCTACAGTGGATGACCGA-3’ (forward) |
| Vinculin | 5’-TCTACACGGTCACACTTGGC-3’ (reverse) |
| CS | 5’-GCCAAGAACTCATCCTGCCT-3’ (forward) |
| CS | 5’-GTCTTCCCATGCTGCTGTCT-3’ (reverse) |
| SDH | 5’-AGCTGTACTTCTGCCCACAC-3’ (forward) |
| SDH | 5’-GTGGGAAGGACTGGAATGGG-3’ (reverse) |

**Supplementary Videos:**

Supplementary Video 1. The contraction of the cardiac patches (GelMA, 2NS-GelMA, 4NS-GelMA and 6NS-GelMA) on day 4 and day 7.

Supplementary Video 2. The calcium transient of the cardiac patches (GelMA, 2NS-GelMA, 4NS-GelMA and 6NS-GelMA) on day 4 and day 7.

Supplementary Video 3. The contraction of the cardiac patches (GelMA, 2IR-GelMA, 2NS-GelMA, 2NT-GelMA, 4IR-GelMA, 4NS-GelMA and 4NT-GelMA) on day 4.

Supplementary Video 4. The calcium transient of the cardiac patches (GelMA, 2IR-GelMA, 2NS-GelMA, 2NT-GelMA, 4IR-GelMA, 4NS-GelMA and 4NT-GelMA) on day 4.

Supplementary Video 5. The contraction of the cardiac patches (GelMA, 2IR-GelMA, 2NS-GelMA, 2NT-GelMA, 4IR-GelMA, 4NS-GelMA and 4NT-GelMA) on day 7.

Supplementary Video 6. The calcium transient of the cardiac patches (GelMA, 2IR-GelMA, 2NS-GelMA, 2NT-GelMA, 4IR-GelMA, 4NS-GelMA and 4NT-GelMA) on day 7.

Supplementary Video 7. The myocardial surface exhibited a dark purple color and ST-segment elevation on the electrocardiogram after the MI model was established.

Supplementary Video 8. The patch was firmly attached to the heart and completely covered the infarcted area.

Supplementary Video 9. The patch was adhered to the heart using fibrin glue.
