# Supplementary material for: Bioactive Magnesium Silicate Activating Myocardial Energy Metabolism For Infarcted Myocardium Repair
Source: Exploration (Beijing). 2026 Apr 13;6(3):70161. doi: 10.1002/exp2.70161 (PMC13317557; doi:10.1002/exp2.70161)
Supplement: Supplementary file 2 — Supporting File 2: exp270161‐sup‐0002‐Supp‐Data1.docx. [file EXP2-6-70161-s001.docx]

**Supplementary Data 1.** The typical design of bioenergy-activating biomaterials in tissue regeneration.

| **Design** | **Mechanism** | **Ref.** |
| --- | --- | --- |
| The bioink based on magnesium silicate with different morphologies | Chemical cue (ions) + physical cues (the morphology of magnesium silicate adjusted the mitochondria-targeted effects and the dynamic stiffness of the bioink) | This work |
| Microgels containing Mg^2+^ | Chemical cue (ions) | 1 |
| Copper-loaded milk-protein derived microgel | Chemical cue (ions) | 2 |
| Bioenergetic metabolism modulatory MOTS-c (mitochondrial open reading frame of 12S ribosomal RNA type-c) peptide hydrogel | Chemical cue (peptide) | 3 |
| Polygallic acid-manganese nanoparticles | Chemical cue ( the electron exchange between polygallic acid and manganese ions) | 4 |
| Extracellular matrix stiffness as an energy metabolism regulator | Physical cue (the stiffness of extracellular matrix) | 5 |
| A PDMS surface containing micropatterned grooves with a spacing of 1 µm and a depth of 250 or 500 nm | Physical cue (surface morphology) | 6 |
| Plated cells on stiff (collagen-coated glass) and soft elastic collagen substrates (storage modulus (G′) = 16.1 Pa, loss modulus (G′′) = 2.7 Pa) | Physical cue (the stiffness of extracellular matrix) | 7 |

**References:**

1. Lin, S. et al. Orchestration of energy metabolism and osteogenesis by Mg2+ facilitates low-dose BMP-2-driven regeneration. Bioactive Materials 18, 116-127, doi:10.1016/j.bioactmat.2022.03.024 (2022) .

2. Hong, X. et al. Copper-loaded Milk-Protein Derived Microgel Preserves Cardiac Metabolic Homeostasis After Myocardial Infarction. Advanced Science, doi:10.1002/advs.202401527 (2024).

3. Zhang, Y. et al. Bioenergetic Metabolism Modulatory Peptide Hydrogel for Cardiac Protection and Repair After Myocardial Infarction. Advanced Functional Materials 34, doi:10.1002/adfm.202312772 (2024).

4. Chen, Q. et al. Mitochondrial-Targeted Metal-Phenolic Nanoparticles to Attenuate Intervertebral Disc Degeneration: Alleviating Oxidative Stress and Mitochondrial Dysfunction. Acs Nano 18, 8885-8905, doi:10.1021/acsnano.3c12163 (2024).

5. Na, J. et al. Extracellular matrix stiffness as an energy metabolism regulator drives osteogenic differentiation in mesenchymal stem cells. Bioactive Materials 35, 549-563, doi:10.1016/j.bioactmat.2024.02.003 (2024).

6. Singh, A. V. et al. Astrocytes Increase ATP Exocytosis Mediated Calcium Signaling in Response to Microgroove Structures. Scientific Reports 5, doi:10.1038/srep07847 (2015).

7. Park, J. S. et al. Mechanical regulation of glycolysis via cytoskeleton architecture. Nature 578, 621-+, doi:10.1038/s41586-020-1998-1 (2020).
