# Supplementary material for: Bioactive Magnesium Silicate Activating Myocardial Energy Metabolism For Infarcted Myocardium Repair
Source: Exploration (Beijing). 2026 Apr 13;6(3):70161. doi: 10.1002/exp2.70161 (PMC13317557; doi:10.1002/exp2.70161)
Supplement: Supplementary file 3 — Supporting File 3: exp270161‐sup‐0003‐Supp‐Data2.docx. [file EXP2-6-70161-s004.docx]

**Supplementary Data 2.** The typical design of bioenergy-activating biomaterials in tissue regeneration.

| **Strategies for treatment of MI and approaches for evaluating cardiac function** | **Mechanism** | **MI repair effects** | **Ref.** |
| --- | --- | --- | --- |
| The 3D bioprinted 2NT-GelMA cardiac patch was implanted on the heart after MI, followed by echocardiography at 4 weeks after transplantation. | Bioenergy activation | Fractional shortening (FS) 11.1%↑, Ejection fraction (EF) 16.6%↑ | This work |
| Triboelectric nanogenerator conductive cardiac patch was implanted on the heart after MI, followed by echocardiography at 4 weeks after transplantation. | Conductivity | FS 14.7%↑, EF 22.3%↑ | 1 |
| Elastin-gelatin-carbon nanotube cardiac patch was implanted on the heart after MI, and echocardiography was performed at 4 weeks post-transplantation. | Conductivity | FS 11%↑, EF 13%↑ (scaffold only)  FS 12%↑, EF 20%↑ (CMs + scaffold ) | 2 |
| AAV serotype 6-microRNA-199a was delivered by direct intramyocardial injections after MI, and cardiac magnetic resonance imaging was performed at 4 weeks after injection. | MicroRNA delivery | EF < 10%↑ | 3 |
| The AAV9-Sav-short hairpin RNA which could knock down the Hippo pathway gene Salvador (Sav) was delivered into border zone cardiomyocytes via catheter-mediated subendocardial injection, and echocardiography was performed at 20, 40, 60, and 90 days after injection. | Gene therapy | EF < 5%↑ (34 days), EF < 10%↑ (54 days), EF < 10%↑ (74 days), EF 8%↑ (104 days) | 4 |
| A lipid nanoparticle-based long non-coding RNA (Tcf21 antisense RNA inducing demethylation) was delivered into the heart after MI, and echocardiography was performed at 4 weeks post-injection. | RNA delivery | FS < 0%↑, EF < 0%↑ | 5 |
| Intracoronary antegrade injection of recombinant human agrin after MI, and cardiac magnetic resonance imaging was performed at 4 weeks post-treatment. | The extracellular matrix protein agrin delivery | EF < 10%↑ | 6 |
| Thymosin β4-gelatin microspheres and hiPSC-CMs were injected (10 injections) into the center and border zone of infarcted heart, and cardiac magnetic resonance imaging was performed at 4 weeks post-treatment. | Thymosin β4 delivery | EF < 10%↑ | 7 |
| A nitrate-functionalized patch (local nitric oxide delivery) was transplanted on the heart after MI, and echocardiography was performed at 4 weeks post-transplantation. | Nitric oxide delivery | FS < 10%↑, EF < 16%↑ | 8 |
| A prevascularized cardiac stromal cell patch endothelialized microvessels and encapsulated with therapeutic cardiosphere-derived stromal cells was implanted on the heart after MI, followed by echocardiography at 4 weeks after transplantation. | Microvessels and therapeutic cells | EF < 5%↑ | 9 |
| A patch consisting of decellularized porcine myocardial extracellular matrix and cell-secreted regenerative factors was implanted into the heart after MI, and echocardiography was conducted 7 days post-transplantation. | Cell secreted factors delivery | FS < 5%↑, EF < 2%↑ (7 days) | 10 |
| Following MI, 25 mL of ion cocktail-containing saline was intravenously injected every other day (8 times in total). | Injection of ion cocktail-containing saline | FS 9%↑, EF 12%↑ | 11 |

**References:**

1. Qiu, R. et al. E-cardiac patch to sense and repair infarcted myocardium. Nature communications 15, 4133-4133, doi:10.1038/s41467-024-48468-x (2024).

2. Wang, L. et al. Injectable and conductive cardiac patches repair infarcted myocardium in rats and minipigs. Nature Biomedical Engineering 5, 1157-1173, doi:10.1038/s41551-021-00796-9 (2021).

3. Gabisonia, K. et al. MicroRNA therapy stimulates uncontrolled cardiac repair after myocardial infarction in pigs. Nature 569, 418-+, doi:10.1038/s41586-019-1191-6 (2019).

4. Liu, S. et al. Gene therapy knockdown of Hippo signaling induces cardiomyocyte renewal in pigs after myocardial infarction. Science Translational Medicine 13, doi:10.1126/scitranslmed.abd6892 (2021).

5. Zhu, D. et al. Intrapericardial long non-coding RNA-Tcf21 antisense RNA inducing demethylation administration promotes cardiac repair. European Heart Journal 44, doi:10.1093/eurheartj/ehad114 (2023).

6. Baehr, A. et al. Agrin Promotes Coordinated Therapeutic Processes Leading to Improved Cardiac Repair in Pigs. Circulation 142, 868-881, doi:10.1161/circulationaha.119.045116 (2020).

7. Tan, S. H. et al. Thymosin β4 increases cardiac cell proliferation, cell engraftment, and the reparative potency of human induced-pluripotent stem cell-derived cardiomyocytes in a porcine model of acute myocardial infarction. Theranostics 11, 7879-7895, doi:10.7150/thno.56757 (2021).

8. Zhu, D. et al. Nitrate-functionalized patch confers cardioprotection and improves heart repair after myocardial infarction via local nitric oxide delivery. Nature Communications 12, doi:10.1038/s41467-021-24804-3 (2021).

9. Su, T. et al. Cardiac Stromal Cell Patch Integrated with Engineered Microvessels Improves Recovery from Myocardial Infarction in Rats and Pigs. Acs Biomaterials Science & Engineering 6, 6309-6320, doi:10.1021/acsbiomaterials.0c00942 (2020).

10. Huang, K. et al. An off-the-shelf artificial cardiac patch improves cardiac repair after myocardial infarction in rats and pigs. Science Translational Medicine 12, doi:10.1126/scitranslmed.aat9683 (2020).

11. Que, Y. et al. Ion cocktail therapy for myocardial infarction by synergistic regulation of both structural and electrical remodeling. Exploration (Beijing, China) 4, 20230067-20230067, doi:10.1002/exp.20230067 (2024).
